# Genetic Variation and Population Structure in Native Americans

**DOI:** 10.1371/journal.pgen.0030185

**Published:** 2007-11-23

**Authors:** Sijia Wang, Cecil M Lewis, Mattias Jakobsson, Sohini Ramachandran, Nicolas Ray, Gabriel Bedoya, Winston Rojas, Maria V Parra, Julio A Molina, Carla Gallo, Guido Mazzotti, Giovanni Poletti, Kim Hill, Ana M Hurtado, Damian Labuda, William Klitz, Ramiro Barrantes, Maria Cátira Bortolini, Francisco M Salzano, Maria Luiza Petzl-Erler, Luiza T Tsuneto, Elena Llop, Francisco Rothhammer, Laurent Excoffier, Marcus W Feldman, Noah A Rosenberg, Andrés Ruiz-Linares

**Affiliations:** 1 The Galton Laboratory, Department of Biology, University College London, London, United Kingdom; 2 Department of Human Genetics, University of Michigan, Ann Arbor, Michigan, United States of America; 3 Center for Computational Medicine and Biology, University of Michigan, Ann Arbor, Michigan, United States of America; 4 Department of Biological Sciences, Stanford University, Stanford, California, United States of America; 5 Computational and Molecular Population Genetics Lab, University of Bern, Bern, Switzerland; 6 Laboratorio de Genética Molecular, Universidad de Antioquia, Medellín, Colombia; 7 Center for Neurobehavioral Genetics, University of California Los Angeles, Los Angeles, United States of America; 8 Laboratorios de Investigación y Desarrollo, Facultad de Ciencias y Filosofía, Universidad Peruana Cayetano Heredia, Lima, Perú; 9 Facultad de Medicina, Universidad Peruana Cayetano Heredia, Lima, Perú; 10 Department of Anthropology, University of New Mexico, Albuquerque, New Mexico, United States of America; 11 Département de Pédiatrie, CHU Sainte-Justine, Université de Montréal, Montréal, Quebec, Canada; 12 School of Public Health, University of California Berkeley, Berkeley, California, United States of America; 13 Public Health Institute, Oakland, California, United States of America; 14 Escuela de Biología, Universidad de Costa Rica, San José, Costa Rica; 15 Departamento de Genética, Instituto de Biociências, Universidade Federal do Rio Grande do Sul, Porto Alegre, Rio Grande do Sul, Brazil; 16 Departamento de Genética, Universidade Federal do Paraná, Curitiba, Paraná, Brazil; 17 Programa de Genética Humana, Instituto de Ciencias Biomédicas, Facultad de Medicina, Universidad de Chile, Santiago, Chile; 18 Instituto de Alta Investigación, Universidad de Tarapacá, Arica, Chile; The Wellcome Trust Sanger Institute, United Kingdom

## Abstract

We examined genetic diversity and population structure in the American landmass using 678 autosomal microsatellite markers genotyped in 422 individuals representing 24 Native American populations sampled from North, Central, and South America. These data were analyzed jointly with similar data available in 54 other indigenous populations worldwide, including an additional five Native American groups. The Native American populations have lower genetic diversity and greater differentiation than populations from other continental regions. We observe gradients both of decreasing genetic diversity as a function of geographic distance from the Bering Strait and of decreasing genetic similarity to Siberians—signals of the southward dispersal of human populations from the northwestern tip of the Americas. We also observe evidence of: (1) a higher level of diversity and lower level of population structure in western South America compared to eastern South America, (2) a relative lack of differentiation between Mesoamerican and Andean populations, (3) a scenario in which coastal routes were easier for migrating peoples to traverse in comparison with inland routes, and (4) a partial agreement on a local scale between genetic similarity and the linguistic classification of populations. These findings offer new insights into the process of population dispersal and differentiation during the peopling of the Americas.

## Author Summary

Studies of genetic variation have the potential to provide information about the initial peopling of the Americas and the more recent history of Native American populations. To investigate genetic diversity and population relationships in the Americas, we analyzed genetic variation at 678 genome-wide markers genotyped in 29 Native American populations. Comparing Native Americans to Siberian populations, both genetic diversity and similarity to Siberians decrease with geographic distance from the Bering Strait. The widespread distribution of a particular allele private to the Americas supports a view that much of Native American genetic ancestry may derive from a single wave of migration. The pattern of genetic diversity across populations suggests that coastal routes might have been important during ancient migrations of Native American populations. These and other observations from our study will be useful alongside archaeological, geological, and linguistic data for piecing together a more detailed description of the settlement history of the Americas.

## Introduction

Patterns of genetic diversity and population structure in human populations constitute an important foundation for many areas of research in human genetics. Most noticeably, they provide an invaluable source of data for inferences about human evolutionary history [1–3]. In addition, the distribution of genetic variation informs the design and interpretation of studies that search for genes that confer an increased susceptibility to disease [4–6].

Recent genomic studies have produced detailed genome-wide descriptions of genetic diversity and population structure for a wide variety of human populations, both at the global level [7–19] and for individual geographic regions, including East Asia [20], Europe [21,22], and India [23]. Here we report the first such analysis of indigenous populations from the American landmass, using 678 microsatellites genotyped in 530 individuals from 29 Native American populations. The study is designed to investigate several questions about genetic variation in Native Americans: what records of the original colonization from Siberia are retained in Native American genetic variation? What geographic routes were taken in the Americas by migrating peoples? What is the genetic structure of Native American populations? To what extent does genetic differentiation among populations parallel the differentiation of Native American languages? In addressing these questions, our analyses identify several surprising features of genetic variation and population history in the Americas.

## Results

**Figure 1:**
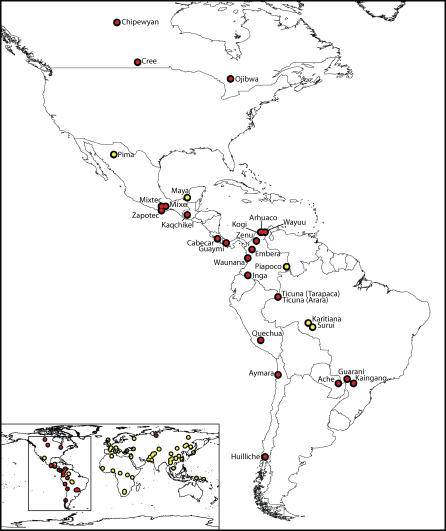
Populations Included in This Study The world map shows the 78 populations investigated in the combined dataset, with the locations of the 29 populations studied in the Americas shown in detail in the larger map. The 25 newly examined populations, including the Siberian Tundra Nentsi, are marked in red, and the previously genotyped HGDP-CEPH populations are marked in yellow.

We collected genome-wide microsatellite genotype data for 751 autosomal markers in 422 individuals from 24 Native American populations spanning ten countries and seven linguistic “stocks” (Tables S1 and S2). We also collected data on 14 individuals from a Siberian population, Tundra Nentsi. To enable comparisons with data previously reported in the worldwide collection of populations represented by the Human Genome Diversity Project–Centre d'Etude du Polymorphisme Humain (HGDP–CEPH) cell line panel [7,11,13], data analysis was restricted to 678 loci typed across all populations (see Methods). The combined dataset contains genotypes for 1,484 individuals from 78 populations, including 29 Native American groups and two Siberian groups (Figure 1).

### Genetic Diversity

**Figure 2:**
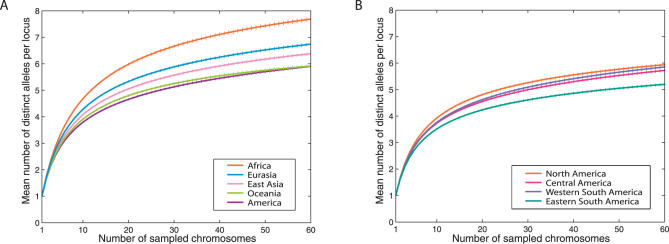
The Mean and Standard Error Across 678 Loci of the Number of Distinct Alleles as a Function of the Number of Sampled Chromosomes (A) Geographic regions worldwide. (B) Subregions within the Americas. For a given locus, region, and sample size *g*, the number of distinct alleles averaged over all possible subsamples of *g* chromosomes from the given region is computed according to the rarefaction method [24,25]. For each sample size *g*, loci were considered only if their sample sizes were at least *g* in each geographic region. Error bars denote the standard error of the mean across loci.

We compared levels of genetic diversity across geographic regions worldwide (Table 1). A serial founding African-origin model of human evolution [10,11]—in which each successive human migration involved only a subset of the genetic variation available at its source location, and in which the Bering Strait formed the only entry point to the American landmass—predicts reduced genetic diversity in Native Americans compared to other populations, as well as a north-to-south decline in genetic diversity among Native American populations. Indeed, Native Americans were found to have lower genetic diversity, as measured by heterozygosity, than was seen in populations from other continents (Table 1). Additionally, applying a sample size-corrected measure of the number of distinct alleles in a population [24,25], Native Americans had fewer distinct alleles per locus compared to populations in other geographic regions (Figure 2A). Among Native American populations, the highest heterozygosities were observed in the more northerly populations, and the lowest values were seen in South American populations (Table 2). The lowest heterozygosities of any populations worldwide occurred in isolated Amazonian and eastern South American populations, such as Surui and Ache. More generally, heterozygosity was reduced in eastern populations from South America compared to western populations (Table 1, *p* = 0.02, Wilcoxon rank sum test). Eastern South American populations also had fewer distinct alleles per locus than populations elsewhere in the Americas (Figure 2B).

**Table 1:**
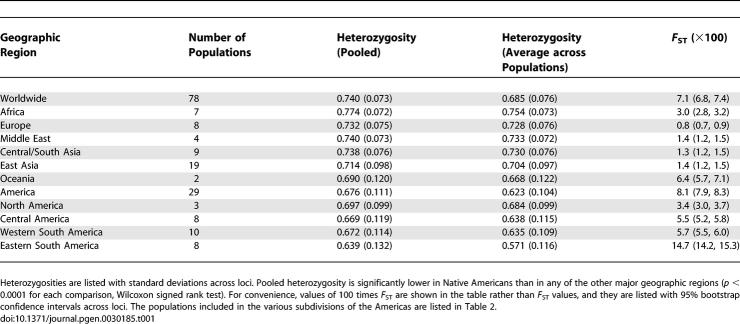
Heterozygosity and *F*_ST_ (×100) for Various Geographic Regions

**Table 2:**
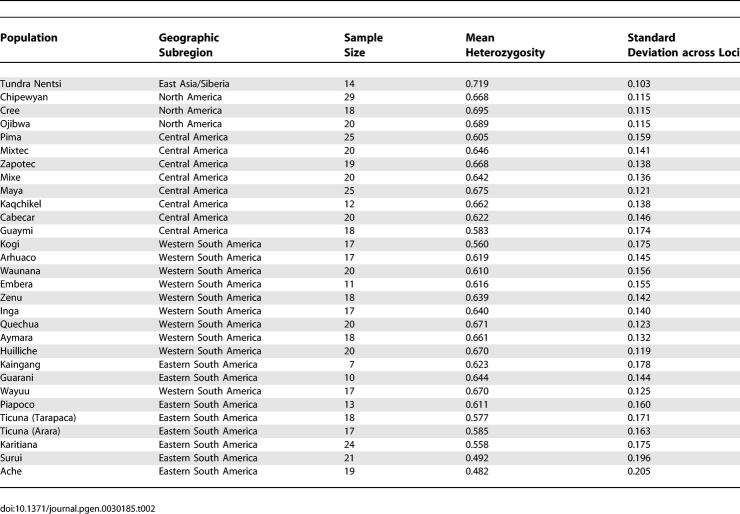
Heterozygosity for Newly Sampled Populations and for the Five Previously Sampled Native American Populations (Pima, Maya, Piapoco, Karitiana, and Surui)

**Figure 3:**
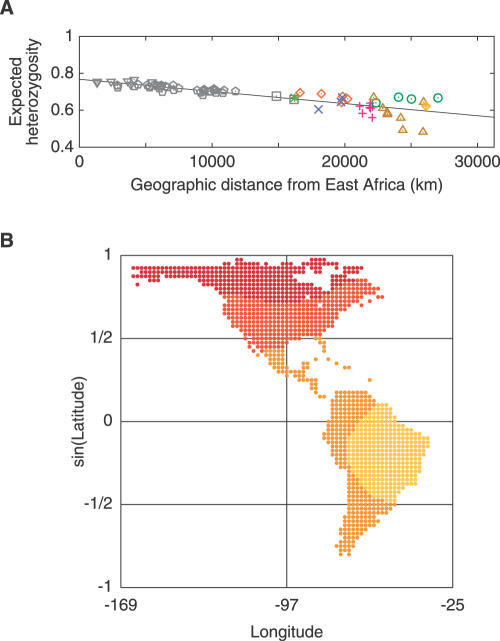
Heterozygosity in Relation to Geography (A) Relationship between heterozygosity and geographic distance from East Africa. Populations in Sub-Saharan Africa and Oceania are marked with gray triangles and squares, respectively, and the remaining non-American populations from Europe, Asia, and northern Africa are marked with gray pentagons. Within the Americas, populations are color-coded and symbol-coded by language stock (see Figure 8). Denoting heterozygosity by *H* and geographic distance in thousands of kilometers by *D*, the regression line for the graph is *H* = 0.7679 − 0.00658*D*, with correlation coefficient −0.862. (B) The fit of a linear decline of heterozygosity with increasing distance from a putative source, considering Native American populations only. The color of a point indicates a correlation coefficient *r* between expected heterozygosity and geographic distance from the point, with darker colors denoting more strongly negative correlations. Across the Americas, the correlation ranges from −0.436 to 0.575, and color bins are set to equalize the number of points drawn in the four colors. From darkest to lightest, the four colors represent points with correlations in (−0.436, −0.424), (−0.424, −0.316), (−0.316, 0.494), and (0.494, 0.575), respectively.

Assuming a single source for a collection of populations, the serial founding model predicts a linear decline of genetic diversity with geographic distance from the source location [11,26]. Such a pattern is observed at the worldwide level, as a linear reduction of heterozygosity is seen with increasing distance from Africa, where distance to Native American populations is measured via a waypoint near the Bering Strait (Figure 3A). To investigate the source location for Native Americans, we considered only the Native American data and allowed the source to vary, measuring the correlation of heterozygosity with distance from putative points of origin. Consistent with the founding from across the Bering Strait, the correlation of heterozygosity with geographic distance from a hypothesized source location had the most strongly negative values (*r* = −0.436) when the source for Native Americans was placed in the northernmost part of the American landmass (Figure 3B). The smallest value for the correlation coefficient was seen at 55.6°N 98.8°W, in central Canada, but as a result of relatively sparse sampling in North America, all correlations in the quartile with the smallest values, plotted in the darkest shade in Figure 3B, were within a narrow range (−0.436 to −0.424).

**Figure 4:**
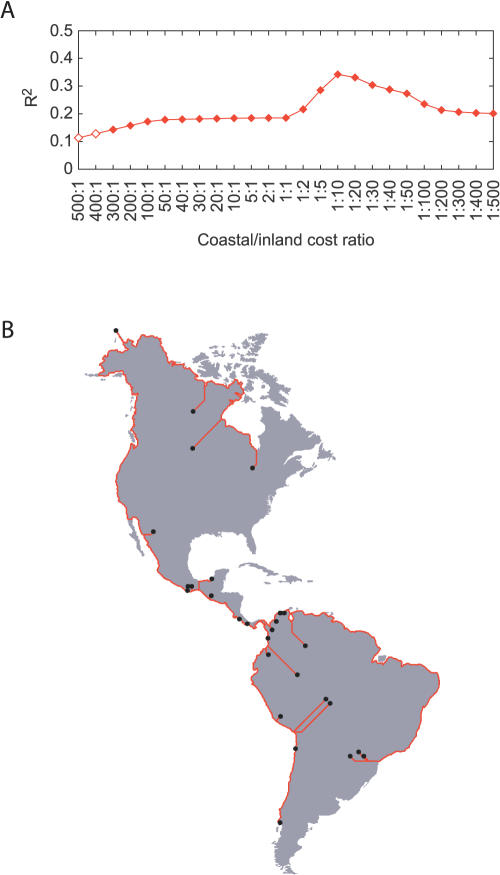
Heterozygosity and Least-Cost Paths in a Coastal Migration Scenario (A) *R* ^2^ (square of the correlation) between heterozygosity *H* and effective geographic distance (least-cost distance), assuming differential permeability of coastal regions compared to inland regions. Correlations significant at the 0.05 level are indicated by closed symbols, and those that are not significant are indicated by open symbols. (B) Least-cost routes for the scenario with 1:10 coastal/inland cost ratio.

One way to examine the support for particular colonization routes within the American landmass is to determine if a closer relationship between heterozygosity and geography is observed when “effective” geographic distances are computed along these routes, rather than along shortest-distance paths. Using *PATHMATRIX* [27] to take the precise locations of continental boundaries into consideration in effective geographic distance calculations (see Methods)—rather than using a waypoint approach [11] to measure distance—does not substantially alter the correlation of heterozygosity with distance from the Bering Strait (*r* = −0.430, 1:1 coastal/inland cost ratio in Figure 4A). However, when coastlines are treated as preferred routes of migration in comparison with inland routes, the percent of variance in heterozygosity explained by effective distance increases to 34% (*r* = −0.585 for a coastal/inland cost ratio of 1:10 in Figure 4A). In contrast, all scenarios tested that had coastal/inland cost ratios greater than 1 explain a smaller proportion of the variance in heterozygosity than do the scenarios with coastal/inland cost ratio of 1 or less.

The preferred routes in the optimal scenario of a 1:10 coastal/inland cost ratio include a path to the Ache, Guarani, and Kaingang populations that travels around northern South America (Figure 4B). With these three populations excluded, the role of coastlines is almost unchanged (Figure S1), and a 1:10 ratio continues to explain the largest fraction of variation in heterozygosity (*r* = −0.595). Applying a reduced cost only to the Pacific coast, a preference is still seen for ratios slightly less than 1 compared to ratios greater than 1, and the scenario producing the closest fit is a 1:2 ratio (Figure S2). A stronger preference for a Pacific coastal route was observed excluding from the computations the Chipewyan, Cree, and Ojibwa populations, three groups that follow an Arctic route in Figure 4B, or excluding Ache, Guarani, and Kaingang in addition to Chipewyan, Cree, and Ojibwa (Figure S3). We did not find a closer fit of heterozygosity and effective distance assuming a reduced cost for travel along major rivers, and indeed we observed that a higher cost for riverine routes was preferred (Figure S3).

### Intercontinental Population Structure

To investigate population structure at the worldwide level, we used unsupervised model-based clustering as implemented in the *STRUCTURE* program [28,29]. Using *STRUCTURE*, we applied a mixture model that allows for allele frequency correlation across a set of *K* genetic clusters, with respect to which individual membership coefficients are estimated (see Methods).

**Figure 5:**
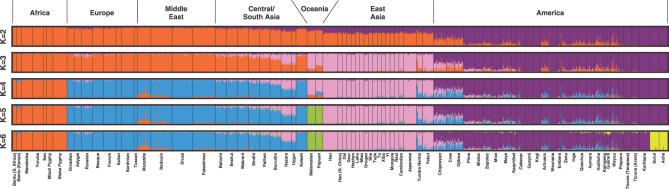
Unsupervised Analysis of Worldwide Population Structure The number of clusters in a given plot is indicated by the value of *K*. Individuals are represented as thin vertical lines partitioned into segments corresponding to their membership in genetic clusters indicated by the colors.

As has been observed previously [7,9,13,16,23], cluster analysis with worldwide populations identifies a major genetic cluster corresponding to Native Americans (Figure 5), indicating an excess similarity of individual genomes within the Americas compared to genomes in other regions. Inclusion of the Native American data collected here did not substantially alter the clusters identified in previous analyses. When the genotypes were analyzed using a model with five clusters, the clusters corresponded to Sub-Saharan Africa, Eurasia west of the Himalayas, Asia east of the Himalayas, Oceania, and the Americas. For a model with six clusters, the sixth cluster corresponded mainly to the isolated Ache and Surui populations from South America. Almost no genetic membership from the cluster containing Africans and a relatively small amount of membership from the cluster containing Europeans were detected in the Native Americans, indicating that with relatively few exceptions, the samples examined here represent populations that have experienced little recent European and African admixture.

**Figure 6:**
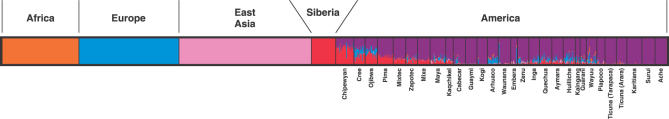
Supervised Population Structure Analysis, Using Five Clusters, Four of Which Were Forced to Correspond to Africans, Europeans, East Asians Excluding Siberians, and Siberians

To search for signals of similarity to Siberians in the Native American populations, we used a supervised cluster analysis [28,29] in which Native Americans were distributed over five clusters (Figure 6). Four of these clusters were forced to correspond to Africans, Europeans, East Asians excluding Siberians, and Siberians (Tundra Nentsi and Yakut), and the fifth cluster was not associated with any particular group a priori. Most Native American individuals were seen to have majority membership in this fifth cluster, and considering their estimated membership in the remaining clusters, Native Americans were genetically most similar to Siberians. A noticeable north-to-south gradient of decreasing similarity to Siberians was observed, as can be seen in the declining membership in the red cluster from left to right in Figure 6. Genetic similarity to Siberia is greatest for the Chipewyan population from northern Canada and for the more southerly Cree and Ojibwa populations. Detectable Siberian similarity is visible to a greater extent in Mesoamerican and Andean populations than in the populations from eastern South America.

### Intracontinental Population Structure

The level of population structure observed among Native Americans, as determined using *F*_ST_ [30], was 0.081, exceeding that of other geographic regions (Table 1). Comparing regions within the Americas, the highest *F*_ST_ value was observed in eastern South America, with intermediate values occurring in western South America and Central America and with the smallest value occurring in North America (Table 1). These results are compatible with the lower overall level of Native American genetic variation, particularly in eastern South America, as the mathematical connection between heterozygosity and *F*_ST_ predicts that low heterozygosities will tend to produce higher *F*_ST_ values [11,31–33].

**Figure 7:**
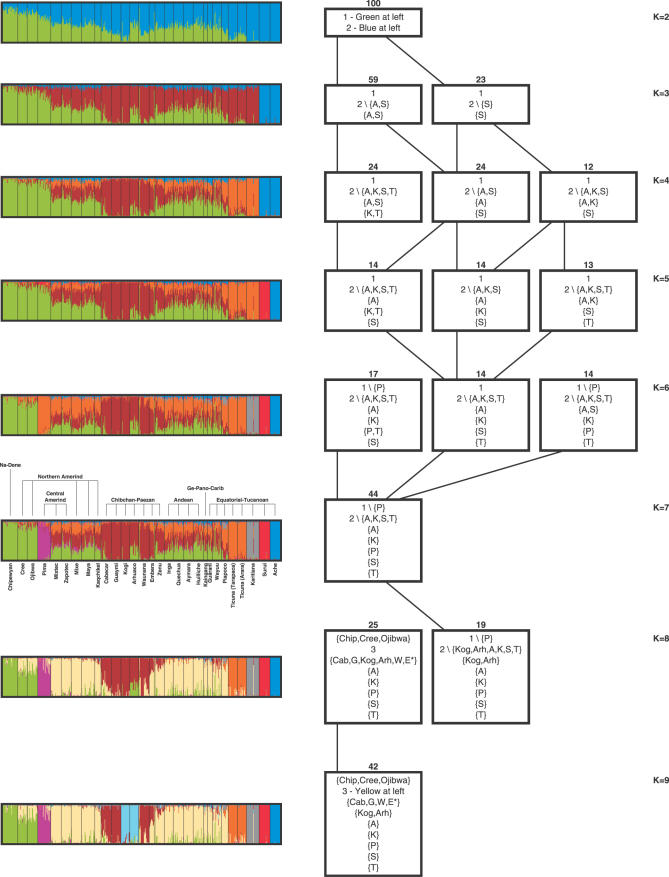
Unsupervised Analysis of Native American Population Structure The colored plots at the left show the estimated population structure of Native Americans, obtained using *STRUCTURE*. The number of clusters in a given plot is indicated by the value of *K* on the right side of the figure. Next to the *K* = 7 plot, the population names and the major language stocks of the populations are also displayed. The left-to-right order of the individuals is the same in all plots. The diagram on the right summarizes the outcomes of 100 replicate *STRUCTURE* runs for each of several values of *K*. Each row represents a value of *K*, and within each row, each box represents a clustering solution that appeared at least 12 times in 100 replicates (see Methods). The number of appearances of a solution is listed above the box, and the boxes are arrayed from left to right in decreasing order of the frequencies of the solutions to which they correspond. The *DISTRUCT* plot shown on the left corresponds to the leftmost box on the right side of the figure. An approximate description of the clusters is located inside the box, with each row in the box representing a different cluster. The numbers 1, 2, and 3 are used to refer to the green cluster in the *K* = 2 *DISTRUCT* plot, the blue cluster in the *K* = 2 *DISTRUCT* plot, and the yellow cluster in the *K* = 9 *DISTRUCT* plot, respectively. The following population abbreviations are also used: A, Ache; Arh, Arhuaco; Cab, Cabecar; Chip, Chipewyan; E, Embera; G, Guaymi; K, Karitiana; Kog, Kogi; P, Pima; S, Surui; T, Ticuna (both Ticuna groups combined); W, Waunana. Clusters are indicated using set notation; for example {A} represents a cluster containing Ache only, and 2\{A,S} represents a cluster that corresponds to cluster 2 (the blue cluster for *K* = 2), excluding Ache and Surui. An asterisk indicates approximately 50% membership of a population in a cluster. A line is drawn from a box representing a solution with *K* clusters to a box representing a solution with *K*+1 clusters if the solution with *K*+1 clusters refines the solution with *K* clusters—that is, if all of the clusters in the solution with *K*+1 clusters subdivide the clusters in the solution with *K* clusters. In case of ties for the highest-frequency solution (*K* = 4 and *K* = 5), boxes are oriented in order to avoid the crossing of lines between them.

Applying unsupervised model-based clustering [28,29] to the Native Americans, considerable population substructure is detectable (Figure 7). For a model with two clusters, one cluster corresponds largely to the northernmost populations, while the other corresponds to populations from eastern South America; the remaining populations are partitioned between these two clusters, with greater membership of the more northerly populations in the “northern” cluster. As the number of clusters is increased, the least genetically variable groups form distinctive clusters (for example, the Ache, Karitiana, and Surui populations). However, variation exists across replicates in the nature of the partitioning, and to illustrate the range of solutions observed, Figure 7 summarizes each clustering solution that was seen in at least 12% of replicate analyses for each *K* from two to nine. These summaries indicate that the main clustering solutions with a given *K* “refine” the partitions observed with *K* − 1 clusters, in the sense that each of the *K* clusters is either identical to, or is a subset of, one of the *K* − 1 clusters. A likely explanation for the multimodality is the presence of several population subgroups that are roughly equally likely to form individual clusters. For small *K*, not enough slots are available, and only when *K* is sufficiently large is each of these groups able to occupy its own cluster.

**Figure 8:**
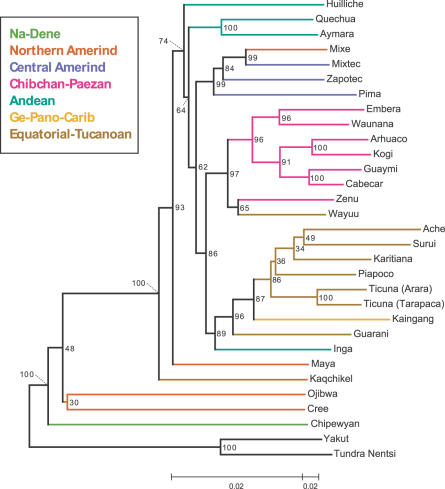
Neighbor-Joining Tree of Native American Populations Each language stock is given a color, and if all populations subtended by an edge belong to the same language stock, the clade is given the color that corresponds to that stock. Branch lengths are scaled according to genetic distance, but for ease of visualization, a different scale is used on the left and right sides of the middle tick mark at the bottom of the figure. The tree was rooted along the branch connecting the Siberian populations and the Native American populations, and for convenience, the forced bootstrap score of 100% for this rooting is indicated twice.

For *K* = 7, a relatively stable clustering solution is observed, appearing in 44 of 100 replicates (compared to seven of 100 for the next most frequently observed solution). This clustering solution has distinctive clusters for three of the smallest and least genetically variable groups in the sample—Karitiana and Surui from Brazil, and Ache from Paraguay. Two separate samples from the Amazonian Ticuna group of Colombia form the basis for a cluster, as does the Pima group from Mexico. The remaining two clusters include one centered on the North American groups and one centered on the Chibchan–Paezan language stock from Central and South America. The cluster containing Chibchan–Paezan populations—the only cluster at *K* = 7 that corresponds well to a major language stock—separates into two subclusters when *K* is increased to nine. Despite the large geographic distance between Mesoamerica and the Andes, Mesoamerican populations (Mixtec, Zapotec, Mixe, and Maya from Mexico and Kaqchikel from Guatemala) and Andean populations (Inga from Colombia, Quechua from Peru, and Aymara and Huilliche from Chile) have similar estimated membership across clusters when *K* = 7, and together with five additional populations (Zenu, Wayuu, and Piapoco from Colombia, and Kaingang and Guarani from Brazil), they comprise a single cluster when *K* = 9.

### Genes and Languages

We compared the classification of the populations into linguistic “stocks” [34,35] (Table S2) with their genetic relationships as inferred on a neighbor-joining tree constructed from Nei genetic distances [36] between pairs of populations (Figure 8). As the use of a single-family grouping (Amerind) of all languages not belonging to the Na–Dene or Eskimo–Aleutian families is controversial [37], we focused our analysis on the taxonomically lower level of linguistic stocks.

In the neighbor-joining tree (Figure 8), a reasonably well-supported cluster (86%) includes all non-Andean South American populations, together with the Andean-speaking Inga population from southern Colombia. Within this South American cluster, strong support exists for separate clustering of Chibchan–Paezan (97%) and Equatorial–Tucanoan (96%) speakers (except for the inclusion of the Equatorial–Tucanoan Wayuu population with its Chibchan–Paezan geographic neighbors, and the inclusion of Kaingang, the single Ge–Pano–Carib population, with its Equatorial–Tucanoan geographic neighbors). Within the Chibchan–Paezan and Equatorial–Tucanoan subclusters several subgroups have strong support, including Embera and Waunana (96%), Arhuaco and Kogi (100%), Cabecar and Guaymi (100%), and the two Ticuna groups (100%). When the tree-based clustering is repeated with alternate genetic distance measures, despite the high Mantel correlation coefficients [38] between distance matrices (0.98, 0.98, and 0.99 for comparisons of the Nei and Reynolds matrices, the Nei and chord matrices, and the Reynolds and chord matrices, respectively), higher-level groupings tend to differ slightly or to have reduced bootstrap support (Figures S4 and S5). However, local groupings such as Cabecar and Guaymi, Arhuaco and Kogi, Aymara and Quechua, and Ticuna (Arara) and Ticuna (Tarapaca) continue to be supported (100%). This observation of strongly supported genetic relationships for geographically proximate linguistically similar groups coupled with smaller support at the scale of major linguistic groupings is also seen in Native American mitochondrial data [39].

To more quantitatively test the correspondence of genetic and linguistic variation in the Americas, we computed the Mantel correlation of genetic and linguistic distances (Table 3). Nei's *D*_a_ distance [36] was used for the genetic computations, and linguistic distances were measured along a discrete scale (see Methods). Considering all of the Native American populations and treating all linguistic stocks as equidistant (Table S3), the Mantel correlation of Nei genetic distance with linguistic distance is small (*r* = 0.04). The correlation is also small when using between-stock linguistic distance measures (Tables S4–S11) that make use of shared etymologies identified by Greenberg [34]. For two ways of computing linguistic distance, using the Dice and Jaccard indices (see Methods), respectively, the correlations are *r* = −0.01 and *r* = −0.02. When the effects of geography are controlled, or when stocks are excluded from the computation individually, the partial correlations of linguistic and genetic distance [40] remain low.

**Table 3:**
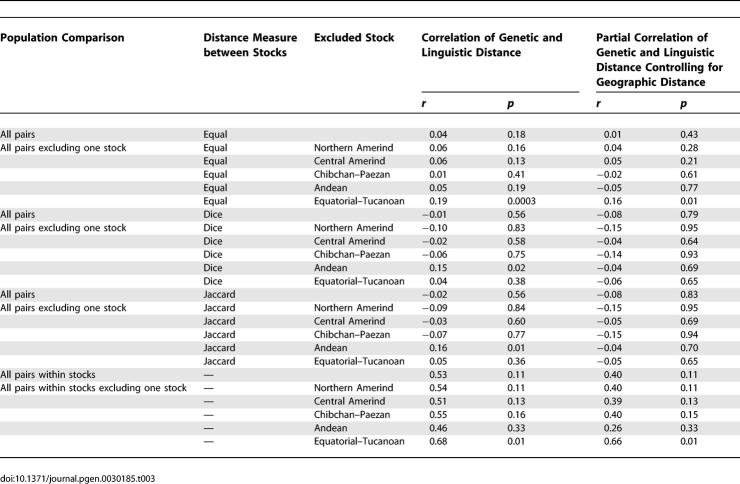
Correlation of Genetic and Linguistic Distances

A potential explanation for the low correlation coefficients—suggested by the apparent genetic and linguistic correspondence in the neighbor-joining tree for closely related groups—is that sizeable correlation between genetic and linguistic distance may exist only below a certain level of linguistic distance. Considering genetic and linguistic differentiation only for pairs of populations within linguistic stocks, the correlation of genetic distance and linguistic distance increases (*r* = 0.53). The partial correlation of genetic distance and linguistic distance remains fairly high when the effect of geographic distance is controlled (*r* = 0.40), although 11% of random matrix permutations produce higher values (Table 3).

By excluding language stocks from the computation individually, it is possible to investigate the extent to which individual linguistic stocks are responsible for the within-stock correlation of genetic and linguistic distance. When the Equatorial–Tucanoan stock is excluded, the correlation increases to 0.68, and the partial correlation controlling for geographic distance increases to 0.66. Excluding the Andean stock, however, both the correlation and the partial correlation decrease (to 0.46 and 0.26, respectively). Excluding any of the three other stocks for which more than one population is represented (Northern Amerind, Central Amerind, Chibchan–Paezan) does not lead to a sizeable change in either the correlation coefficient (0.54, 0.51, 0.55) or the partial correlation coefficient (0.40, 0.39, 0.40).

### Native American Private Alleles

**Figure 9:**
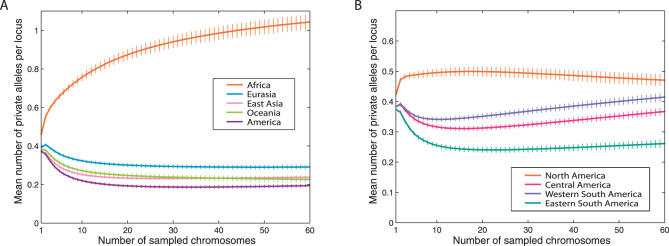
The Mean and Standard Error Across 678 Loci of the Number of Private Alleles as a Function of the Number of Sampled Chromosomes For a given locus, region, and sample size *g*, the number of private alleles in the region—averaging over all possible subsamples that contain *g* chromosomes each from the five regions—is computed according to an extension of the rarefaction method [25]. For each sample size *g*, loci were considered only if their sample sizes were at least *g* in each geographic region. Error bars denote the standard error of the mean across loci.

Considering alleles found only in one major geographic region worldwide, Native Americans have the fewest private alleles (Figure 9A). Private alleles, which lie at the extreme ends of the allele size range more often than expected by chance (*p* < 0.023), usually have low frequencies in the geographic region where they are found (≤13%). Within the Americas, counting alleles private to one of four subregions, northern populations have the most and eastern South American populations have the fewest private alleles, with western South American populations having slightly more than Central American populations (Figure 9B).

**Figure 10:**
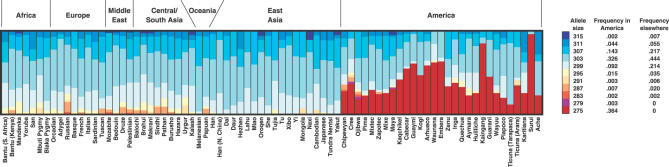
Allele Frequency Distribution at Tetranucleotide Locus D9S1120 For each population the sizes of the colored bars are proportional to allele frequencies in the population, with alleles color-coded as in the legend. Alleles are ordered from bottom to top by increase in size, with the smallest allele, a Native American private allele of size 275, shown in red, and the largest allele, 315, shown in dark blue.

Despite this general lack of high-frequency private alleles, especially in Native Americans, we observed that the only common (>13%) regionally private variant in the worldwide dataset was a Native American private allele. This allele, corresponding to a length of 275 base pairs at locus D9S1120, was found at a frequency of 36.4% in the full Native American sample, and was absent from the other 49 world populations. Allele 275 is the smallest variant observed at the locus and it is present in each of the 29 Native American populations—at frequencies ranging from 11.1% in Ticuna (Tarapaca) to 97.1% in Surui (Figure 10). This allele has now been observed in every Native American population in which the locus has been investigated [41,42], and it has only been seen elsewhere in two populations at the far eastern edge of Siberia [42].

## Discussion

Because of the likely submergence of key archaeological sites along the Pacific coast, the relative absence of a written record, and the comparatively recent time scale of the initial colonization, population-genetic approaches provide a particularly important source of data for the study of Native American population history [43–52]. In this article, building upon recent investigations that have increased the size of Native American genetic datasets beyond classical marker, Y-chromosomal, mitochondrial, and single-gene studies [7,11,13,16,41,53–65], we have examined genome-wide patterns of variation in a dataset that—in terms of total genotypes—represents the largest continent-wide Native American population-genetic study performed to date. Our results have implications for a variety of topics in the demographic history of Native Americans, including (1) the process by which the American landmass was originally populated, (2) the routes taken by the founders during and subsequent to the migration, and (3) the extent to which genes and languages have traveled together during the diversification of Native American populations. We discuss these issues in sequence.

### Genetic Signatures of the Colonization from Siberia

The lower level of genetic diversity observed in the Americas compared to other continental regions is compatible with a reduction in population size associated with a geographically discrete founding, representing one of the most recent in a series of major bottlenecks during human expansions outward from Africa [11]. Gradients of genetic diversity (Figure 3) and decreasing similarity to Siberians (Figure 6) also point to extant Native Americans as the descendants of a colonization process initiated from the northwestern part of the American landmass. An alternative possibility that could produce a genetic diversity gradient—namely, a north-to-south gradient of recent admixture from high-diversity European populations—can be eliminated as a possible explanation given that (1) European admixture is not strongly correlated with distance from the Bering Strait (*r* = −0.135), (2) inclusion of a European admixture covariate in the regression of heterozygosity on distance from the Bering Strait is not supported (*p* = 0.37) and only slightly increases the fit of the regression model (*R* ^2^ = 0.208 compared to *R* ^2^ = 0.182), and (3) the regression of heterozygosity on distance from the Bering Strait does not change substantially when the most highly admixed populations are excluded from the analysis (Table S12). The genetic diversity and population structure gradients—which are generally compatible with principal component maps of allele frequencies at small numbers of classical markers [1,66] and with some analyses of mitochondrial, X-chromosomal, and Y-chromosomal data [67,68]—are more clearly visible in our study of a larger number of loci.

Although gradients of genetic diversity and Siberian similarity constitute major features of the pattern of Native American variation when considering all of the loci together, one important aspect of Native American variation—the distribution of a private allele at locus D9S1120—deviates from the genome-wide pattern and does not show a north-to-south frequency gradient. The geographic distribution of this allele is similar to the distributions of certain mitochondrial and Y-chromosomal variants that are also ubiquitous in the Americas, but that are absent elsewhere or that are found outside the Americas only in extreme northeast Siberia [69–74]. Such distributions are most easily explained by the spatial diffusion of initially rare variants during the colonization of the continent, rather than by continent-wide natural selection or by an origin considerably later than the colonization [42,75,76]. The restricted distribution in Asia of D9S1120 allele 275 and similar Y-chromosomal and mitochondrial variants suggests one of several explanations [42]: the ancestral population that migrated to the Americas may have already acquired a degree of genetic differentiation from other Asian populations [77], descendants of the original Native American founders are no longer present elsewhere in Asia, or these descendants have not yet been genotyped at loci that carry apparently private Native American variants.

The genomic continent-wide patterns observed here can be explained most parsimoniously by a single main colonization event, as proposed by some interpretations of archaeological, mitochondrial, and Y-chromosomal data [67,74,77–83]. In this view, at each step in the migration, a subset of the population splitting off from a parental group moves deeper into the Americas, taking with it a subset of the genetic variation present in the parental population. This scenario would be expected to produce a set of low-diversity populations with distinctive patterns of variation at the far terminus of the migration, such as those we and others [84] observe in the Ache and Surui populations. It can also explain the gradient of Siberian similarity, and the continent-wide distribution of D9S1120 allele 275. Alternatively, similar patterns could result from gene flow across the Bering Strait in the last few thousand years, together with continual interactions between neighbors on both sides of the Bering Strait [47]. It is also possible to envision a series of prehistoric migrations, possibly from the same source population, with the more recent descendants gradually diffusing into pre-existing Native American populations.

### Routes of Population Dispersal

Largely on the basis of archaeological data, a classical model for the colonization of the Americas posits that humans entered the region towards the end of the Wisconsin glaciation (∼11,000 y ago) via a mid-continental ice-free corridor between the Cordilleran and Laurentide glaciers [78,79]. According to this model, migration southwards would have followed a pattern with a front of advance at approximately the same latitude across North America.

It is interesting to consider the patterns of genetic structure observed here within the context of the emphasis placed recently on the Pacific coast as an alternative to the inland ice-free corridor route of population dispersal in the Americas [79,85–87]. The late timing of the rapid inland colonization model has been put into some doubt by the discovery of early archaeological sites that predate by thousands of years the most recent deglaciation of North America [88]. In addition, recent geological evidence indicates that ice-free areas west of the Cordilleran ice sheet may have existed as early as ∼14,000 y ago [79], suggesting the possibility of an early coastal migration. Within South America, the coastal colonization model suggests an early southward migration along the western side of the Andes and is consistent with an interpretation that modern speakers of Andean languages may represent descendants of the first occupiers of the region [1]. Recent computer simulations also suggest that a coastal colonization model may more easily explain observed patterns of classical marker and mitochondrial DNA diversity [89].

Several observations from our data are compatible with the proposal of a coastal colonization route. The stronger correlation of genetic diversity with geographic distance when higher coastal mobility is taken into account (Figure 4) supports a possible role for population dispersals along the coast (note, however, that the difference in the tree structure induced by the optimal route in Figure 4 and the tree in Figure 8 suggests that alternative routes might be preferred if more aspects of the genetic data were incorporated into the coastal analysis). Consistent with observations of recent migration paths of certain Amazonian populations [43], we did not find support for migrations along major rivers. Finally, the relative genetic similarity of Andean populations to populations from Mesoamerica (Figure 7) is also compatible with an early Pacific coastal colonization. Under this view, the east-to-west difference in genetic diversity in South America, a pattern also observed with mitochondrial and Y-chromosomal markers [90–92] (including the extremely low diversity in the Ache [93,94] and Surui [94] populations), could reflect an initial colonization of western South America followed by subsampling of western populations to form the eastern populations.

An alternative interpretation of the Mesoamerican and Andean similarity is that this pattern is recent in origin. In this case, the reduced diversity and increased population structure in eastern South America may reflect a deep divergence between western and eastern populations, so that their different levels of differentiation could result from different levels of gene flow and genetic drift in western and eastern South America. The genetic similarity among Andean populations, and their relative similarity to the populations sampled from Mesoamerica, would perhaps then reflect recent gene flow along the coast.

Similar to results seen in some mitochondrial studies [95–97], Central American and South American populations from the Chibchan–Paezan language stock had slightly reduced heterozygosity compared to neighboring populations. Interestingly, the Cabecar and Guaymi populations from lower Central America (Costa Rica and Panama) were robustly placed at the tips of a northwest South American Chibchan–Paezan cluster in the tree of Figure 8. One explanation of this observation is that these populations may be of South American origin, as the ancestral group for the cluster could have been a South American population, most of whose descendants remain in South America. Alternatively, the large cluster containing the Chibchan–Paezan and Equatorial–Tucanoan populations could be the result of a colonization of South America separate from the colonization by the Andean populations—with the founder population possibly speaking a language from which modern Chibchan–Paezan languages have descended [98]. In this view, Guaymi and Cabecar are the only sampled Central American populations descended from the ancestors of this second migration.

### Genetic and Linguistic Differentiation

At a qualitative level, the topology of the tree of Figure 8 shows some correspondence between genetic distance and linguistic stock assignment. High bootstrap values are seen for population clusters corresponding mainly to speakers of Chibchan–Paezan and Equatorial–Tucanoan languages and, to a lesser extent, Central Amerind languages. Although the high bootstrap values support previous qualitative comparisons that have suggested a considerable degree of relationship between genetic and linguistic distances [1], quantitative analyses based on matrix correlation coefficients for genetic and linguistic distances have been somewhat more equivocal [39,99–101]. Indeed, the correlation of genetic and linguistic similarity considering all populations in our dataset is quite small (Table 3). Considering only pairs of populations from within major language stocks, however, the correlation increases. Although several populations that do not group in the neighbor-joining tree with their linguistic neighbors appear most genetically similar to their geographic neighbors, the correlation remains moderate when geographic distance is controlled. The within-stock correlations are in most cases not unusually high when applying permutation tests, but are perhaps suggestive that at the local scale, dissimilarities in languages either play a partial role in producing genetic barriers or otherwise co-occur with factors that impede gene flow. The lack of a more general correlation may be due to such factors as deviations from a tree-like history for genetic evolution or for linguistic evolution, or to uncertainties in the linguistic classification [39].

### Conclusions

In a genomic study of a relatively large number of Native American populations, our work provides support to a variety of hypotheses about fundamental aspects of Native American demographic history. In particular, we find genetic evidence that supports a single main colonization event from Siberia, a coastal colonization route, and a divergence process that may have been facilitated at the local scale partly by differences between languages. As genomic data proliferate, more formal genetic tests of these hypotheses, together with accumulating evidence from fields such as archaeology [78,79,102], geology [103], and linguistics [104–106], will surely result in a more detailed picture of the settlement by and differentiation of indigenous human populations in the American landmass.

## Methods

### Samples.

A total of 436 individuals from 24 Native American populations and one Siberian population were included in this study, in addition to data on 1,048 individuals from 53 worldwide populations represented in the HGDP–CEPH human genome diversity cell line panel [107,108]. Alternate names for the Native American populations, together with sample sizes and approximate geographic coordinates, are given in Table S1.

Populations from the HGDP–CEPH panel were classified into geographic regions as in Rosenberg et al. (2002) [7], and the Tundra Nentsi population from Siberia was classified as East Asian. In analyses subdivided by geographic region within the Americas, we grouped the populations as North American (Chipewyan, Cree, Ojibwa), Central American (Cabecar, Guaymi, Kaqchikel, Maya, Mixe, Mixtec, Pima, Zapotec), western South American (Arhuaco, Aymara, Embera, Huilliche, Inga, Kogi, Quechua, Waunana, Wayuu, Zenu), and eastern South American (Ache, Guarani, Kaingang, Karitiana, Piapoco, Surui, Ticuna [Arara], and Ticuna [Tarapaca]). The populations from Mexico, which except Pima were from the southern part of Mexico, were considered as part of the Central American group. Populations were placed linguistically using the classification of Ruhlen [35]. Although disagreement exists about linguistic classifications in the Americas, there is greater agreement at the level of linguistic stocks and at lower levels in the linguistic classification hierarchy, on which we focus.

### Markers.

Each of the newly sampled individuals was genotyped by the Mammalian Genotyping Service for 751 microsatellites spread across all 22 autosomes. The microsatellite markers were drawn from Marshfield Screening Sets 16 and 54 (http://research.marshfieldclinic.org/genetics/). Considering all individuals, we checked each pair of markers to determine if genotypes at one member of the marker pair were identical to those at the other member of the pair, up to a constant of translation. This procedure identified one pair of duplicated markers—MFD600 and MFD601—and MFD600 was discarded from the analysis.

### Combined dataset with the HGDP–CEPH diversity panel.

Among the 750 remaining microsatellites that were genotyped in the new samples, 693 had previously been genotyped in the HGDP–CEPH diversity panel [7,11,13]. For some of these loci, there was a change in primer length or position between the two studies, or a systematic change occurred in the algorithm by which allele size was determined from raw genotyping products—or both. In cases where the primers changed, allele sizes from the new dataset were adjusted by the appropriate length in order to align its list of allele sizes with the earlier list for the HGDP–CEPH dataset.

To identify systematic changes between datasets, for each locus the allele sizes of one dataset were translated by a constant and the *G* test statistic of independence between allele frequencies and dataset (older HGDP–CEPH dataset versus newly genotyped dataset) was then computed [23]. Considering all possible constants for translation of allele sizes, the one that minimized the *G* statistic was determined. In implementing the *G* test, two groups of comparisons were performed. In the first group of comparisons, the constant of translation was determined by comparing 80 Jewish individuals genotyped simultaneously with the Native Americans to all 255 individuals from Europe and the Middle East in the HGDP–CEPH H1048 dataset [109], excluding Mozabites. The second group of comparisons involved 346 Native American individuals from Central and South America in this newer dataset (all 336 sampled Central and South Americans excluding Ache, and ten additional individuals who were later excluded) and 63 Native American individuals from the Maya, Pima, and Piapoco populations in the older H1048 dataset (the Piapoco population is described as “Colombian” in previous analyses of these data). The constants expected based on the two *G* tests—labeled *S*_1_ for the comparison of the Jewish populations to European and Middle Eastern populations and *S*_2_ for the Native American comparison—were then compared with the constant of translation expected from consideration of three additional sources of information available for the two datasets: the genotypes of a Mammalian Genotyping Service size standard (*S*_3_), a code letter provided by the Mammalian Genotyping Service indicating the nature of the change in primers (*S*_4_), and the locations of the primers themselves in the human genome sequence (*S*_5_).

Among the 693 markers, 687 had the same optimal constant of translation (that is, the constant that minimizes the *G* statistic) in the two different sets of population comparisons (*S*_1_ = *S*_2_). The remaining six markers with different optimal constants of translation in the two *G* tests were compared with the value expected from the locations of the old and new primers in the human genome (*S*_5_). In all six cases, the optimal constant for the comparison of the Jewish and European/Middle Eastern datasets agreed with the value based on the primer locations (*S*_1_ = *S*_5_). As real population differences between datasets are more likely in Native Americans due to the larger overall level of genetic differentiation in the Americas, we used the constant obtained based on the Jewish and European/Middle Eastern comparison (*S*_1_) for allele size calibration.

Of the remaining 687 markers, 638 had an optimal constant of translation that agreed with the value expected based on the code letter provided by the Mammalian Genotyping Service (*S*_1_ = *S*_2_ = *S*_4_). Thus, there were 49 markers for which the code letter was either uninformative or produced a constant of translation that disagreed with *S*_1_ and *S*_2_. For 35 of these markers, the constant of translation based on the size standard (*S*_3_) agreed with *S*_1_ and *S*_2_. For eight of the remaining 14 markers, the constant of translation based on the primer sequences (*S*_5_) agreed with *S*_1_ and *S*_2_. The six markers with disagreements (AAT263P, ATT070, D15S128, D6S1021, D7S817, and TTTAT002Z), having *S*_1_ ≠ *S*_5_, were then discarded. For the remaining 687 markers that were not discarded, 685 had *G* < 48 in both *G* tests, while the other two markers (D14S587 and D15S822) had *G* > 91 in the Jewish versus European/Middle Eastern comparison. These two extreme outliers, which also had the highest *G* values for the Native American comparison, were then excluded (Figure S6).

To further eliminate loci with extreme genotyping errors, we performed Hardy-Weinberg tests [110] within individual populations for the 685 remaining markers. This analysis, performed using *PowerMarker* [111], used only the 44 populations in which all 685 markers were polymorphic. We calculated the fraction of populations with a significant *p*-value (<0.05) for the Hardy-Weinberg test (Figure S7). Two markers (GAAA1C11 and GATA88F08P) were extreme outliers, with more than 43% of populations producing *p* < 0.05. For the remaining markers, the proportion of tests significant at *p* < 0.05 varied from 0 to 35% without any clear outliers, and with most markers having less than 10% of tests significant at *p* < 0.05. Excluding the two Hardy-Weinberg outliers, 683 markers remained. Five additional markers (AGAT120, AGAT142P, D14S592, GATA135G01, and TTTA033) were excluded due to missing data: for each of these markers there was at least one population in which all genotypes were missing. Thus, 678 loci remained for the combined analysis with the HGDP–CEPH panel.

### Final dataset.

After the elimination of problematic markers, ten individuals who had potentially been mislabeled were discarded. Seven of these were admixed individuals from Guatemala who, through a clerical error, had been incorporated in the data cleaning phase of the study as members of the Kaqchikel population. The other three were individuals who, on the basis of elevated allele sharing, were inferred to be siblings, but who were classified as belonging to two different populations (Wayuu and Zenu). The final dataset, combining the HGDP–CEPH data and the new data, contained 1484 individuals and 678 markers, with a missing data rate of 4.0%. Each marker had some data present in all populations, with a minimum 88.7% genotypes per marker and 50.1% genotypes per individual. Of the 1,484 individuals, 1,419 had a missing data rate of less than 10%.

### Detection of relatives.

Identification of pairs of close relatives was performed using identity-by-state allele sharing combined with likelihood inference as implemented in *Relpair* [112,113]. A critical value of 100 was used in the likelihood analysis, and the genotyping error rate was set at 0.008. In each population, *Relpair* was applied using count estimates of allele frequencies in that population. Identification of recommended panels with no first-degree relatives and with no first- or second-degree relatives followed the procedure of Rosenberg [109], except that when an arbitrary decision was required about which individual in a relative pair should be excluded, the individual with more missing data was discarded. Beginning from the 436 newly sampled individuals (termed panel N436), this analysis produced a panel of 379 individuals with no first-degree relatives and a panel of 354 individuals with no first- or second-degree relatives. These panels are termed N379 and N354. Details on the properties of these panels can be found in Tables S13–S26, and plots of allele sharing are shown in Figures S8–S13.

### Geographic computations.

Geographic coordinates for the newly sampled populations are specified in Table S1, and coordinates for the other populations were taken from Rosenberg et al. [13]. For the production of Figure 3B, distances between populations were computed using great circle routes [13], with obligatory waypoints as specified by Ramachandran et al. [11]. Routes to South America required an additional waypoint in Panama at 8.967°N 79.533°W. The computation of Figure 3B excluded the waypoint used by Ramachandran et al. [11] at Prince Rupert, and did not use the Panama waypoint when the origin was placed on a Caribbean island. Geographic distances from East Africa (Figure 3A) were computed using an origin at Addis Ababa [11].

Compared to the waypoint-based geographic distances, effective distances incorporate more detailed information on the effects of landscape components. They are computed as least-cost paths on the basis of a spatial cost map that incorporates these landscape components. For example, a coastal/inland ratio of 1:10 means that it is ten times more costly to go through land than through coastline. The effective distance between two points is computed as the sum of costs (so-called “least-cost distance”) along the least-cost path connecting the points. Because the relative costs of landscape components are somewhat arbitrary, several combinations were tested. We used *PATHMATRIX* [27] to compute least-cost distances based on a “uniform” cost over the continent (that is, when the boundaries of continental landmasses are the only spatial constraint, so that the coastal/inland cost ratio is 1:1), as well as using the following coastal/inland relative cost combinations: 1:2, 1:5, 1:10, 1:20, 1:30, 1:40, 1:50, 1:100, 1:200, 1:300, 1:400, and 1:500. Inverse cost combinations were also tested (2:1, 5:1, 10:1, 20:1, 30:1, 40:1, 50:1, 100:1, 200:1, 300:1, 400:1, 500:1). We also considered scenarios where the cost differed only for the Pacific coast instead of for all coasts, and where it differed not along coasts, but along major rivers.

Least-cost paths were computed on a Lambert azimuthal equal-area projection of the American landmass (central meridian 80°W, reference latitude 10°N) divided into a grid of 100 km^2^ square cells. For each cost scheme, we computed a Pearson correlation between heterozygosity and effective distance from the Bering Strait, as specified by the Anadyr waypoint [11] at 64°N 177°E, and we obtained its significance by using the *t*-distribution transformation [114].

### Heterozygosity and *F*_ST_.

For each population, expected heterozygosity was computed for each locus using an unbiased estimator [115], and the average across loci was taken as the population estimate. Heterozygosity was calculated for pooled collections of populations, and average heterozygosity across populations was obtained within individual geographic regions. Computations of *F*_ST_ were performed using Equation 5.3 of Weir [30], with confidence intervals obtained using 1,000 bootstrap resamples across loci.

### Private alleles.

To assess whether private alleles lie more often at the ends of the allele size range, for a given allele frequency cutoff, *c*, all private alleles with frequency at least *c* in their region of occurrence were obtained. Under the null hypothesis that all alleles are equally likely to be private, the number of private alleles expected to be at one of the two ends of the allele size range was obtained as the sum over the private alleles of 2/*k*_i_, where *k*_i_ denotes the number of distinct alleles worldwide at the locus that produced private allele *i*. A difference from the value expected was evaluated using a chi-square goodness-of-fit test with one degree of freedom. Considering this test for all possible cutoffs *c* below 0.06 (above which only seven private alleles were observed), the most conservative *p*-value was 0.0228, although most values of *c* produced considerably more stringent *p*-values (Figure S14). In depicting allele frequencies at tetranucleotide locus D9S1120 (Figure 10), five of 2,914 observations not differing from the remaining alleles by a multiple of four are grouped with the nearest allele sizes (in one case where the allele was halfway between steps, it was assigned to the larger allele).

### Population structure analysis.

Analysis of population structure was performed using *STRUCTURE* [28,29]. Replicate runs of *STRUCTURE* used a burn-in period of 20,000 iterations followed by 10,000 iterations from which estimates were obtained. All runs were based on the admixture model, in which each individual is assumed to have ancestry in multiple genetic clusters, using the *F* model of correlation in allele frequencies across clusters. Graphs of *STRUCTURE* results were produced using *DISTRUCT* [116].

*Worldwide population structure.* Using the full worldwide data, ten replicate unsupervised *STRUCTURE* runs were performed for each value of the number of clusters *K* from one to 20. For each pair of runs with a given *K*, the symmetric similarity coefficient [117] (SSC) was computed as a measure of the similarity of the outcomes of the two population structure estimates. Using the Greedy algorithm of *CLUMPP* [117], distinct modes among the ten runs with a given *K* were then identified by finding sets of runs so that each pair in a set had *SSC* ≥ 0.9. The average was then taken of the estimated cluster membership coefficients for all runs with the same clustering mode. Of the ten runs, the number of runs that exhibited the mode shown was ten for *K* = 2 and *K* = 4, nine for *K* = 3 (with the tenth run grouping Africans and East Asians rather than Europeans and other Asians), five for *K* = 5 (with the remaining runs subdividing various combinations among Karitiana, Surui, and Ache, rather than separating the two populations from Oceania), and six for *K* = 6 (with the remaining runs subdividing the Native Americans into three clusters rather than separating the two populations from Oceania).

Supervised clustering. Using *STRUCTURE*, individuals from Europe, Sub-Saharan Africa, East Asia (excluding Siberia), and Siberia were forced into separate clusters, and supervised analysis of the Native American data was performed with *K* = 5 clusters. Ten replicates were performed, each of which yielded the same clustering mode, and the average membership coefficients across these replicates are displayed in Figure 6.

*Native Americans* **.** Using the Native Americans only, 100 replicate unsupervised *STRUCTURE* runs were performed for each value of *K* from one to 15 clusters. The settings for the runs were the same as in the worldwide analysis, and modes were identified in a similar manner. For *K* ≤ 9, average membership coefficients for the most frequently observed mode at each *K* are displayed in Figure 7. For each value of *K*, the figure presents the average membership estimates across all replicates that produced the most frequently occurring solution. Because of the high level of multimodality for *K* ≥ 3, no single mode provides a complete representation of the *STRUCTURE* results with a given *K*. Using *CLUMPP* [117], we identified all modes appearing at least 12 times in 100 replicates, using the *SSC* ≥ 0.9 criterion. Computations of *SSC* were based on the best alignment of the 100 replicate analyses obtained using the LargeKGreedy algorithm of *CLUMPP* with 1,000 (2 ≤ *K* ≤ 11) or 200 (12 ≤ *K* ≤ 15) random input sequences.

For 2 ≤ *K* ≤ 9, using the criterion SSC ≥ 0.9, the relation “in the same mode” had the property of being transitive, so that if runs (*R*_1_,*R*_2_) were in the same mode and runs (*R*_2_,*R*_3_) were in the same mode, then runs (*R*_1_,*R*_3_) were also in the same mode. For *K* ≥ 10, with the criterion *SSC* ≥ 0.9, “in the same mode” was not always transitive. While other cutoffs *c* could sometimes be identified so that “in the same mode” was transitive when the criterion *SSC* > *c* was applied, for *K* ≥ 10 there was no clear plateau in the cumulative probability distribution of *SSC* values across pairs of runs (Figure S15). Such plateaus, which are observed for 2 ≤ *K* ≤ 9, represent a large gap between *SSC* values for pairs of runs truly in the same mode (high *SSC*) and pairs of runs not in the same mode (lower *SSC*). The fact that for *K* ≥ 10 the probability is high that a randomly chosen pair of runs has *SSC* < 0.9 is also indicative of considerable multimodality across replicates.

Considering the modes with successive numbers of clusters, we identified all sets of modes with *K*+1 clusters that “refined” modes with *K* clusters. A mode with *K*+1 clusters is a refinement of a mode with *K* clusters if the mode with *K*+1 clusters consists of *K*−1 of the clusters in the *K*-cluster mode together with two clusters obtained by splitting the *K*th cluster into two subgroups. More generally, a mode with *K* > *L* clusters refines a mode with *L* clusters if each cluster in the *K*-cluster mode is either the same as or a subdivision of a cluster in the *L*-cluster mode. As an example, in Figure 7, the mode depicted for *K* = 7 is a refinement of all modes depicted for smaller values of *K*.

For the Native American data, we performed a separate analysis using *TESS* [118,119], a genetic clustering program that estimates a preferred value of the number of clusters *K* less than or equal to a prespecified maximum value *K*_max_. If the estimated *K* equals *K*_max_, then the choice of *K*_max_ is insufficiently large. Using the *TESS* admixture model with burn-in period of length 10,000 followed by 20,000 iterations from which estimates were obtained, we performed 200 runs of *TESS* with *K*_max_ = 10, 20 each for ten values of a spatial autocorrelation parameter *Ψ* at intervals of 0.2 from 0.2 to 2. Of these 200 replicates, 183 supported an inference of *K* = 6, 7, 8, or 9, and only one supported an inference of *K* = 10. This suggests that the most important components of population structure are apparent with *K* < 10.

### Population tree.

An unrooted neighbor-joining [120] population tree was constructed for the Native American and Siberian populations based on the *D*_a_ distance of Nei et al. [36], which was found to perform comparatively well in estimation of population trees from microsatellite allele frequency data [121]. To visualize the tree, the root was placed between the Siberian and Native American populations. Confidence values were obtained from 1000 bootstrap resamples across loci. The computation of bootstrap distances was performed using *PowerMarker* [111], and the consensus tree was obtained and plotted using *MEGA3* [122]. For comparison, trees based on Reynolds [123] and chord distances [124] were obtained analogously. Genetic distance matrices based on the Nei, Reynolds, and chord distances are shown in Tables S27–S29.

### Correlations of genes and languages.

We used a discretized scale to measure linguistic distance [125,126]. Two populations from different language stocks or “groups” (Table S2) were scored as having distance 4, and within stocks, two populations had distance of 1, 2, or 3 depending on the level at which their languages diverged (Table S3). For some computations, we devised discretized measures of linguistic distance between stocks on the basis of shared and unshared etymologies tabulated in Table C.1 of Greenberg [34] (Tables S4 and S5). Using these etymologies, we computed the Dice (simple matching) and Jaccard indices of dissimilarity between stocks [127] (Tables S6 and S7), which we then converted into discretized between-stock distances (Tables S8 and S9).

For comparison with linguistic distances, *D*_a_ genetic distances were used (Table S27), and the Mantel correlation coefficients [38] between pairs of distance matrices (among genetic, geographic, and linguistic) were obtained, with significance assessed using 10,000 permutations of rows and columns. Waypoint-based distances (Table S30) were used for the geographic computations. For computations within linguistic stocks, the correlation and significance level were computed as in tests involving the full matrix, except that all entries between language stocks were omitted from the evaluation of the correlation coefficient. Partial correlations of genetic and linguistic distance controlling for geographic distance were also obtained [128], with geographic distance calculated using the waypoint approach as above.

### Analysis excluding relatives.

As the inclusion of relatives has the potential to influence various types of population-genetic analysis, we compared some of our results based on the full collection of 1,484 individuals to results based on 1,306 individuals—the H952 set from the HGDP–CEPH diversity panel [109] together with the N354 set from the newly genotyped individuals. The inclusion of relatives does not lead to a bias in allele frequency estimates (that is, *E*[**p̂*_i_*] still equals *p*_i_), but it does inflate *Var*[**p̂**_*i*_]. The estimator *Ĥ* of heterozygosity is 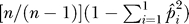 , where *n* is sample size, the sum proceeds over alleles, and **p̂**_*i*_ is the estimated frequency of allele *i*. Expanding the expression for the expectation *E*[*Ĥ*], it can be observed that the coefficient for the *Var*[**p̂**_*i*_] term is negative. Thus, inclusion of relatives is expected to reduce the estimate of heterozygosity through an increase in *Var*[**p̂**_*i*_].

The population heterozygosities based on the full and reduced datasets are plotted in Figure S16. The correlation coefficient between population heterozygosities based on the reduced and full datasets was 0.997; as expected, however, heterozygosity was systematically higher in the reduced set (mean difference of 0.0033 across populations; *p*< 0.001, Wilcoxon signed rank test). Given the greater proportion of individuals excluded when relatives were removed from N436 (18.8%) compared to H1048 (9.2%), the difference in heterozygosities between full and reduced datasets is greater in the 25 newly sampled populations (mean difference of 0.0052; *p* < 0.001) compared to the 53 HGDP–CEPH populations (mean difference of 0.0024; *p* < 0.001).

Despite the detectable effect of the removal of relatives on heterozygosity, the systematic nature of this small effect was such that very little difference was observed on the relationship of heterozygosity with distance from the Bering Strait (Figure S17). A number of other analyses, including the analyses of linguistic correlations and numbers of private and distinct alleles, also produced nearly identical inferences when relatives were excluded (Figures S18–S20 and Tables S31–S33), two exceptions being a noticeable decrease in population differentiation (Table S31) and a shift in the position of several populations in the neighbor-joining tree (Figure S19). Via the connection between heterozygosity and differentiation [11,31–33], the decrease in differentiation is a consequence of the increase in heterozygosity upon exclusion of relatives. In the case of the tree, despite a Mantel correlation of 0.99 between genetic distance matrices including and excluding relatives (Tables S27 and S33), the Cree, Huilliche, Maya, Ojibwa, Wayuu and Zenu populations shifted positions slightly, and the Kaqchikel population moved nearer to its geographic neighbors. Although the population groupings were generally quite similar, several bootstrap values decreased, magnifying the effect of the slight decrease in population differentiation.

## Supporting Information

Dataset S1 Gzipped File with the Genotypes for 1,484 Individuals at 678 Loci (2.1 MB GZ).

Figure S1 Heterozygosity and Least-Cost Paths in a Coastal Migration Scenario, with Ache, Guarani, and Kaingang Excluded from the Analysis The figure design follows that of Figure 4, with part B based on the scenario with 1:10 coastal/inland cost ratio.
(635 KB PDF)

Figure S2 Heterozygosity and Least-Cost Paths in a Pacific Coastal Migration Scenario The figure design follows that of Figure 4, with part B based on the scenario with 1:2 Pacific coastal/inland cost ratio.
(644 KB PDF)

Figure S3 Heterozygosity and Least-Cost Paths in Five Migration Scenarios The figure design follows that of Figure 4A.
(1.1 MB PDF)

Figure S4 Neighbor-Joining Tree of Native American Populations Based on Reynolds Genetic Distance The figure design follows that of Figure 8.
(481 KB PDF)

Figure S5 Neighbor-Joining Tree of Native American Populations Based on Chord Genetic Distance The figure design follows that of Figure 8.
(462 KB PDF)

Figure S6 *G* Test Statistics for Agreement of Calibrated Allele Sizes in Two Population Comparisons (1.3 MB PDF)

Figure S7 Frequency Distribution Across Markers of the Fraction of Populations Whose *p*-Values for a Test of Hardy-Weinberg Equilibrium Were Below 0.05 (538 KB PDF)

Figure S8 Allele Sharing for Pairs of Individuals from Different Populations The four points farthest to the left all involve pairs in which one individual is Kogi 2463 and the other is from the Arhuaco population.
(412 KB PDF)

Figure S9 Allele Sharing for Within-Population Pairs of Individuals from Each of Five Populations: Ache, Arhuaco, Aymara, Cabecar, and Chipewyan Parent/offspring (PO), full sib (FS), and second-degree (2nd) relative pairs are indicated on the graphs.
(1.0 MB PDF)

Figure S10 Allele Sharing for Within-Population Pairs of Individuals from Each of Five Populations: Cree, Embera, Guarani, Guaymi, and Huilliche Parent/offspring (PO), full sib (FS), and second-degree (2nd) relative pairs are indicated on the graphs.
(959 KB PDF)

Figure S11 Allele Sharing for Within-Population Pairs of Individuals from Each of Five Populations: Inga, Kaingang, Kaqchikel, Kogi, and Mixe Parent/offspring (PO), full sib (FS), and second-degree (2nd) relative pairs are indicated on the graphs.
(969 KB PDF)

Figure S12 Allele Sharing for Within-Population Pairs of Individuals from Each of Five Populations: Mixtec, Ojibwa, Quechua, Ticuna (Arara), and Ticuna (Tarapaca) Parent/offspring (PO), full sib (FS), and second-degree (2nd) relative pairs are indicated on the graphs.
(1.0 MB PDF)

Figure S13 Allele Sharing for Within-Population Pairs of Individuals from Each of Five Populations: Tundra Nentsi, Waunana, Wayuu, Zapotec, and Zenu Parent/offspring (PO), full sib (FS), and second-degree (2nd) relative pairs are indicated on the graphs.
(1.0 MB PDF)

Figure S14 *p*-Value for the Goodness-of-Fit Test of the Hypothesis that Private Allele Sizes Match the Expectation, as a Function of the Minimum Frequency of Private Alleles Considered (213 KB PDF)

Figure S15 Cumulative Probability Distribution of the 4,950 Pairwise Symmetric Similarity Coefficients among 100 Runs of *STRUCTURE* with Given Values of *K*
The cutoff *SSC* = 0.9 is marked by a vertical line.
(173 KB PDF)

Figure S16 Heterozygosity of Individual Populations in the Reduced Dataset of 1,306 Individuals versus Heterozygosity in the Full Dataset of 1,484 Individuals The 25 newly examined populations are marked in red, and the previously genotyped populations are marked in yellow.
(173 KB PDF)

Figure S17 Heterozygosity in Relation to Geography for the Reduced Dataset of 1,306 Individuals The figure design follows that of Figure 3. Denoting heterozygosity by *H* and geographic distance in thousands of kilometers by *D*, the regression line for the graph is *H* = 0.7668 − 0.00624*D*, with correlation coefficient −0.867. Across the Americas, the correlation between heterozygosity and distance from the Bering Strait ranges from −0.457 to 0.573, and color bins are set to equalize the number of points drawn in the four colors. From darkest to lightest, the four colors represent points with correlations in (−0.457, −0.444), (−0.444, −0.328), (−0.328, 0.498), and (0.498, 0.573), respectively. The most strongly negative correlation occurs at 58.2117°N 95.2°W. Of 1,246 points plotted, 44 change colors compared to Figure 3.
(74 KB PDF)

Figure S18 The Mean and Standard Error Across 678 Loci of the Number of Distinct Alleles as a Function of the Number of Sampled Chromosomes, for the Reduced Dataset of 1,306 Individuals The figure design follows that of Figure 2, with the results based on 1,306 individuals superimposed using thin lines on the results obtained with all 1,484 individuals.
(530 KB PDF)

Figure S19 Neighbor-Joining Tree of Native American Populations Based on Nei Genetic Distance, Using the Reduced Dataset of 1,306 Individuals The figure design follows that of Figure 8.
(472 KB PDF)

Figure S20 The Mean and Standard Error Across 678 Loci of the Number of Private Alleles as a Function of the Number of Sampled Chromosomes, for the Reduced Dataset of 1,306 Individuals The figure design follows that of Figure 9, with the results based on 1,306 individuals superimposed using thin lines on the results obtained with all 1,484 individuals.
(514 KB PDF)

Table S1 Native American Populations Included in This Study, and the Coordinates Used for Their Sampling Locations (18 KB PDF)

Table S2 Languages of the Native American Populations Included in This Study, Classified According to Greenberg [34] and Ruhlen [35] (16 KB PDF)

Table S3 Discretized Matrix of Linguistic Distances for Pairs of Populations, with All Stocks Treated as Equidistant (42 KB PDF)

Table S4 Number of Etymologies Shared between Language Stocks, Among the 281 Etymologies Examined by Greenberg [34] (14 KB PDF)

Table S5 Number of Etymologies Unshared between Language Stocks, Among the 281 Etymologies Examined by Greenberg [34] (14 KB PDF)

Table S6 Matrix of Linguistic Distances between Stocks, Based on the Dice Index (15 KB PDF)

Table S7 Matrix of Linguistic Distances between Stocks, Based on the Jaccard Index (15 KB PDF)

Table S8 Discretized Matrix of Linguistic Distances between Stocks, Based on the Dice Index (14 KB PDF)

Table S9 Discretized Matrix of Linguistic Distances between Stocks, Based on the Jaccard Index (14 KB PDF)

Table S10 Discretized Matrix of Linguistic Distances for Pairs of Populations, with Stock Distances Based on the Dice Index (45 KB PDF)

Table S11 Discretized Matrix of Linguistic Distances for Pairs of Populations, with Stock Distances Based on the Jaccard Index (46 KB PDF)

Table S12 Correlation of Heterozygosity with Distance from the Bering Strait When Excluding Populations with the Highest Level of European Admixture (12 KB PDF)

Table S13 Inferred Relative Pairs within Populations (Part I) (14 KB PDF)

Table S14 Inferred Relative Pairs within Populations (Part II) (14 KB PDF)

Table S15 Inferred Relative Pairs within Populations (Part III) (14 KB PDF)

Table S16 Inferred Relative Pairs within Populations (Part IV) (14 KB PDF)

Table S17 Inferred Relative Pairs within Populations (Part V) (14 KB PDF)

Table S18 Number of Inferred Relative Pairs within Populations (13 KB PDF)

Table S19 The 38 Inferred Parent/Offspring Pairs in N436 (15 KB PDF)

Table S20 The 47 Inferred Full Sib Pairs in N436 (15 KB PDF)

Table S21 The 31 Inferred Within-Population Second-Degree Relative Pairs in N436 (15 KB PDF)

Table S22 The Five Inferred Parent/Parent/Offspring Trios in N436 (12 KB PDF)

Table S23 Numbers of Individuals Excluded from N436 in N379 and N354 (14 KB PDF)

Table S24 The 57 Individuals Included in N436 but Not in N379 (18 KB PDF)

Table S25 The 82 Individuals Included in N436 but Not in N354 (18 KB PDF)

Table S26 The Missing Data Rate in N436, N379, and N354 (13 KB PDF)

Table S27 Nei Genetic Distance Matrix for Native American Populations (21 KB PDF)

Table S28 Reynolds Genetic Distance Matrix for Native American Populations (21 KB PDF)

Table S29 Chord Genetic Distance Matrix for Native American Populations (22 KB PDF)

Table S30 Waypoint Geographic Distance Matrix for Native American Populations, in Kilometers (20 KB PDF)

Table S31 Heterozygosity and F_ST_ (×100) for Various Geographic Regions, Based on a Reduced Dataset of 1,306 Individuals (17 KB PDF)

Table S32 Correlation of Genetic and Linguistic Distances, Based on a Reduced Set of 1,306 Individuals (12 KB PDF)

Table S33 Nei Genetic Distance Matrix for Native American Populations, Based on a Reduced Set of 1,306 Individuals (21 KB PDF)

Text S1 Readme Text Accompanying the File with the Genotypes for 1,484 Individuals at 678 Loci (1 KB TXT)

## Abbreviations

CEPH: Centre d'Etude du Polymorphisme Humain
HGDP: Human Genome Diversity Project
