# Supplementary material for: Genetic Variation and Population Structure in Native Americans
Source: PLoS Genet. 2007 Nov 23;3(11):e185. doi: 10.1371/journal.pgen.0030185 (PMC2082466; doi:10.1371/journal.pgen.0030185)
Supplement: Supplementary file 1 [file pgen.0030185.sg001.pdf]

A

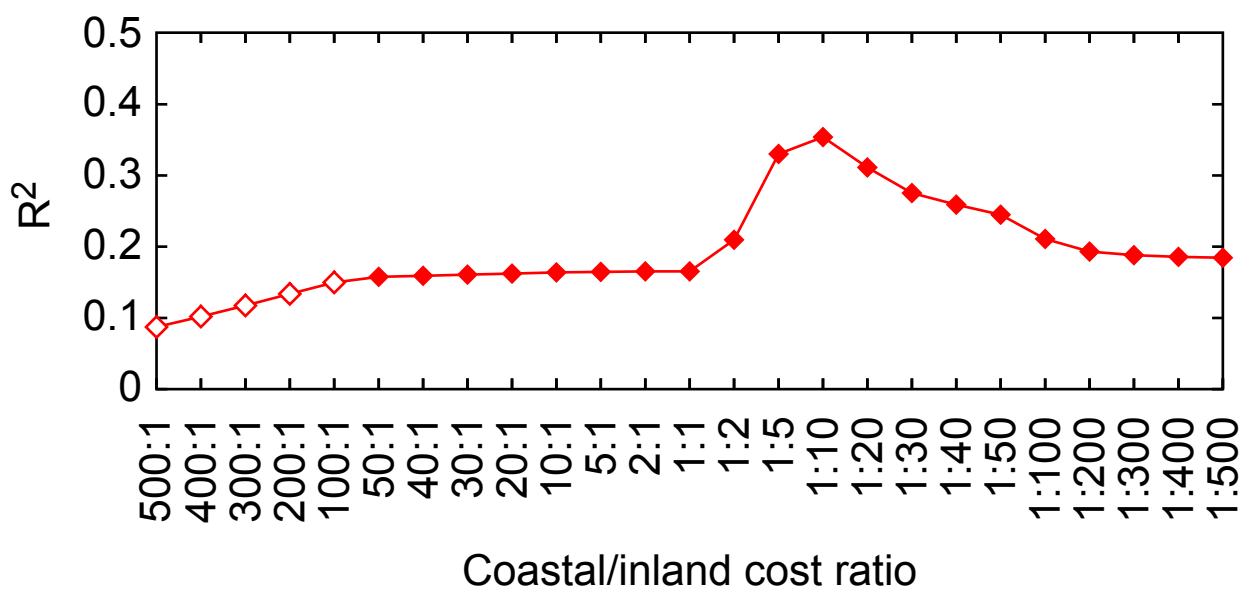

0.5
0.4
0.3
0.2
0.1
0
R^2
500:1
400:1
300:1
200:1
100:1
50:1
40:1
30:1
20:1
10:1
5:1
2:1
1:1
1:2
1:5
1:10
1:20
1:30
1:40
1:50
1:100
1:200
1:300
1:400
1:500
Coastal/inland cost ratio

B

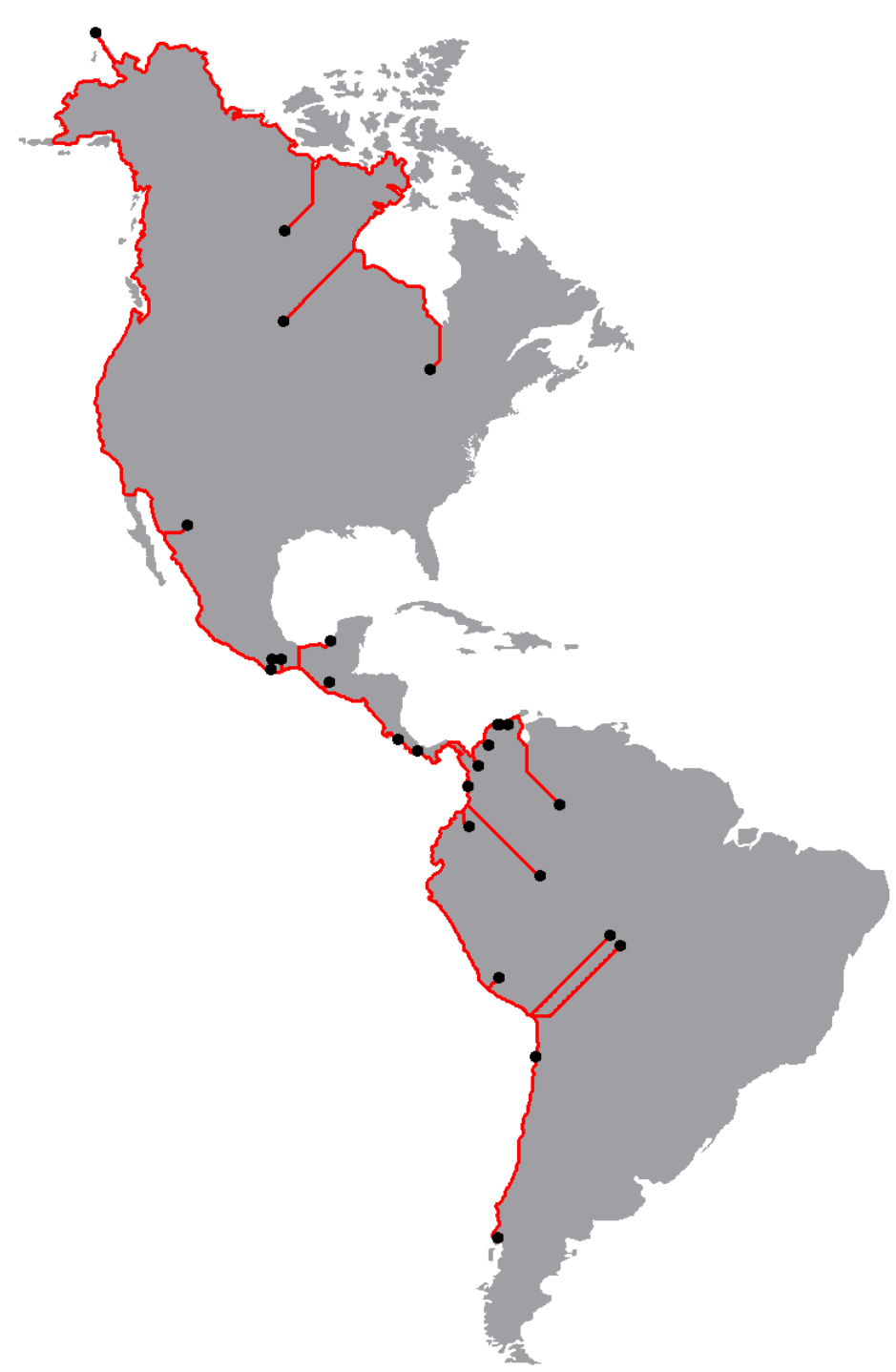

Figure S1
