# Supplementary material for: Genetic Variation and Population Structure in Native Americans
Source: PLoS Genet. 2007 Nov 23;3(11):e185. doi: 10.1371/journal.pgen.0030185 (PMC2082466; doi:10.1371/journal.pgen.0030185)
Supplement: Supplementary file 3 [file pgen.0030185.sg003.pdf]

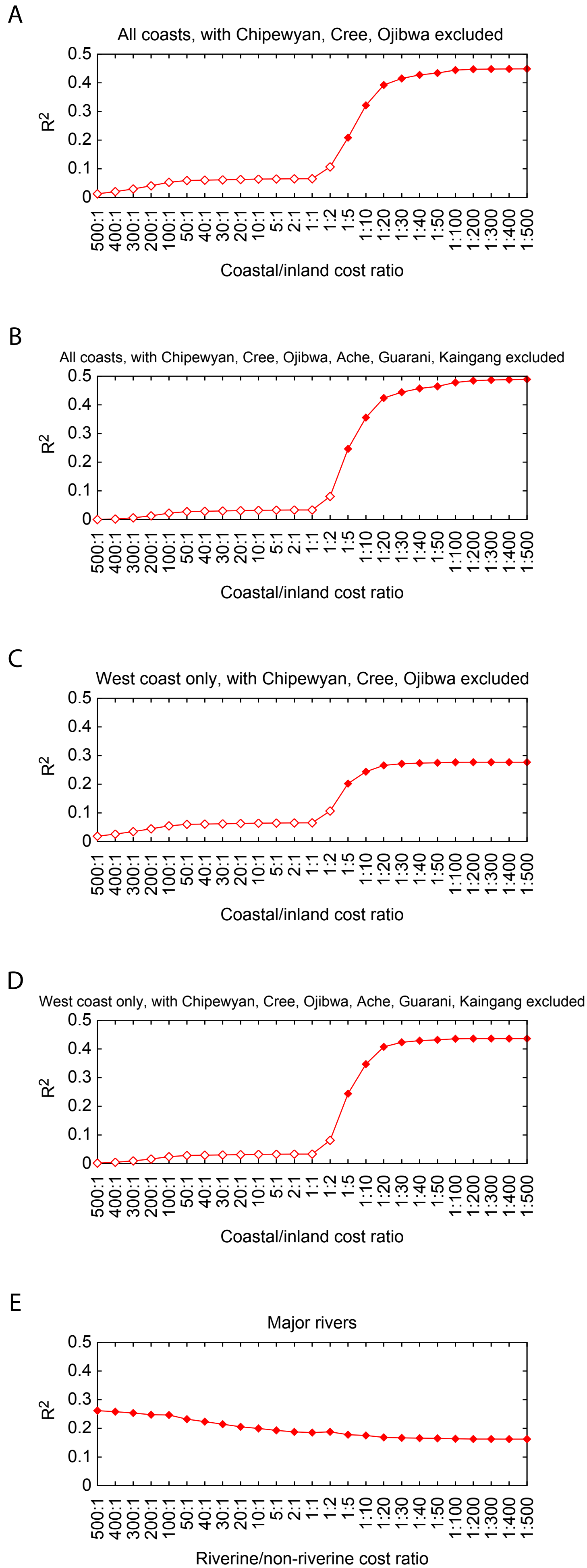
A
All coasts, with Chipewyan, Cree, Ojibwa excluded
R2
0.5
0.4
0.3
0.2
0.1
0
500:1
400:1
300:1
200:1
100:1
50:1
40:1
30:1
20:1
10:1
5:1
2:1
1:1
1:2
1:5
1:10
1:20
1:30
1:40
1:50
1:100
1:200
1:300
1:400
1:500
Coastal/inland cost ratio
B
All coasts, with Chipewyan, Cree, Ojibwa, Ache, Guarani, Kaingang excluded
R2
Coastal/inland cost ratio
C
West coast only, with Chipewyan, Cree, Ojibwa excluded
R2
Coastal/inland cost ratio
D
West coast only, with Chipewyan, Cree, Ojibwa, Ache, Guarani, Kaingang excluded
R2
Coastal/inland cost ratio
E
Major rivers
R2
Riverine/non-riverine cost ratio

Figure S3
