# Supplementary material for: Genetic Variation and Population Structure in Native Americans
Source: PLoS Genet. 2007 Nov 23;3(11):e185. doi: 10.1371/journal.pgen.0030185 (PMC2082466; doi:10.1371/journal.pgen.0030185)
Supplement: Supplementary file 4 [file pgen.0030185.sg004.pdf]

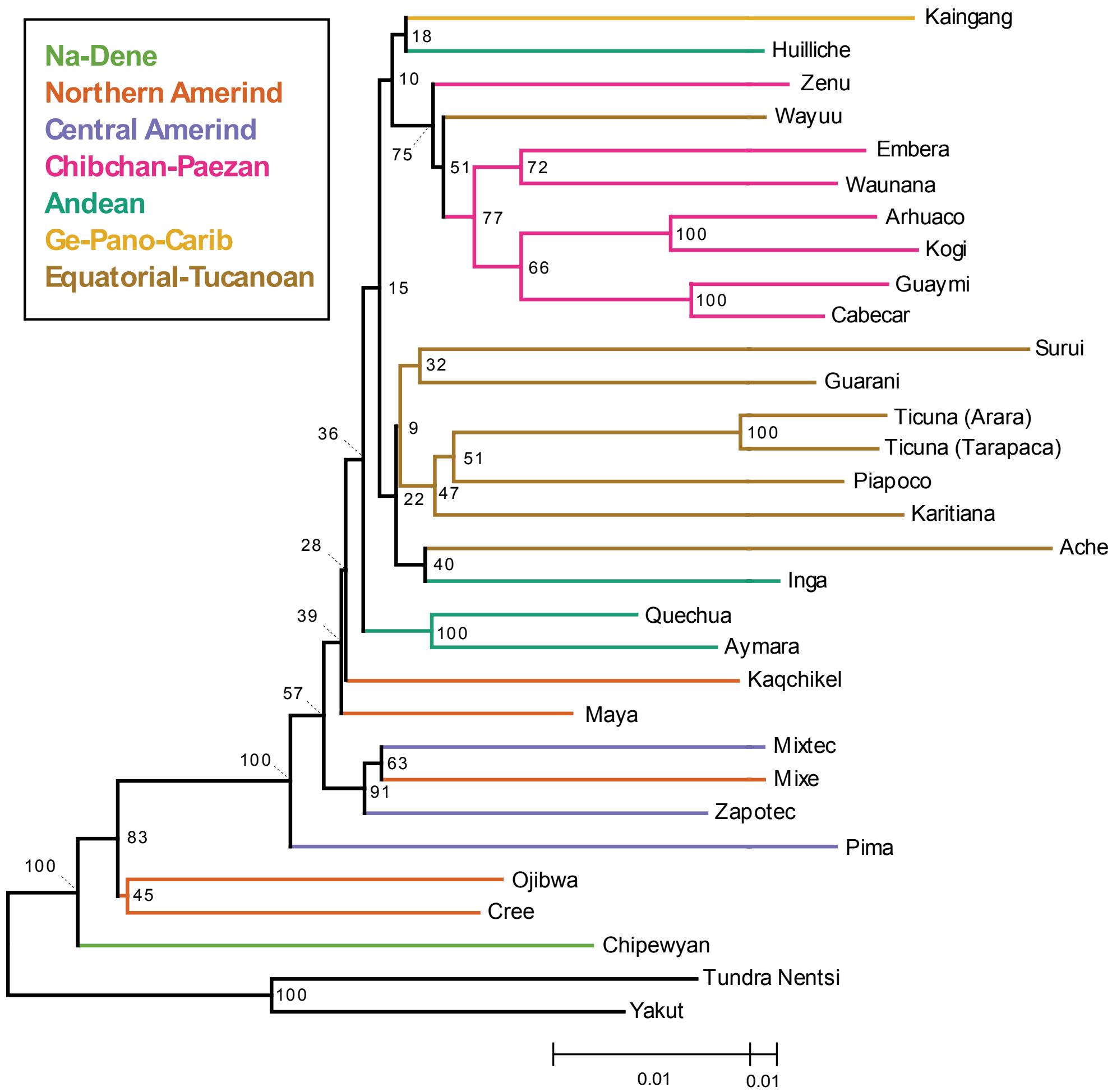
Na-Dene
Northern Amerind
Central Amerind
Chibchan-Paezan
Andean
Ge-Pano-Carib
Equatorial-Tucanoan
Kaingang
Huilliche
Zenu
Wayuu
Embera
Waunana
Arhuaco
Kogi
Guaymi
Cabecar
Surui
Guarani
Ticuna (Arara)
Ticuna (Tarapaca)
Piapoco
Karitiana
Ache
Inga
Quechua
Aymara
Kaqchikel
Maya
Mixtec
Mixe
Zapotec
Pima
Ojibwa
Cree
Chipewyan
Tundra Nentsi
Yakut
18
10
75
51
72
77
100
66
100
15
32
9
100
51
36
22
47
28
40
39
100
57
63
100
91
83
100
45
100
0.01
0.01

Figure S4
