# Supplementary material for: Genetic Variation and Population Structure in Native Americans
Source: PLoS Genet. 2007 Nov 23;3(11):e185. doi: 10.1371/journal.pgen.0030185 (PMC2082466; doi:10.1371/journal.pgen.0030185)
Supplement: Supplementary file 5 [file pgen.0030185.sg005.pdf]

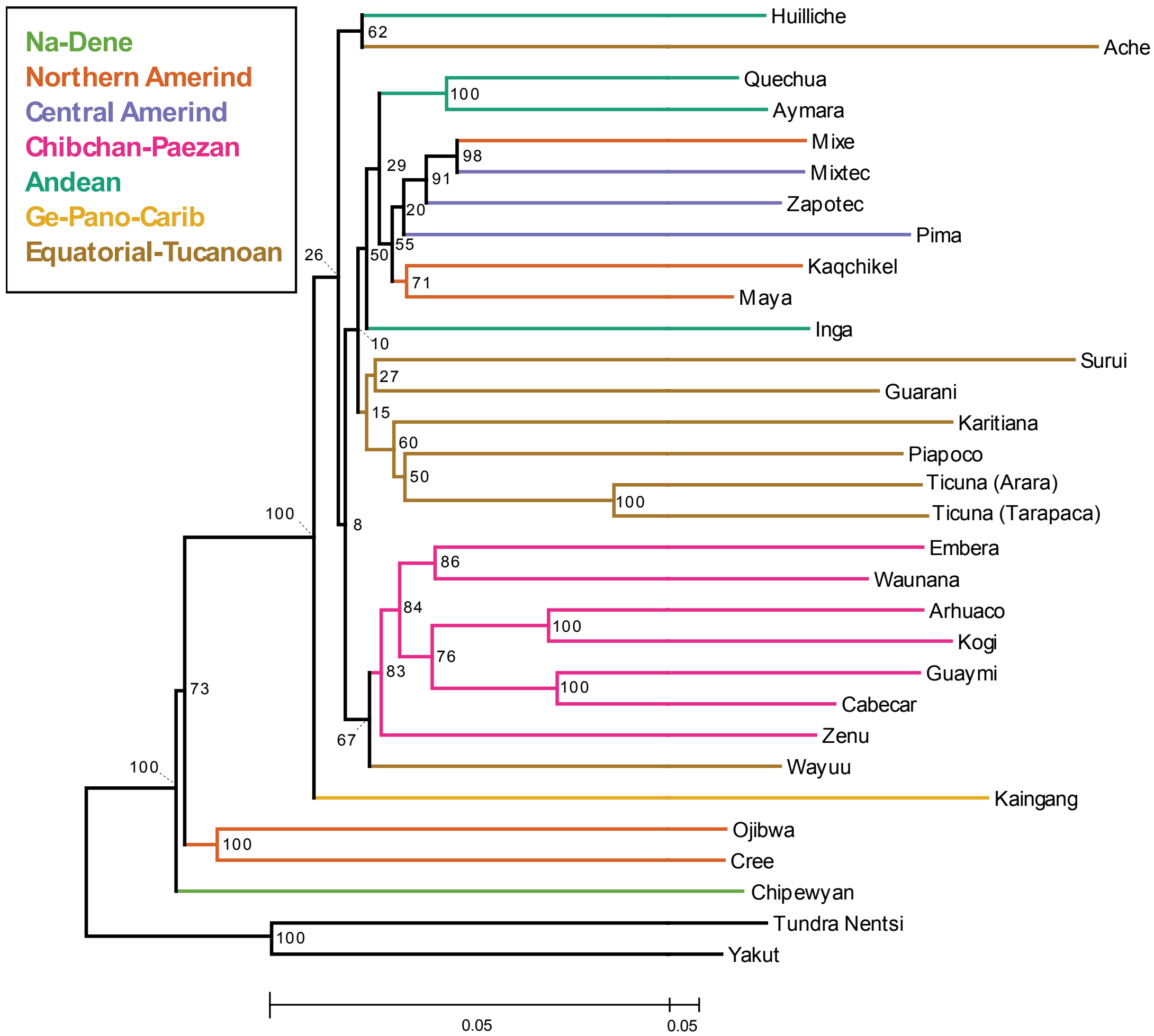
Na-Dene
Northern Amerind
Central Amerind
Chibchan-Paezan
Andean
Ge-Pano-Carib
Equatorial-Tucanoan
Huilliche
Ache
Quechua
Aymara
Mixe
Mixtec
Zapotec
Pima
Kaqchikel
Maya
Inga
Surui
Guarani
Karitiana
Piapoco
Ticuna (Arara)
Ticuna (Tarapaca)
Embera
Waunana
Arhuaco
Kogi
Guaymi
Cabecar
Zenu
Wayuu
Kaingang
Ojibwa
Cree
Chipewyan
Tundra Nentsi
Yakut
0.05
0.05

Figure S5
