# Supplementary material for: Genetic Variation and Population Structure in Native Americans
Source: PLoS Genet. 2007 Nov 23;3(11):e185. doi: 10.1371/journal.pgen.0030185 (PMC2082466; doi:10.1371/journal.pgen.0030185)
Supplement: Supplementary file 6 [file pgen.0030185.sg006.pdf]

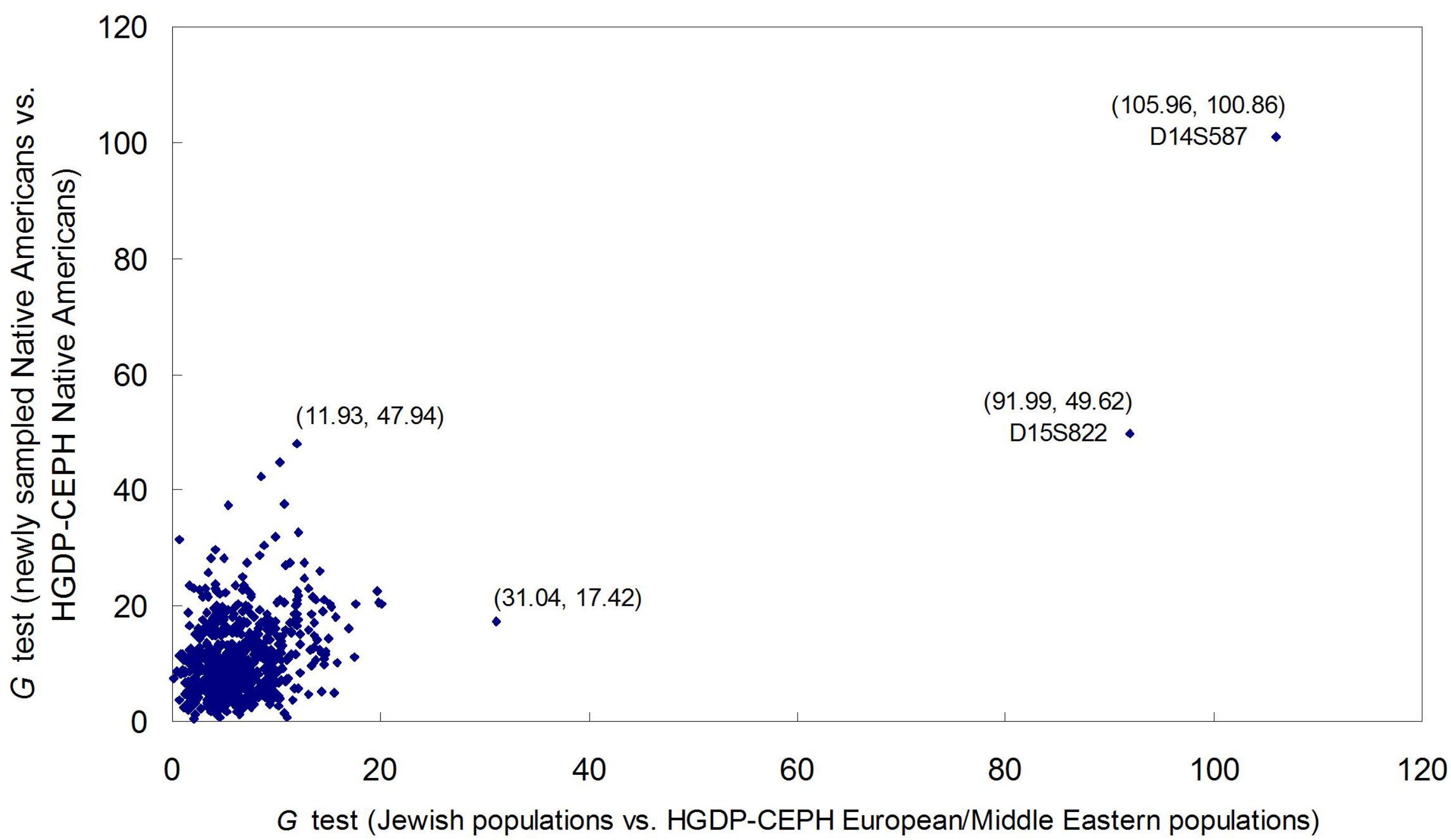

G test (newly sampled Native Americans vs. HGDP-CEPH Native Americans)
120
100
80
60
40
20
0
(105.96, 100.86)
D14S587
(91.99, 49.62)
D15S822
(11.93, 47.94)
(31.04, 17.42)
0
20
40
60
80
100
120
G test (Jewish populations vs. HGDP-CEPH European/Middle Eastern populations)

Figure S6
