# Supplementary material for: Genetic Variation and Population Structure in Native Americans
Source: PLoS Genet. 2007 Nov 23;3(11):e185. doi: 10.1371/journal.pgen.0030185 (PMC2082466; doi:10.1371/journal.pgen.0030185)
Supplement: Supplementary file 7 [file pgen.0030185.sg007.pdf]

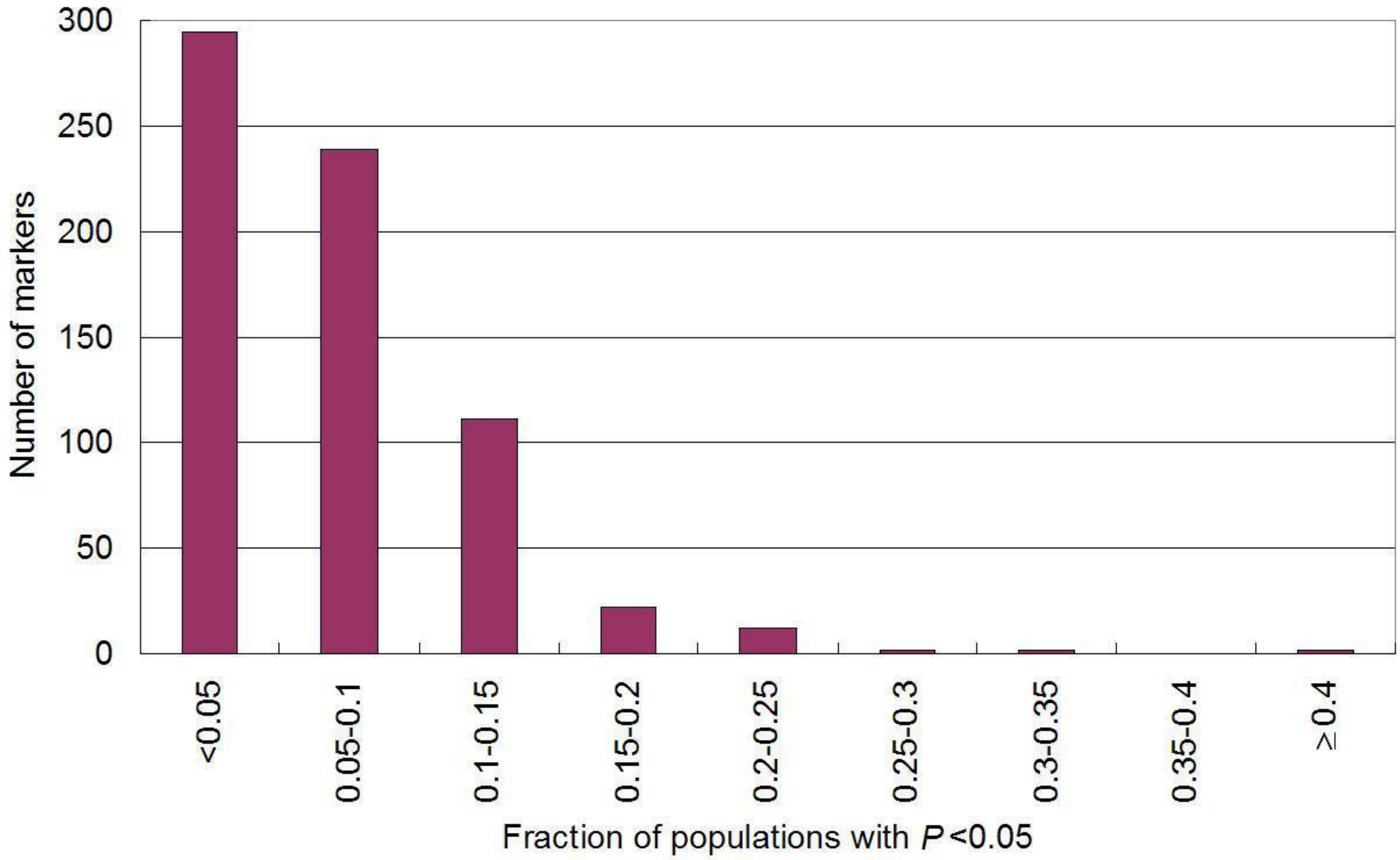
300
250
200
150
100
50
0
Number of markers
<0.05
0.05-0.1
0.1-0.15
0.15-0.2
0.2-0.25
0.25-0.3
0.3-0.35
0.35-0.4
≥0.4
Fraction of populations with P<0.05

Figure S7
