# Supplementary material for: Genetic Variation and Population Structure in Native Americans
Source: PLoS Genet. 2007 Nov 23;3(11):e185. doi: 10.1371/journal.pgen.0030185 (PMC2082466; doi:10.1371/journal.pgen.0030185)
Supplement: Supplementary file 9 [file pgen.0030185.sg009.pdf]

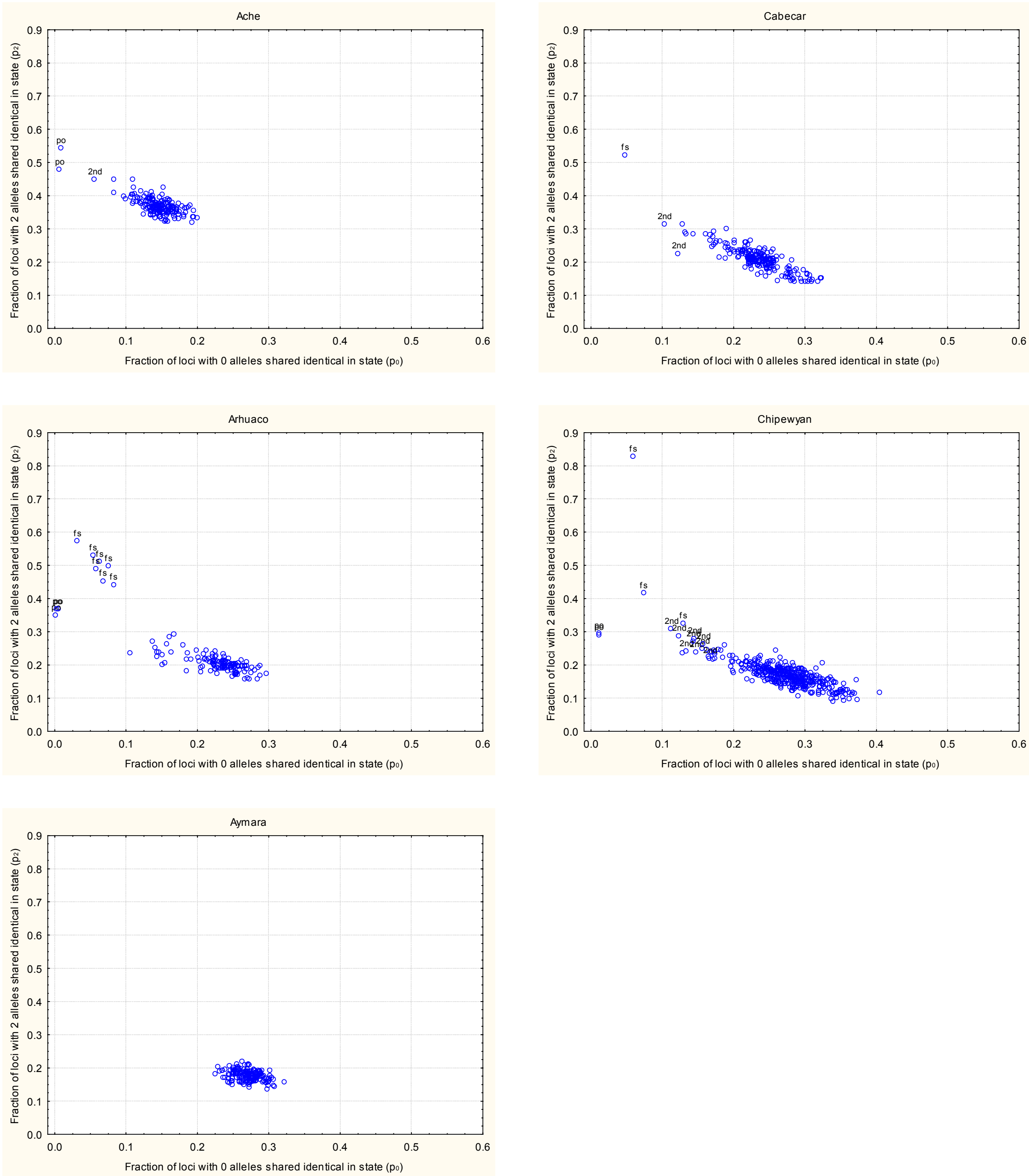

Ache
Cabecar
Arhuaco
Chipewyan
Aymara
Fraction of loci with 2 alleles shared identical in state (p_2)
Fraction of loci with 0 alleles shared identical in state (p_0)
po
2nd
fs

Figure S9
