# Supplementary material for: Genetic Variation and Population Structure in Native Americans
Source: PLoS Genet. 2007 Nov 23;3(11):e185. doi: 10.1371/journal.pgen.0030185 (PMC2082466; doi:10.1371/journal.pgen.0030185)
Supplement: Supplementary file 10 [file pgen.0030185.sg010.pdf]

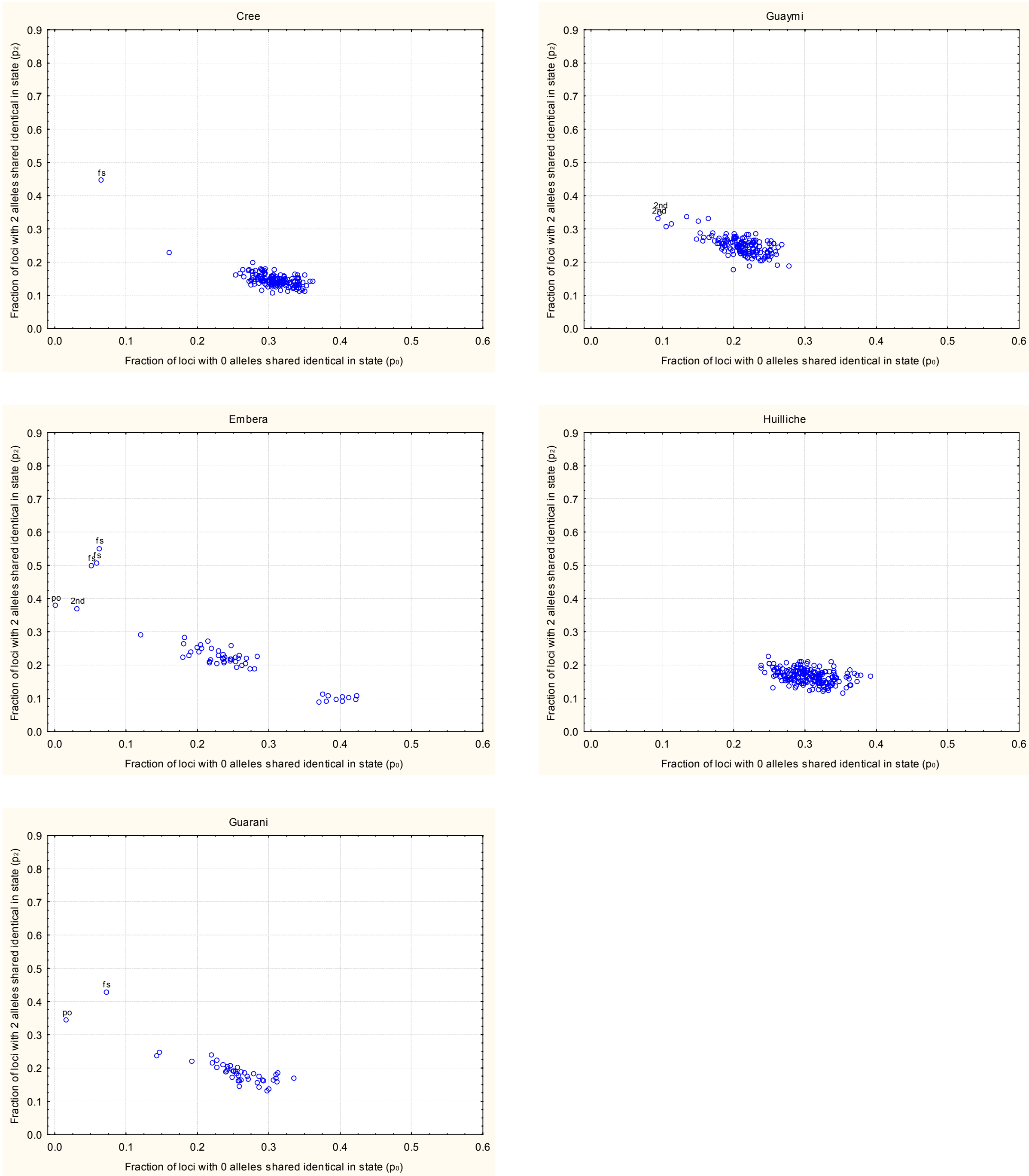

Cree
Guaymi
Embera
Huilliche
Guarani
Fraction of loci with 2 alleles shared identical in state (p_2)
Fraction of loci with 0 alleles shared identical in state (p_0)
0.0
0.1
0.2
0.3
0.4
0.5
0.6
0.7
0.8
0.9
fs
2nd
po

Figure S10
