# Supplementary material for: Genetic Variation and Population Structure in Native Americans
Source: PLoS Genet. 2007 Nov 23;3(11):e185. doi: 10.1371/journal.pgen.0030185 (PMC2082466; doi:10.1371/journal.pgen.0030185)
Supplement: Supplementary file 11 [file pgen.0030185.sg011.pdf]

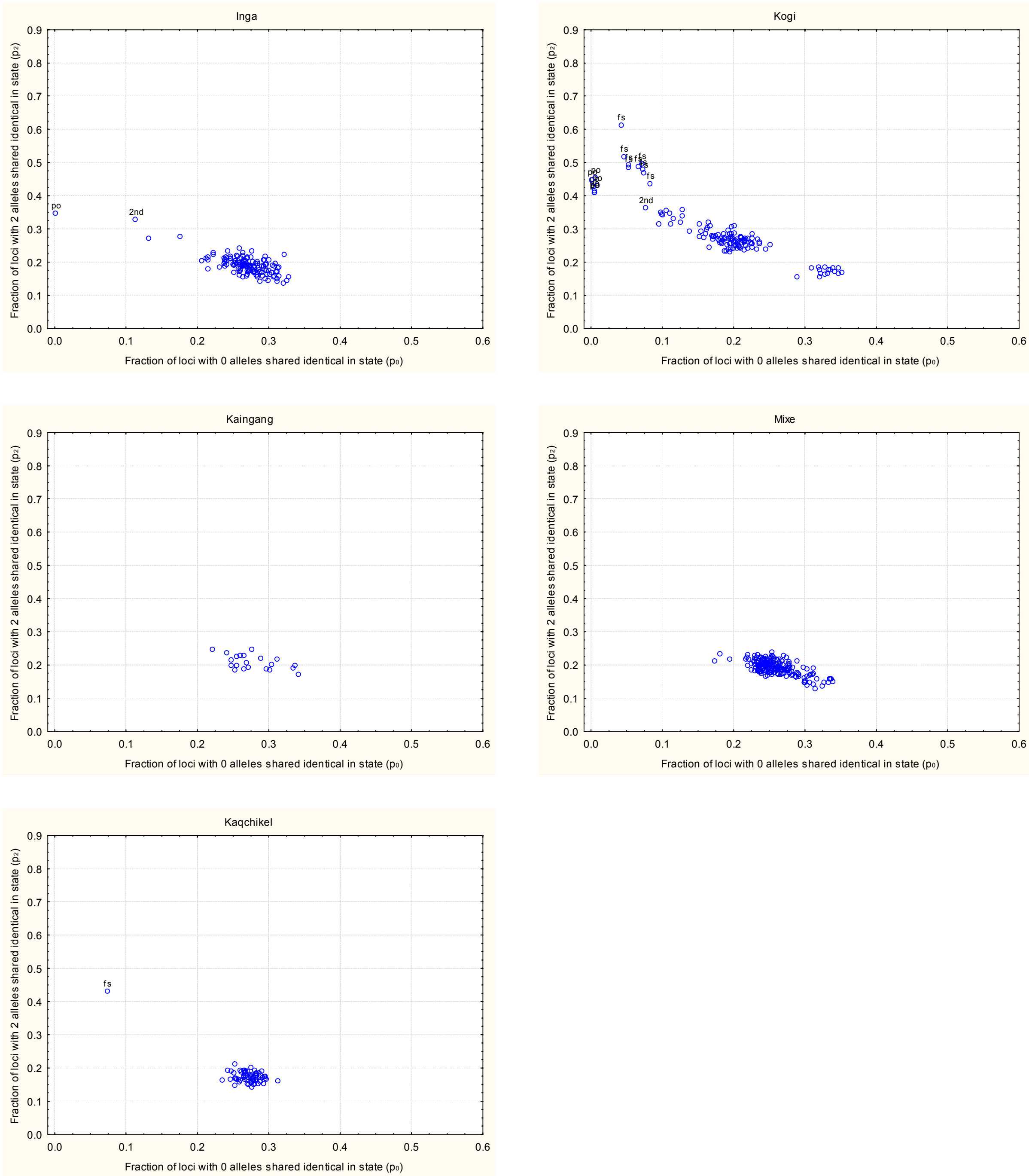
Inga
Kogi
Kaingang
Mixe
Kaqchikel
Fraction of loci with 2 alleles shared identical in state (p_2)
Fraction of loci with 0 alleles shared identical in state (p_0)
0.0
0.1
0.2
0.3
0.4
0.5
0.6
0.7
0.8
0.9
po
2nd
fs

Figure S11
