# Supplementary material for: Genetic Variation and Population Structure in Native Americans
Source: PLoS Genet. 2007 Nov 23;3(11):e185. doi: 10.1371/journal.pgen.0030185 (PMC2082466; doi:10.1371/journal.pgen.0030185)
Supplement: Supplementary file 12 [file pgen.0030185.sg012.pdf]

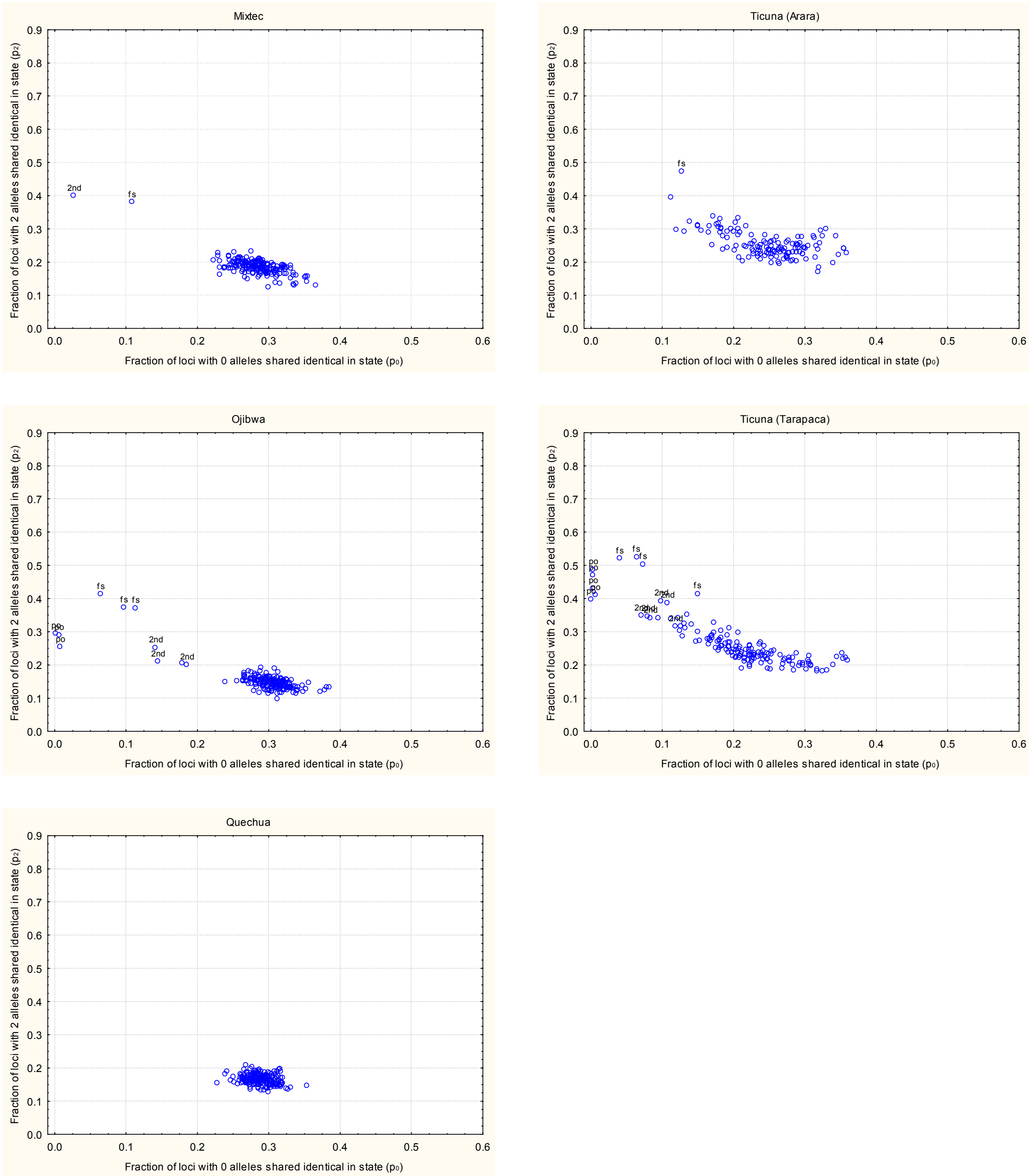
Mixtec
Ticuna (Arara)
Ojibwa
Ticuna (Tarapaca)
Quechua
Fraction of loci with 2 alleles shared identical in state (p2)
Fraction of loci with 0 alleles shared identical in state (p0)
2nd
fs
po

Figure S12
