# Supplementary material for: Genetic Variation and Population Structure in Native Americans
Source: PLoS Genet. 2007 Nov 23;3(11):e185. doi: 10.1371/journal.pgen.0030185 (PMC2082466; doi:10.1371/journal.pgen.0030185)
Supplement: Supplementary file 13 [file pgen.0030185.sg013.pdf]

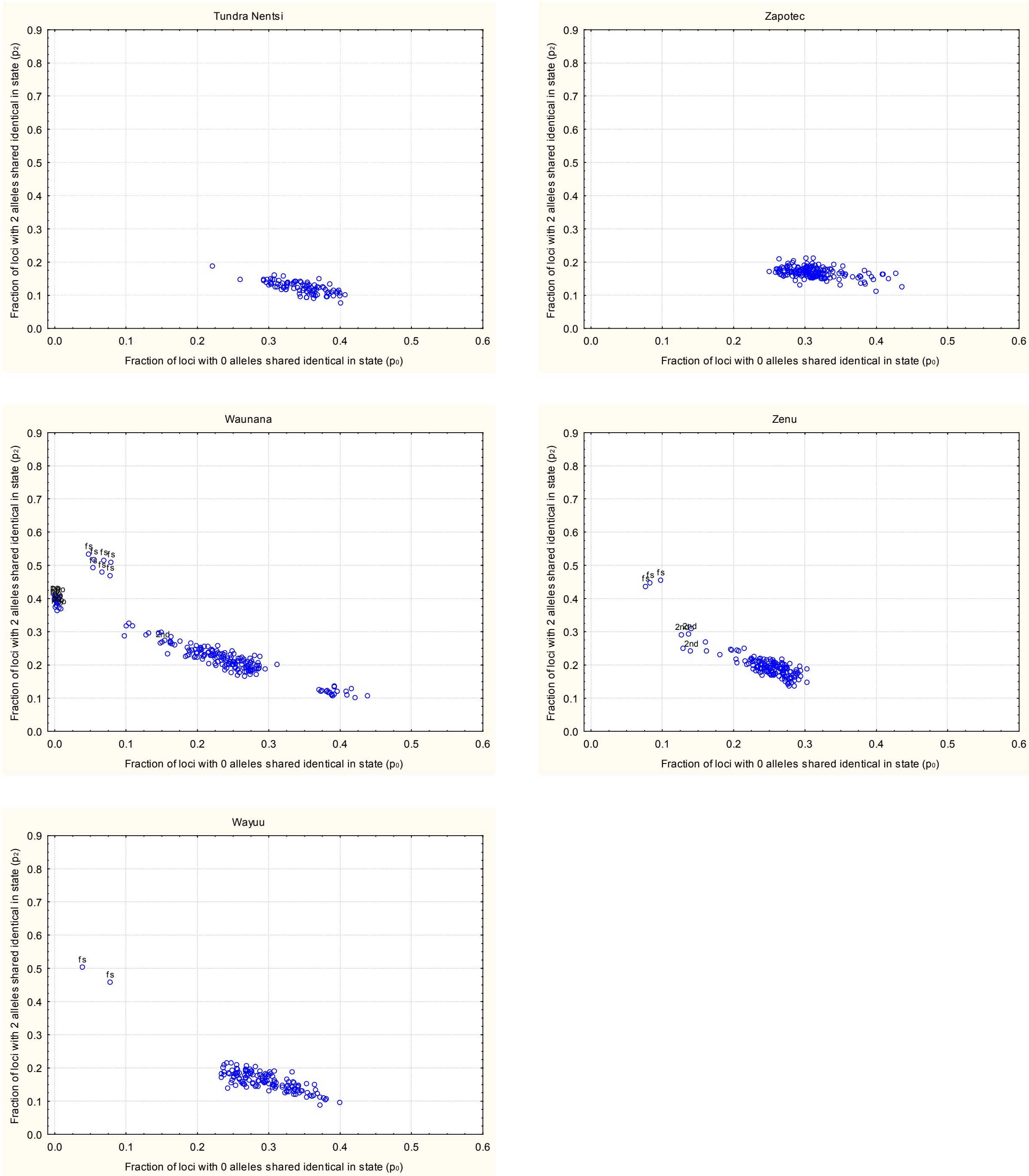

Tundra Nentsi
Zapotec
Waunana
Zenu
Wayuu
Fraction of loci with 2 alleles shared identical in state (p_2)
Fraction of loci with 0 alleles shared identical in state (p_0)
fs
2nd

Figure S13
