# Supplementary material for: Genetic Variation and Population Structure in Native Americans
Source: PLoS Genet. 2007 Nov 23;3(11):e185. doi: 10.1371/journal.pgen.0030185 (PMC2082466; doi:10.1371/journal.pgen.0030185)
Supplement: Supplementary file 14 [file pgen.0030185.sg014.pdf]

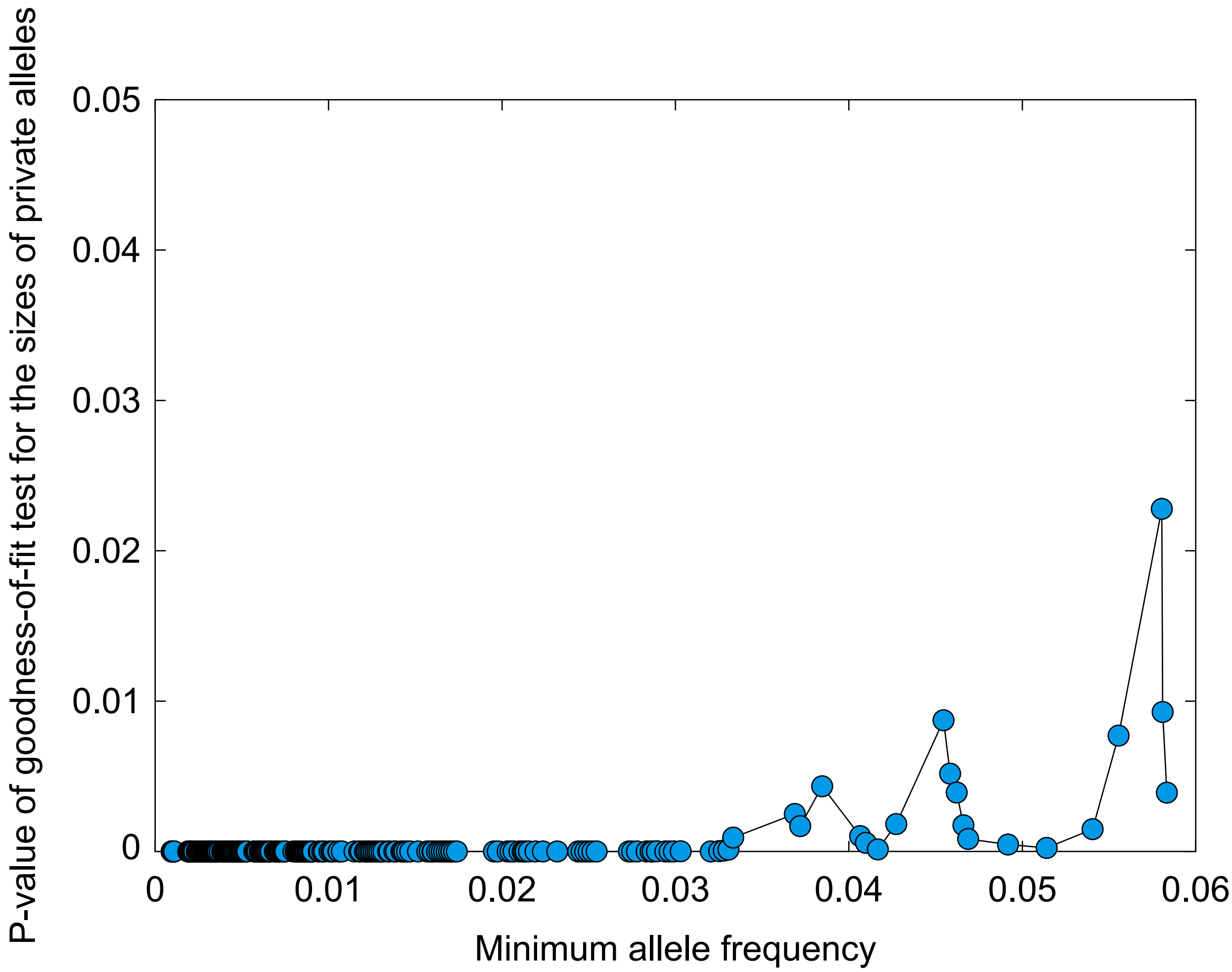
0.05
0.04
0.03
0.02
0.01
0
0
0.01
0.02
0.03
0.04
0.05
0.06
P-value of goodness-of-fit test for the sizes of private alleles
Minimum allele frequency

Figure S14
