# Supplementary material for: Genetic Variation and Population Structure in Native Americans
Source: PLoS Genet. 2007 Nov 23;3(11):e185. doi: 10.1371/journal.pgen.0030185 (PMC2082466; doi:10.1371/journal.pgen.0030185)
Supplement: Supplementary file 15 [file pgen.0030185.sg015.pdf]

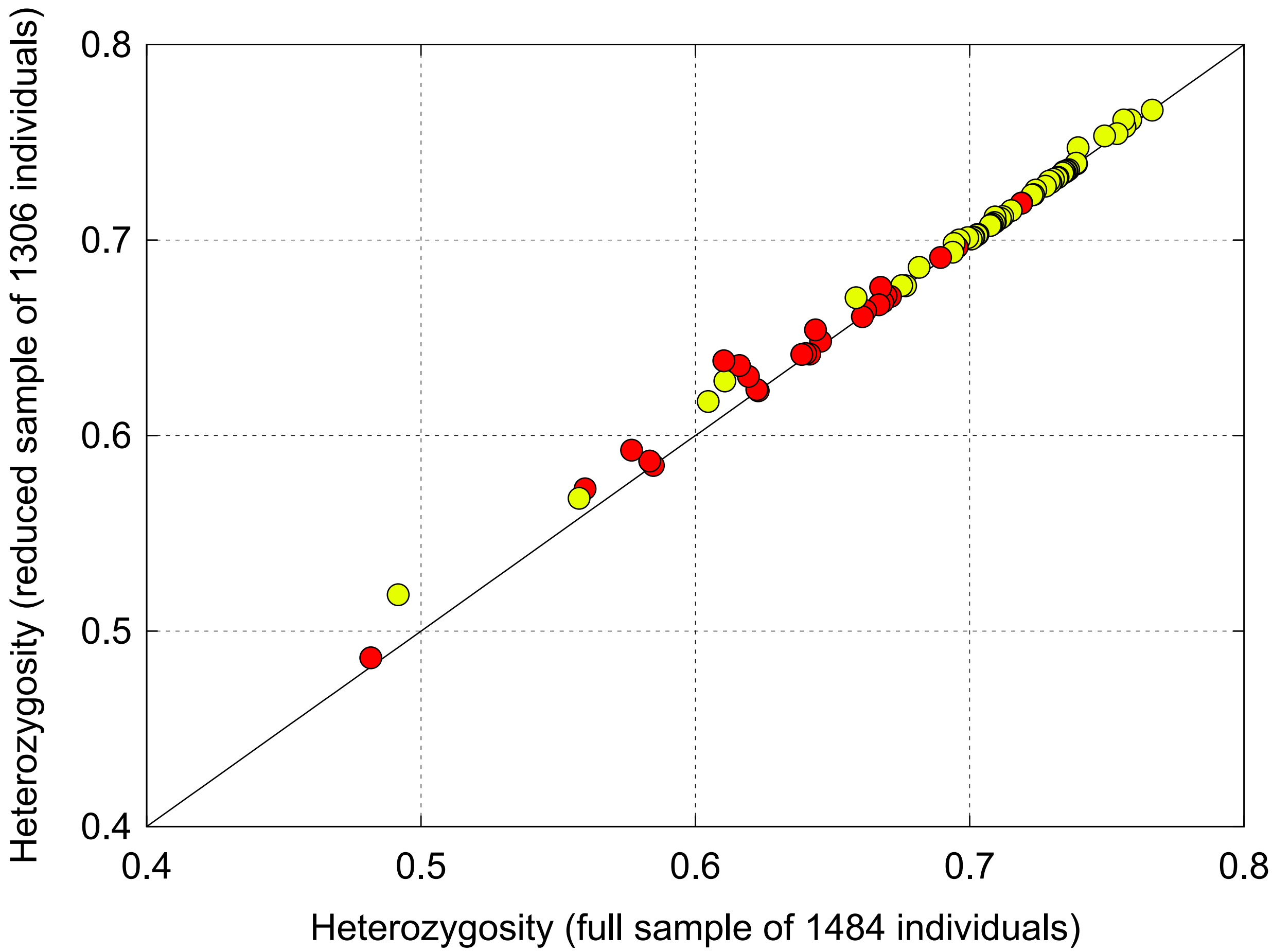

Heterozygosity (reduced sample of 1306 individuals)
0.8
0.7
0.6
0.5
0.4
0.4
0.5
0.6
0.7
0.8
Heterozygosity (full sample of 1484 individuals)


Figure S16
