# Supplementary material for: Genetic Variation and Population Structure in Native Americans
Source: PLoS Genet. 2007 Nov 23;3(11):e185. doi: 10.1371/journal.pgen.0030185 (PMC2082466; doi:10.1371/journal.pgen.0030185)
Supplement: Supplementary file 19 [file pgen.0030185.sg019.pdf]

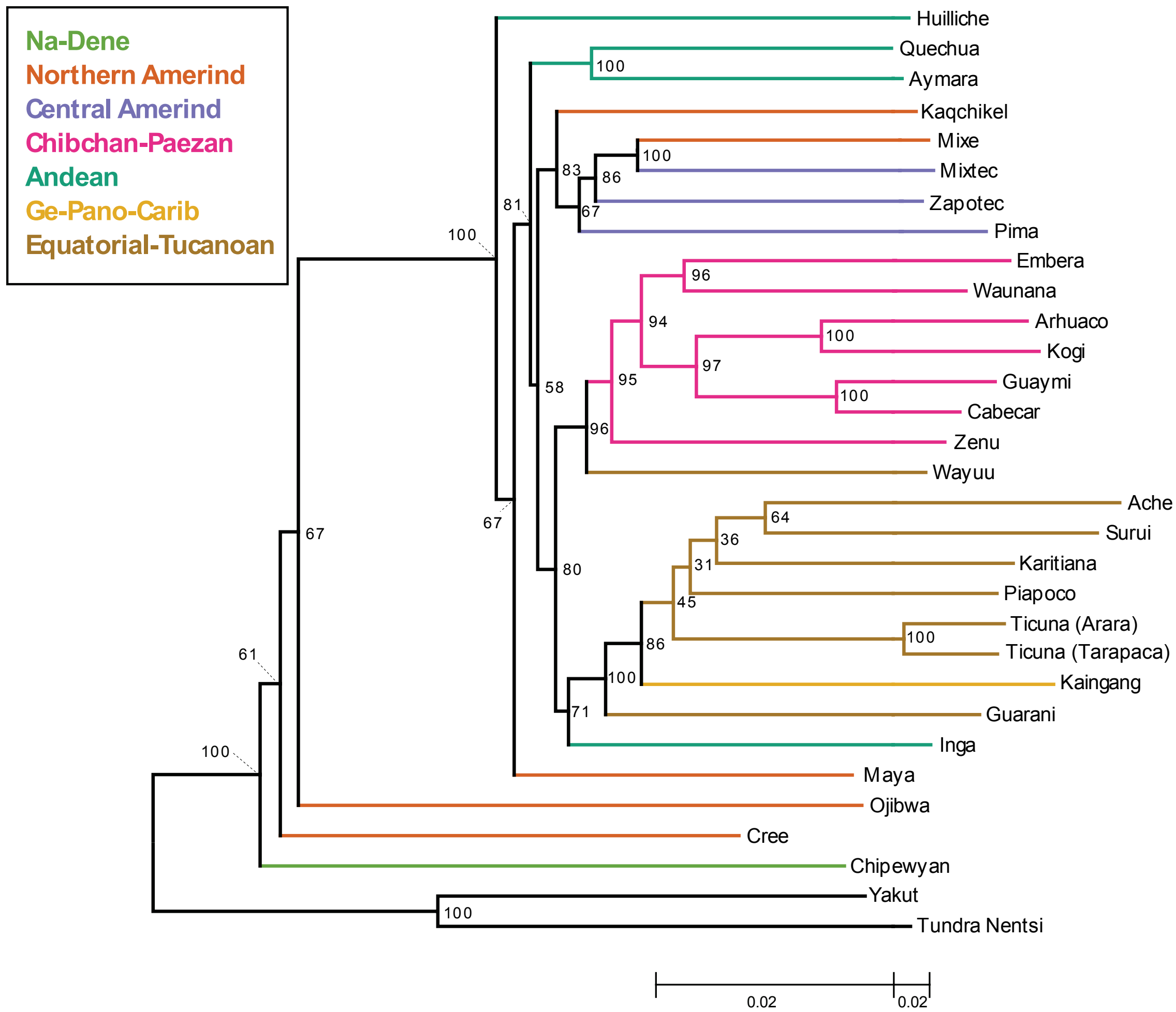
Na-Dene
Northern Amerind
Central Amerind
Chibchan-Paezan
Andean
Ge-Pano-Carib
Equatorial-Tucanoan
Huilliche
Quechua
Aymara
Kaqchikel
Mixe
Mixtec
Zapotec
Pima
Embera
Waunana
Arhuaco
Kogi
Guaymi
Cabecar
Zenu
Wayuu
Ache
Surui
Karitiana
Piapoco
Ticuna (Arara)
Ticuna (Tarapaca)
Kaingang
Guarani
Inga
Maya
Ojibwa
Cree
Chipewyan
Yakut
Tundra Nentsi
0.02
0.02

Figure S19
