# Supplementary material for: Genetic Variation and Population Structure in Native Americans
Source: PLoS Genet. 2007 Nov 23;3(11):e185. doi: 10.1371/journal.pgen.0030185 (PMC2082466; doi:10.1371/journal.pgen.0030185)
Supplement: Supplementary file 20 [file pgen.0030185.sg020.pdf]

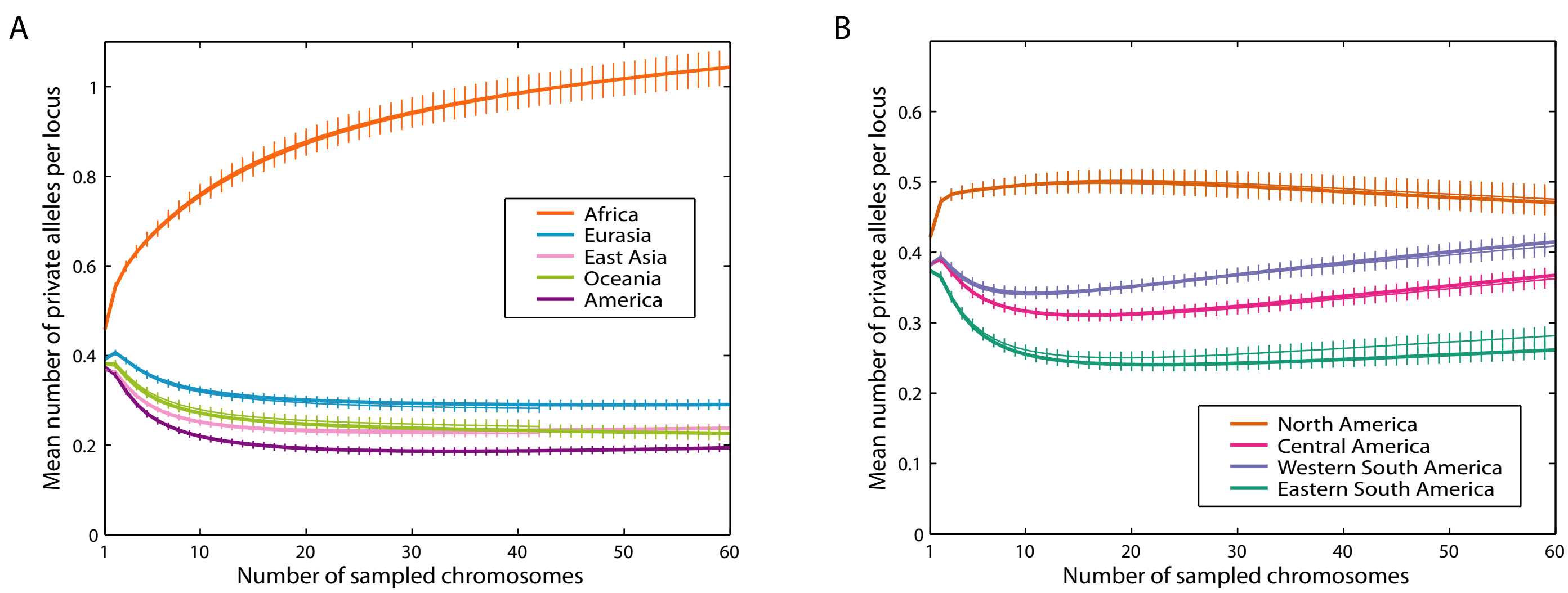

A
Mean number of private alleles per locus
Number of sampled chromosomes
Africa
Eurasia
East Asia
Oceania
America
B
North America
Central America
Western South America
Eastern South America

Figure S20
