# Supplementary material for: Genetic Variation and Population Structure in Native Americans
Source: PLoS Genet. 2007 Nov 23;3(11):e185. doi: 10.1371/journal.pgen.0030185 (PMC2082466; doi:10.1371/journal.pgen.0030185)
Supplement: Supplementary file 21 [file pgen.0030185.st001.pdf]

Table S1. Native American populations included in this study, and the coordinates used for their sampling locations.

Population name in the present dataset	Other names	Latitude (degrees north)	Longitude (degrees east)	Sample size
Chipewyan	Dene[1,2]	59.55	-107.3	29
Cree		50.33	-102.5	18
Ojibwa	Northern Ojibwa, Ojibway, Ojibwe[1,2]	46.5	-81	20
Pima	3	29	-108	25
Mixtec	Nudzahui	17	-97	20
Zapotec	3	16	-97	19
Mixe	3	17	-96	20
Maya	3	19	-91	25
Kaqchikel	Cakchiquel, Maya, Quiché	15	-91	12
Cabecar	Chirripó[1,2]	9.5	-84	20
Guaymi		8.5	-82	18
Kogi	Coghui, Cogui, Kagaba, Kaggaba, Kogui[1,2]	11	-74	17
Arhuaco	Aruaco, Bintucua, Bintuk, Bíntukua, Ica, Ijca, Ijka, Ika, Ike[1,2]	11	-73.8	17
Waunana	Chanco, Chocama, Noanama, Noenama, Nonama, Waumeo, Waun Meo, Woun Meu, Wounaan, Wounmeu[1,2]	5	-77	20
Embera	Atrato, Cholo, Darien, Dariena, Ebera Bedea, Eerã, Empera[1,2]	7	-76	11
Zenu	Senu, Sinu[4]	9	-75	18
Inga	Ingano, Lowland Inga, Mocoa, Quechua II B[1,2]	1	-77	17
Quechua	3	-14	-74	20
Aymara	3	-22	-70	18
Huilliche	Huiliche, Veliche[1,2]	-41	-73	20
Kaingang	Bugre, Caingang, Coroado, Coroados[1,2]	-24	-52.5	7
Guarani	3	-23	-54	10
Wayuu	Goajiro, Guajira, Guajiro[1,2]	11	-73	17
Piapoco	Dzaze, Piapoko[1,2]	3	-68	13
Ticuna (Tarapaca)	Magüta, Tikuna, Tucuna, Tukuna, Tukúna[1,2] (more northerly of the two Ticuna samples, collected 1996)	-4	-70	18
Ticuna (Arara)	Magüta, Tikuna,Tucuna, Tukuna, Tukúna[1,2] (more southerly of the two Ticuna samples, collected 1995)	-4	-70	17
Karitiana	Caritiana[2], Karitiâna[1]	-10	-63	24
Surui	Paiter[1,2]	-11	-62	21
Ache	Axe	-24	-56	19

[1]Appleby M, Sawyer A (2006) *The Linguist List*. http://www.linguistlist.org.
[2]Gordon RGJ (2005) *Ethnologue: Languages of the World*, Fifteenth edition. Dallas: SIL International: http://www.ethnologue.com.
[3]The are several regional dialects, some with different common names.
[4]Ruiz-Linares A, et al. (1999) Microsatellites provide evidence for Y chromosome diversity among the founders of the New World. *Proc Natl Acad Sci U S A* 96: 6312-6317.
The Tundra Nentsi sample new in this data set had size 14, and was located at 66.08ºN 76.5ºE.
