# Supplementary material for: Genetic Variation and Population Structure in Native Americans
Source: PLoS Genet. 2007 Nov 23;3(11):e185. doi: 10.1371/journal.pgen.0030185 (PMC2082466; doi:10.1371/journal.pgen.0030185)
Supplement: Supplementary file 22 [file pgen.0030185.st002.pdf]

Table S2. Languages of the Native American populations included in this study, classified according to Greenberg (1987) and Ruhlen (1991).

**Population**	**Family**	**Stock**	**Group**	**Subgroup (level A)**	**Subgroup (level B)**	**Subgroup (level C)**	**Subgroup (level D)**
Chipewyan	Na-Dene	Continental Na-Dene	Athabaskan-Eyak	Athabaskan			
Cree	Amerind	Northern Amerind	Almosan-Keresiouan	Almosan	Algic	Algonquian	
Ojibwa	Amerind	Northern Amerind	Almosan-Keresiouan	Almosan	Algic	Algonquian	
Pima	Amerind	Central Amerind	Uto-Aztecan				
Mixtec	Amerind	Central Amerind	Oto-Mangue				
Zapotec	Amerind	Central Amerind	Oto-Mangue				
Mixe	Amerind	Northern Amerind	Penutian	Mexican	Mixe-Zoque		
Maya	Amerind	Northern Amerind	Penutian	Mexican	Mayan		
Kaqchikel	Amerind	Northern Amerind	Penutian	Mexican	Mayan		
Cabecar	Amerind	Chibchan-Paezan	Chibchan	Nuclear Chibchan	Talamanca		
Guaymi	Amerind	Chibchan-Paezan	Chibchan	Nuclear Chibchan	Guaymi		
Kogi	Amerind	Chibchan-Paezan	Chibchan	Nuclear Chibchan	Aruak		
Arhuaco	Amerind	Chibchan-Paezan	Chibchan	Nuclear Chibchan	Aruak		
Waunana	Amerind	Chibchan-Paezan	Paezan	Nuclear Paezan	Choco		
Embera	Amerind	Chibchan-Paezan	Paezan	Nuclear Paezan	Choco		
Zenu	Amerind	Chibchan-Paezan	Chibchan*				
Inga	Amerind	Andean	Quechua				
Quechua	Amerind	Andean	Quechua				
Aymara	Amerind	Andean	Aymara				
Huilliche	Amerind	Andean	Southern	Araucanian			
Kaingang	Amerind	Ge-Pano-Carib	Macro-Ge	Ge-Kaingang			
Guarani	Amerind	Equatorial-Tucanoan	Equatorial	Kariri-Tupi	Tupi	Guarani	
Wayuu	Amerind	Equatorial-Tucanoan	Equatorial	Macro-Arawakan	Arawakan	Maipuran	Goajiro
Piapoco	Amerind	Equatorial-Tucanoan	Equatorial	Macro-Arawakan	Arawakan	Maipuran	
Ticuna (Tarapaca)	Amerind	Equatorial-Tucanoan	Macro-Tucanoan	Ticuna-Yuri	Ticuna		
Ticuna (Arara)	Amerind	Equatorial-Tucanoan	Macro-Tucanoan	Ticuna-Yuri	Ticuna		
Karitiana	Amerind	Equatorial-Tucanoan	Equatorial	Kariri-Tupi	Tupi	Guarani	
Surui	Amerind	Equatorial-Tucanoan	Equatorial	Kariri-Tupi	Tupi	Monde	
Ache	Amerind	Equatorial-Tucanoan	Equatorial	Kariri-Tupi	Tupi	Guarani	

*This population has lost its ancestral language. Following Mesa et al. (2000), we classify it as belonging to the Chibchan group. We assume Zenu has its own subgroup within Chibchan.

Mesa NR, et al. (2000) Autosomal, mtDNA, and Y-chromosome diversity in Amerinds: pre- and post-Columbian patterns of gene flow in South America. *Am J Hum Genet* 67: 1277-1286.
