# Supplementary material for: Genetic Variation and Population Structure in Native Americans
Source: PLoS Genet. 2007 Nov 23;3(11):e185. doi: 10.1371/journal.pgen.0030185 (PMC2082466; doi:10.1371/journal.pgen.0030185)
Supplement: Supplementary file 23 [file pgen.0030185.st003.pdf]

Table S3. Discretized matrix of linguistic distances for pairs of populations, with all stocks treated as equidistant.

		I	II	III	IV	V	VI	VII	VIII	IX	X	XI	XII	XIII	XIV	XV	XVI	XVII	XVIII	XIX	XX	XXI	XXII	XXIII	XXIV	XXV	XXVI	XXVII	XXVIII	XXIX
I	Chipewyan	0	4	4	4	4	4	4	4	4	4	4	4	4	4	4	4	4	4	4	4	4	4	4	4	4	4	4	4	4
II	Cree	4	0	2	4	4	4	4	4	4	4	4	4	4	4	4	4	4	4	4	4	4	4	4	4	4	4	4	4	4
III	Ojibwa	4	2	0	4	4	4	4	4	4	4	4	4	4	4	4	4	4	4	4	4	4	4	4	4	4	4	4	4	4
IV	Pima	4	4	4	0	4	4	4	4	4	4	4	4	4	4	4	4	4	4	4	4	4	4	4	4	4	4	4	4	4
V	Mixtec	4	4	4	4	0	2	4	4	4	4	4	4	4	4	4	4	4	4	4	4	4	4	4	4	4	4	4	4	4
VI	Zapotec	4	4	4	4	2	0	4	4	4	4	4	4	4	4	4	4	4	4	4	4	4	4	4	4	4	4	4	4	4
VII	Mixe	4	4	4	4	4	4	0	3	3	4	4	4	4	4	4	4	4	4	4	4	4	4	4	4	4	4	4	4	4
VIII	Maya	4	4	4	4	4	4	3	0	2	4	4	4	4	4	4	4	4	4	4	4	4	4	4	4	4	4	4	4	4
IX	Kaqchikel	4	4	4	4	4	4	3	2	0	4	4	4	4	4	4	4	4	4	4	4	4	4	4	4	4	4	4	4	4
X	Cabecar	4	4	4	4	4	4	4	4	4	0	3	3	3	4	4	3	4	4	4	4	4	4	4	4	4	4	4	4	4
XI	Guaymi	4	4	4	4	4	4	4	4	4	3	0	3	3	4	4	3	4	4	4	4	4	4	4	4	4	4	4	4	4
XII	Kogi	4	4	4	4	4	4	4	4	4	3	3	0	2	4	4	3	4	4	4	4	4	4	4	4	4	4	4	4	4
XIII	Arhuaco	4	4	4	4	4	4	4	4	4	3	3	2	0	4	4	3	4	4	4	4	4	4	4	4	4	4	4	4	4
XIV	Waunana	4	4	4	4	4	4	4	4	4	4	4	4	4	0	2	4	4	4	4	4	4	4	4	4	4	4	4	4	4
XV	Embera	4	4	4	4	4	4	4	4	4	4	4	4	4	2	0	4	4	4	4	4	4	4	4	4	4	4	4	4	4
XVI	Zenu	4	4	4	4	4	4	4	4	4	3	3	3	3	4	4	0	4	4	4	4	4	4	4	4	4	4	4	4	4
XVII	Inga	4	4	4	4	4	4	4	4	4	4	4	4	4	4	4	4	0	2	3	3	4	4	4	4	4	4	4	4	4
XVIII	Quechua	4	4	4	4	4	4	4	4	4	4	4	4	4	4	4	4	2	0	3	3	4	4	4	4	4	4	4	4	4
XIX	Aymara	4	4	4	4	4	4	4	4	4	4	4	4	4	4	4	4	3	3	0	3	4	4	4	4	4	4	4	4	4
XX	Huilliche	4	4	4	4	4	4	4	4	4	4	4	4	4	4	4	4	3	3	3	0	4	4	4	4	4	4	4	4	4
XXI	Kaingang	4	4	4	4	4	4	4	4	4	4	4	4	4	4	4	4	4	4	4	4	0	4	4	4	4	4	4	4	4
XXII	Guarani	4	4	4	4	4	4	4	4	4	4	4	4	4	4	4	4	4	4	4	4	4	0	3	3	4	4	2	3	2
XXIII	Wayuu	4	4	4	4	4	4	4	4	4	4	4	4	4	4	4	4	4	4	4	4	4	3	0	2	4	4	3	3	3
XXIV	Piapoco	4	4	4	4	4	4	4	4	4	4	4	4	4	4	4	4	4	4	4	4	4	3	2	0	4	4	3	3	3
XXV	Ticuna (Tarapaca)	4	4	4	4	4	4	4	4	4	4	4	4	4	4	4	4	4	4	4	4	4	4	4	4	0	1	4	4	4
XXVI	Ticuna (Arara)	4	4	4	4	4	4	4	4	4	4	4	4	4	4	4	4	4	4	4	4	4	4	4	4	1	0	4	4	4
XXVII	Karitiana	4	4	4	4	4	4	4	4	4	4	4	4	4	4	4	4	4	4	4	4	4	2	3	3	4	4	0	3	2
XXVIII	Surui	4	4	4	4	4	4	4	4	4	4	4	4	4	4	4	4	4	4	4	4	4	3	3	3	4	4	3	0	3
XXIX	Ache	4	4	4	4	4	4	4	4	4	4	4	4	4	4	4	4	4	4	4	4	4	2	3	3	4	4	2	3	0

All between-stock comparisons are highlighted.
