# Supplementary material for: Genetic Variation and Population Structure in Native Americans
Source: PLoS Genet. 2007 Nov 23;3(11):e185. doi: 10.1371/journal.pgen.0030185 (PMC2082466; doi:10.1371/journal.pgen.0030185)
Supplement: Supplementary file 24 [file pgen.0030185.st004.pdf]

Table S4. Number of etymologies shared between language stocks, among the 281 etymologies examined by Greenberg (1987).

	Almosan-Keresiouan	Penutian	Hokan	Central Amerind	Chibchan-Paezan	Andean	Macro-Tucanoan	Equatorial	Macro-Carib	Macro-Panoan	Macro-Ge
Almosan-Keresiouan	87	47	44	26	35	21	24	28	12	32	29
Penutian	47	106	50	36	40	32	30	33	20	24	33
Hokan	44	50	101	24	36	27	30	31	18	27	38
Central Amerind	26	36	24	71	33	20	28	25	20	24	27
Chibchan-Paezan	35	40	36	33	100	32	28	35	22	29	34
Andean	21	32	27	20	32	85	22	28	22	28	31
Macro-Tucanoan	24	30	30	28	28	22	85	41	17	29	39
Equatorial	28	33	31	25	35	28	41	96	21	28	39
Macro-Carib	12	20	18	20	22	22	17	21	66	27	33
Macro-Panoan	32	24	27	24	29	28	29	28	27	79	46
Macro-Ge	29	33	38	27	34	31	39	39	33	46	103

All entries marked “X” or “(X)” in Appendix 4 of Greenberg (1987) were tabulated as shared etymologies.
