# Supplementary material for: Genetic Variation and Population Structure in Native Americans
Source: PLoS Genet. 2007 Nov 23;3(11):e185. doi: 10.1371/journal.pgen.0030185 (PMC2082466; doi:10.1371/journal.pgen.0030185)
Supplement: Supplementary file 25 [file pgen.0030185.st005.pdf]

Table S5. Number of etymologies unshared between language stocks, among the 281 etymologies examined by Greenberg (1987).

	Almosan-Keresiouan	Penutian	Hokan	Central Amerind	Chibchan-Paezan	Andean	Macro-Tucanoan	Equatorial	Macro-Carib	Macro-Panoan	Macro-Ge
Almosan-Keresiouan	87	59	57	45	65	64	61	68	54	47	74
Penutian	40	106	51	35	60	53	55	63	46	55	70
Hokan	43	56	101	47	64	58	55	65	48	52	65
Central Amerind	61	70	77	71	67	65	57	71	46	55	76
Chibchan-Paezan	52	66	65	38	100	53	57	61	44	50	69
Andean	66	74	74	51	68	85	63	68	44	51	72
Macro-Tucanoan	63	76	71	43	72	63	85	55	49	50	64
Equatorial	59	73	70	46	65	57	44	96	45	51	64
Macro-Carib	75	86	83	51	78	63	68	75	66	52	70
Macro-Panoan	55	82	74	47	71	57	56	68	39	79	57
Macro-Ge	58	73	63	44	66	54	46	57	33	33	103

The upper triangle gives the number of etymologies found in the stock in the appropriate column but absent in the stock in the appropriate row. The lower triangle gives the number of etymologies found in the stock in the appropriate row but absent in the stock in the appropriate column. The diagonal gives the total number of etymologies identified for the appropriate stock.
