# Supplementary material for: Genetic Variation and Population Structure in Native Americans
Source: PLoS Genet. 2007 Nov 23;3(11):e185. doi: 10.1371/journal.pgen.0030185 (PMC2082466; doi:10.1371/journal.pgen.0030185)
Supplement: Supplementary file 26 [file pgen.0030185.st006.pdf]

Table S6. Matrix of linguistic distances between stocks, based on the Dice index.

	Almosan-Keresiouan	Penutian	Hokan	Central Amerind	Chibchan-Paezan	Andean	Macro-Tucanoan	Equatorial	Macro-Carib	Macro-Panoan	Macro-Ge
Almosan-Keresiouan	0.0000	0.5130	0.5319	0.6709	0.6257	0.7558	0.7209	0.6940	0.8431	0.6145	0.6947
Penutian	0.5130	0.0000	0.5169	0.5932	0.6117	0.6649	0.6859	0.6733	0.7674	0.7405	0.6842
Hokan	0.5319	0.5169	0.0000	0.7209	0.6418	0.7097	0.6774	0.6853	0.7844	0.7000	0.6275
Central Amerind	0.6709	0.5932	0.7209	0.0000	0.6140	0.7436	0.6410	0.7006	0.7080	0.6800	0.6897
Chibchan-Paezan	0.6257	0.6117	0.6418	0.6140	0.0000	0.6541	0.6973	0.6429	0.7349	0.6760	0.6650
Andean	0.7558	0.6649	0.7097	0.7436	0.6541	0.0000	0.7412	0.6906	0.7086	0.6585	0.6702
Macro-Tucanoan	0.7209	0.6859	0.6774	0.6410	0.6973	0.7412	0.0000	0.5470	0.7748	0.6463	0.5851
Equatorial	0.6940	0.6733	0.6853	0.7006	0.6429	0.6906	0.5470	0.0000	0.7407	0.6800	0.6080
Macro-Carib	0.8431	0.7674	0.7844	0.7080	0.7349	0.7086	0.7748	0.7407	0.0000	0.6276	0.6095
Macro-Panoan	0.6145	0.7405	0.7000	0.6800	0.6760	0.6585	0.6463	0.6800	0.6276	0.0000	0.4945
Macro-Ge	0.6947	0.6842	0.6275	0.6897	0.6650	0.6702	0.5851	0.6080	0.6095	0.4945	0.0000

This matrix was obtained from Tables S4 and S5 using the Dice index of dissimilarity for groups A and B, $D=(M_{01}+M_{10})/(M_{01}+M_{10}+2M_{11})$, where M_{01}, M_{10}, and M_{11} respectively represent the number of etymologies absent in group A and present in group B, the number of etymologies present in group A and absent in group B, and the number of etymologies present in both groups.
