# Supplementary material for: Genetic Variation and Population Structure in Native Americans
Source: PLoS Genet. 2007 Nov 23;3(11):e185. doi: 10.1371/journal.pgen.0030185 (PMC2082466; doi:10.1371/journal.pgen.0030185)
Supplement: Supplementary file 27 [file pgen.0030185.st007.pdf]

Table S7. Matrix of linguistic distances between stocks, based on the Jaccard index.

	Almosan-Keresiouan	Penutian	Hokan	Central Amerind	Chibchan-Paezan	Andean	Macro-Tucanoan	Equatorial	Macro-Carib	Macro-Panoan	Macro-Ge
Almosan-Keresiouan	0.0000	0.6781	0.6944	0.8030	0.7697	0.8609	0.8378	0.8194	0.9149	0.7612	0.8199
Penutian	0.6781	0.0000	0.6815	0.7447	0.7590	0.7987	0.8137	0.8047	0.8684	0.8509	0.8125
Hokan	0.6944	0.6815	0.0000	0.8378	0.7818	0.8302	0.8077	0.8133	0.8792	0.8235	0.7711
Central Amerind	0.8030	0.7447	0.8378	0.0000	0.7609	0.8529	0.7813	0.8239	0.8291	0.8095	0.8163
Chibchan-Paezan	0.7697	0.7590	0.7818	0.7609	0.0000	0.7908	0.8217	0.7826	0.8472	0.8067	0.7988
Andean	0.8609	0.7987	0.8302	0.8529	0.7908	0.0000	0.8514	0.8170	0.8295	0.7941	0.8025
Macro-Tucanoan	0.8378	0.8137	0.8077	0.7813	0.8217	0.8514	0.0000	0.7071	0.8731	0.7852	0.7383
Equatorial	0.8194	0.8047	0.8133	0.8239	0.7826	0.8170	0.7071	0.0000	0.8511	0.8095	0.7563
Macro-Carib	0.9149	0.8684	0.8792	0.8291	0.8472	0.8295	0.8731	0.8511	0.0000	0.7712	0.7574
Macro-Panoan	0.7612	0.8509	0.8235	0.8095	0.8067	0.7941	0.7852	0.8095	0.7712	0.0000	0.6618
Macro-Ge	0.8199	0.8125	0.7711	0.8163	0.7988	0.8025	0.7383	0.7563	0.7574	0.6618	0.0000

This matrix was obtained from Tables S4 and S5 using the Jaccard index of dissimilarity for groups A and B, $J=(M_{01}+M_{10})/(M_{01}+M_{10}+M_{11})$, where M_{01}, M_{10}, and M_{11} respectively represent the number of etymologies absent in group A and present in group B, the number of etymologies present in group A and absent in group B, and the number of etymologies present in both groups.
