# Supplementary material for: Genetic Variation and Population Structure in Native Americans
Source: PLoS Genet. 2007 Nov 23;3(11):e185. doi: 10.1371/journal.pgen.0030185 (PMC2082466; doi:10.1371/journal.pgen.0030185)
Supplement: Supplementary file 29 [file pgen.0030185.st009.pdf]

Table S9. Discretized matrix of linguistic distances between stocks, based on the Jaccard index.

	Almosan-Keresiouan	Central Amerind	Penutian	Chibchan-Paezan	Andean	Macro-Ge	Equatorial	Macro-Tucanoan
Almosan-Keresiouan	NA	7.5	NA	6.0	10.0	8.5	8.5	9.0
Central Amerind	7.5	NA	5.0	5.5	9.5	8.5	8.5	6.5
Penutian	NA	5.0	NA	5.5	7.5	8.0	7.5	8.0
Chibchan-Paezan	6.0	5.5	5.5	NA	7.0	7.5	6.5	8.5
Andean	10.0	9.5	7.5	7.0	NA	7.5	8.5	9.5
Macro-Ge	8.5	8.5	8.0	7.5	7.5	NA	5.5	5.0
Equatorial	8.5	8.5	7.5	6.5	8.5	5.5	NA	NA
Macro-Tucanoan	9.0	6.5	8.0	8.5	9.5	5.0	NA	NA

This matrix was obtained from Table S7 by suppressing stocks not represented in our data set and comparisons connecting stocks that are combined by Greenberg (1987) into higher-level stocks. The Almosan-Keresiouan, Penutian, and Hokan stocks (“groups” in Table S2) are grouped as Northern Amerind; Macro-Tucanoan and Equatorial are grouped as Equatorial-Tucanoan; Macro-Carib, Macro-Panoan, and Macro-Ge are grouped as Ge-Pano-Carib. The remaining entries were rounded to two decimal places and the smallest value was associated with linguistic distance 5.0. Higher values were ordered and were sequentially associated with larger distances at intervals of 0.5, with values that were tied after rounding being given the same discretized distance.
