# Supplementary material for: Genetic Variation and Population Structure in Native Americans
Source: PLoS Genet. 2007 Nov 23;3(11):e185. doi: 10.1371/journal.pgen.0030185 (PMC2082466; doi:10.1371/journal.pgen.0030185)
Supplement: Supplementary file 31 [file pgen.0030185.st011.pdf]

Table S11. Discretized matrix of linguistic distances for pairs of populations, with stock distances based on the Jaccard index.

		I	II	III	IV	V	VI	VII	VIII	IX	X	XI	XII	XIII	XIV	XV	XVI	XVII	XVIII	XIX	XX	XXI	XXII	XXIII	XXIV	XXV	XXVI	XXVII	XXVIII
I	Cree	0	2	7.5	7.5	7.5	4	4	4	6	6	6	6	6	6	6	10	10	10	10	8.5	8.5	8.5	8.5	9	9	8.5	8.5	8.5
II	Ojibwa	2	0	7.5	7.5	7.5	4	4	4	6	6	6	6	6	6	6	10	10	10	10	8.5	8.5	8.5	8.5	9	9	8.5	8.5	8.5
III	Pima	7.5	7.5	0	4	4	5	5	5	5.5	5.5	5.5	5.5	5.5	5.5	5.5	9.5	9.5	9.5	9.5	8.5	8.5	8.5	8.5	6.5	6.5	8.5	8.5	8.5
IV	Mixtec	7.5	7.5	4	0	2	5	5	5	5.5	5.5	5.5	5.5	5.5	5.5	5.5	9.5	9.5	9.5	9.5	8.5	8.5	8.5	8.5	6.5	6.5	8.5	8.5	8.5
V	Zapotec	7.5	7.5	4	2	0	5	5	5	5.5	5.5	5.5	5.5	5.5	5.5	5.5	9.5	9.5	9.5	9.5	8.5	8.5	8.5	8.5	6.5	6.5	8.5	8.5	8.5
VI	Mixe	4	4	5	5	5	0	3	3	5.5	5.5	5.5	5.5	5.5	5.5	5.5	7.5	7.5	7.5	7.5	8	7.5	7.5	7.5	8	8	7.5	7.5	7.5
VII	Maya	4	4	5	5	5	3	0	2	5.5	5.5	5.5	5.5	5.5	5.5	5.5	7.5	7.5	7.5	7.5	8	7.5	7.5	7.5	8	8	7.5	7.5	7.5
VIII	Kaqchikel	4	4	5	5	5	3	2	0	5.5	5.5	5.5	5.5	5.5	5.5	5.5	7.5	7.5	7.5	7.5	8	7.5	7.5	7.5	8	8	7.5	7.5	7.5
IX	Cabecar	6	6	5.5	5.5	5.5	5.5	5.5	5.5	0	3	3	3	4	4	3	7	7	7	7	7.5	6.5	6.5	6.5	8.5	8.5	6.5	6.5	6.5
X	Guaymi	6	6	5.5	5.5	5.5	5.5	5.5	5.5	3	0	3	3	4	4	3	7	7	7	7	7.5	6.5	6.5	6.5	8.5	8.5	6.5	6.5	6.5
XI	Kogi	6	6	5.5	5.5	5.5	5.5	5.5	5.5	3	3	0	2	4	4	3	7	7	7	7	7.5	6.5	6.5	6.5	8.5	8.5	6.5	6.5	6.5
XII	Arhuaco	6	6	5.5	5.5	5.5	5.5	5.5	5.5	3	3	2	0	4	4	3	7	7	7	7	7.5	6.5	6.5	6.5	8.5	8.5	6.5	6.5	6.5
XIII	Waunana	6	6	5.5	5.5	5.5	5.5	5.5	5.5	4	4	4	4	0	2	4	7	7	7	7	7.5	6.5	6.5	6.5	8.5	8.5	6.5	6.5	6.5
XIV	Embera	6	6	5.5	5.5	5.5	5.5	5.5	5.5	4	4	4	4	2	0	4	7	7	7	7	7.5	6.5	6.5	6.5	8.5	8.5	6.5	6.5	6.5
XV	Zenu	6	6	5.5	5.5	5.5	5.5	5.5	5.5	3	3	3	3	4	4	0	7	7	7	7	7.5	6.5	6.5	6.5	8.5	8.5	6.5	6.5	6.5
XVI	Inga	10	10	9.5	9.5	9.5	7.5	7.5	7.5	7	7	7	7	7	7	7	0	2	3	3	7.5	8.5	8.5	8.5	9.5	9.5	8.5	8.5	8.5
XVII	Quechua	10	10	9.5	9.5	9.5	7.5	7.5	7.5	7	7	7	7	7	7	7	2	0	3	3	7.5	8.5	8.5	8.5	9.5	9.5	8.5	8.5	8.5
XVIII	Aymara	10	10	9.5	9.5	9.5	7.5	7.5	7.5	7	7	7	7	7	7	7	3	3	0	3	7.5	8.5	8.5	8.5	9.5	9.5	8.5	8.5	8.5
XIX	Huilliche	10	10	9.5	9.5	9.5	7.5	7.5	7.5	7	7	7	7	7	7	7	3	3	3	0	7.5	8.5	8.5	8.5	9.5	9.5	8.5	8.5	8.5
XX	Kaingang	8.5	8.5	8.5	8.5	8.5	8	8	8	7.5	7.5	7.5	7.5	7.5	7.5	7.5	7.5	7.5	7.5	7.5	0	5.5	5.5	5.5	5	5	5.5	5.5	5.5
XXI	Guarani	8.5	8.5	8.5	8.5	8.5	7.5	7.5	7.5	6.5	6.5	6.5	6.5	6.5	6.5	6.5	8.5	8.5	8.5	8.5	5.5	0	3	3	4	4	2	3	2
XXII	Wayuu	8.5	8.5	8.5	8.5	8.5	7.5	7.5	7.5	6.5	6.5	6.5	6.5	6.5	6.5	6.5	8.5	8.5	8.5	8.5	5.5	3	0	2	4	4	3	3	3
XXIII	Piapoco	8.5	8.5	8.5	8.5	8.5	7.5	7.5	7.5	6.5	6.5	6.5	6.5	6.5	6.5	6.5	8.5	8.5	8.5	8.5	5.5	3	2	0	4	4	3	3	3
XXIV	Ticuna (Tarapaca)	9	9	6.5	6.5	6.5	8	8	8	8.5	8.5	8.5	8.5	8.5	8.5	8.5	9.5	9.5	9.5	9.5	5	4	4	4	0	1	4	4	4
XXV	Ticuna (Arara)	9	9	6.5	6.5	6.5	8	8	8	8.5	8.5	8.5	8.5	8.5	8.5	8.5	9.5	9.5	9.5	9.5	5	4	4	4	1	0	4	4	4
XXVI	Karitiana	8.5	8.5	8.5	8.5	8.5	7.5	7.5	7.5	6.5	6.5	6.5	6.5	6.5	6.5	6.5	8.5	8.5	8.5	8.5	5.5	2	3	3	4	4	0	3	2
XXVII	Surui	8.5	8.5	8.5	8.5	8.5	7.5	7.5	7.5	6.5	6.5	6.5	6.5	6.5	6.5	6.5	8.5	8.5	8.5	8.5	5.5	3	3	3	4	4	3	0	3
XXVIII	Ache	8.5	8.5	8.5	8.5	8.5	7.5	7.5	7.5	6.5	6.5	6.5	6.5	6.5	6.5	6.5	8.5	8.5	8.5	8.5	5.5	2	3	3	4	4	2	3	0

Each colored block represents the between-stock comparisons for a particular pair of stocks.
