# Supplementary material for: Genetic Variation and Population Structure in Native Americans
Source: PLoS Genet. 2007 Nov 23;3(11):e185. doi: 10.1371/journal.pgen.0030185 (PMC2082466; doi:10.1371/journal.pgen.0030185)
Supplement: Supplementary file 32 [file pgen.0030185.st012.pdf]

Table S12. Correlation of heterozygosity with distance from the Bering Strait when excluding populations with the highest level of European admixture.

Population	Estimated European admixture	Correlation coefficient of heterozygosity with distance from the Bering Strait
Cree	0.2422	-0.368
Arhuaco	0.1844	-0.368
Ojibwa	0.1822	-0.317
Huilliche	0.1640	-0.423
Wayuu	0.1236	-0.434
Kaingang	0.0979	-0.473
Quechua	0.0932	-0.541
Inga	0.0871	-0.551
Maya	0.0828	-0.530
Guarani	0.0827	-0.645

The correlation coefficient on a given row is obtained when excluding from the computation all populations on that row or above it. Populations are sorted in decreasing order of European admixture, estimates of which were obtained from the values plotted in Figure 6.
