# Supplementary material for: Genetic Variation and Population Structure in Native Americans
Source: PLoS Genet. 2007 Nov 23;3(11):e185. doi: 10.1371/journal.pgen.0030185 (PMC2082466; doi:10.1371/journal.pgen.0030185)
Supplement: Supplementary file 33 [file pgen.0030185.st013.pdf]

Table S13. Inferred relative pairs within populations (part I).

Population	Inferred relative pairs	Comments	Individuals excluded from N379	Individuals excluded from N354
Ache	(2735, 2746) PO<br>(2746, 2747) PO<br><br>(2739, 2748) AV, HS, or GG	(2735, 2747) were inferred to be cousins, avuncular, or half sibs. It is likely that 2746 (f) is the parent of 2735 (f) and 2747 (f). No other relationships involving 2735, 2746, 2747.<br>(2736, 2748) are inferred to be cousins. No other relationships involving 2736, 2739, 2748.<br>(2742, 2750), (2749, 2751) are inferred to be cousins.<br>No other relationships in this population.	2746	2746<br>2747<br><br>2748
Arhuaco	(2157, 2158) PO<br>(2577, 2732) PO<br>(2579, 2732) PO<br>(2577, 2579) FS<br>(2732, 2733) FS<br>(2460, 2575) FS<br>(2458, 2459) FS<br>(2458, 2576) FS<br>(2459, 2576) FS<br>(2159, 2420) FS	No other relationships involving 2157, 2158.<br>It is likely that 2732 (f) is the parent of full sibs 2577 (f) and 2579 (f). No other relationships involving 2577, 2579, 2732, 2733.<br><br><br>No other relationships involving 2460, 2575.<br>No other relationships involving 2458, 2459, 2576.<br><br><br>(2159, 2160), (2160, 2420), (2159, 2419), (2160, 2420), (2419, 2420) are inferred to be cousins. No other relationships involving 2159, 2160, 2419, 2420.<br>No other relationships in this population.	2158<br>2577<br>2579<br><br>2733<br>2460<br>2458<br>2576<br><br>2159	2158<br>2577<br>2579<br><br>2733<br>2460<br>2458<br>2576<br><br>2159
Aymara		No relationships in this population.		
Cabecar	(2034, 2039) FS<br>(2021, 2026) AV, HS, or CO<br>(2035, 2037) HS, AV, or GG	No other relationships involving 2034, 2039.<br>No other relationships involving 2021, 2026.<br>(2033, 2035), (2033, 2037), (2037, 2040), (2031, 2037), (2031, 2040), (2031, 2032), (2031, 2038), (2033, 2038) are inferred to be cousins. No other relationships involving 2031, 2032, 2033, 2035, 2037, 2038, 2040.<br>(2023, 2024) are inferred to be cousins.<br>No other relationships in this population.	2034	2034<br>2026<br>2037
