# Supplementary material for: Genetic Variation and Population Structure in Native Americans
Source: PLoS Genet. 2007 Nov 23;3(11):e185. doi: 10.1371/journal.pgen.0030185 (PMC2082466; doi:10.1371/journal.pgen.0030185)
Supplement: Supplementary file 34 [file pgen.0030185.st014.pdf]

Table S14. Inferred relative pairs within populations (part II).

Population	Inferred relative pairs	Comments	Individuals excluded from N379	Individuals excluded from N354
Chipewyan	(2394, 2395) FS	No other non-cousin relationships involving 2390, 2394, 2395, 2474.	2395	2395
	(2390, 2395) GG, HS, CO, or AV			2390
	(2390, 2474) HS, AV, CO, or GG			
	(2392, 2399) PO	It is likely that 2392 (m) is the parent of 2399 (m) and 2400 (m). No other non-cousin relationships involving 2392, 2399, 2400.	2399	2399
	(2392, 2400) PO		2400	2400
	(2399, 2400) FS			
	(2383, 2560) FS	No other non-cousin relationships involving 2156, 2383, 2398, 2560.	2560	2560
	(2156, 2560) HS, AV, or GG			
	(2156, 2383) AV or HS			2156
	(2383, 2398) GG, CO, or HS			2383
	(2012, 2387) GG, HS, or AV	No other non-cousin relationships involving 2012, 2387.		2387
	(2382, 2393) GG or HS	No other non-cousin relationships involving 2382, 2393.		2393
	(2455, 2800) GG or HS	No other non-cousin relationships involving 2455, 2800.		2800
	(2511, 2515) AV, HS, or CO	No other non-cousin relationships involving 2511, 2515.		2515
		This population has 20 inferred cousin relationships involving 22 individuals.		
Cree	(2411, 2418) FS	No other relationships involving 2411, 2418.<br>(2410, 2417) are inferred to be cousins.<br>No other relationships in this population	2418	2418
Embera	(2569, 2570) PO	No other relationships involving 2569, 2570.	2569	2569
	(2564, 2565) FS	No other relationships involving 2564, 2565, 2566.	2564	2564
	(2564, 2566) FS		2566	2566
	(2565, 2566) FS			
	(2561, 2562) AV, HS, or GG	This pair had a low level of loci with 0 alleles shared identical by state, so it is possible that it is a parent/offspring pair with a high number of genotyping errors. No other relationships involving 2561, 2562.<br>(2568, 2574) are inferred to be cousins.<br>No other relationships in this population.	2562	2562
Guarani	(2729, 2731) PO or GG	(2728, 2729) are inferred to be cousins. No other relationships involving 2728, 2729, 2731.	2729	2729
	(2725, 2726) FS	No other relationships involving 2725, 2726.<br>No other relationships in this population.	2726	2726
