# Supplementary material for: Genetic Variation and Population Structure in Native Americans
Source: PLoS Genet. 2007 Nov 23;3(11):e185. doi: 10.1371/journal.pgen.0030185 (PMC2082466; doi:10.1371/journal.pgen.0030185)
Supplement: Supplementary file 35 [file pgen.0030185.st015.pdf]

Table S15. Inferred relative pairs within populations (part III).

Population	Inferred relative pairs	Comments	Individuals excluded from N379	Individuals excluded from N354
Guaymi	(2011, 2014) HS, AV, or GG<br><br>(2002, 2013) AV, HS, or CO	(2014, 2018) are inferred to be cousins. No other relationships involving 2011, 2014, 2018.<br>(2002, 2017) are inferred to be cousins. No other relationships involving 2002, 2013, 2017.<br>(2004, 2009), (2004, 2019), (2009, 2019) are inferred to be cousins.<br>No other relationships in this population.		2014<br><br>2002
Huilliche		No relationships in this population.		
Inga	(2517, 2518) PO<br>(2501, 2505) HS, AV, or GG	No other relationships involving 2517, 2518.<br>(2503, 2505), (2501, 2503) are inferred to be cousins.<br>No other relationships in this population.	2518	2518<br>2505
Kaingang		No relationships in this population.		
Kaqchikel	(2690, 2694) FS	No other relationships in this population.	2690	2690
Kogi	(2466, 2467) FS<br>(2466, 2476) PO<br>(2467, 2476) PO<br>(2470, 2476) FS<br>(2461, 2473) PO<br>(2462, 2473) PO<br>(2464, 2473) PO<br>(2473, 2475) PO<br>(2461, 2462) FS<br>(2461, 2464) FS<br>(2461, 2475) FS<br>(2462, 2464) FS<br>(2462, 2475) FS<br>(2464, 2475) FS<br>(2468, 2473) FS<br>(2472, 2473) GG or CO<br>(2465, 2472) PO	It is likely that 2476 (f) is the parent of 2466 (m) and 2467 (f). (2466, 2470), (2467, 2470) are inferred to be cousins. No other relationships involving 2466, 2467, 2470, 2476.<br><br>It is likely that 2473 (m) is the parent of 2461 (m), 2462 (m), 2464 (m), and 2475 (f).<br><br>It is strange that (2468, 2473) are full sibs but no relationships are observed with 2468 and 2461, 2462, 2464, or 2475. (2465, 2480) are inferred to be cousins. No other relationships involving 2461, 2462, 2464, 2465, 2468, 2472, 2473, 2475, 2480.<br>No other relationships in this population.	2467<br>2476<br><br>2461<br>2462<br>2464<br>2475<br><br>2473<br><br>2472	2467<br>2476<br>2466<br>2461<br>2462<br>2464<br>2475<br><br>2473<br><br>2472
Mixe		(2069, 2073) are inferred to be cousins.<br>No other relationships in this population.		
Mixtec	(2051, 2055) FS<br>(2052, 2054) GG	No other relationships involving 2051, 2055.<br>This pair had a low level of loci with 0 alleles shared identical by state, so it is possible that it is a parent/offspring pair with a high number of genotyping errors. No other relationships involving 2052, 2054.<br>No other relationships in this population.	2055<br>2054	2055<br>2054
