# Supplementary material for: Genetic Variation and Population Structure in Native Americans
Source: PLoS Genet. 2007 Nov 23;3(11):e185. doi: 10.1371/journal.pgen.0030185 (PMC2082466; doi:10.1371/journal.pgen.0030185)
Supplement: Supplementary file 36 [file pgen.0030185.st016.pdf]

Table S16. Inferred relative pairs within populations (part IV).

Population	Inferred relative pairs	Comments	Individuals excluded from N379	Individuals excluded from N354
Ojibwa	(2421, 2425) FS<br>(2421, 2438) PO<br>(2425, 2438) HS, AV, CO, or GG	It is likely that 2421 (f) is the parent of 2438 (f). No other relationships involving 2421, 2425, 2438.	2421	2421<br>2438
	(2429, 2432) PO	No other relationships involving 2429, 2432.	2432	2432
	(2439, 2440) FS	No other relationships involving 2439, 2440.	2440	2440
	(2430, 2431) PO<br>(2430, 2437) FS	(2431, 2437) are inferred to be cousins. It is likely that 2430 (f) is the parent of 2431 (m). No other relationships involving 2430, 2431, 2437.	2430	2430<br>2437
	(2427, 2428) AV or HS	No other relationships involving 2427, 2428.		2428
	(2422, 2436) HS, CO, AV, or GG	No other relationships involving 2422, 2436.		2436
		No other relationships in this population.		
Quechua		No relationships in this population.		
Ticuna (Arara)	(2543, 2545) FS	This pair had a high level of loci with 0 alleles shared identical by state, so it is possible that it is a pair more distant than full sibs. No other relationships involving 2543, 2545.	2543	2543
		(2551, 2559), (2554, 2557), (2557, 2558) are inferred to be cousins.<br>No other relationships in this population.		
Ticuna (Tarapaca)	(2766, 2795) FS	No other non-cousin relationships involving 2766, 2795.	2795	2795
	(2794, 2797) FS	No other relationships involving 2794, 2797.	2797	2797
	(2767, 2768) FS<br>(2767, 2769) PO<br>(2768, 2770) PO<br>(2767, 2770) HS, AV, or GG<br>(2768, 2769) AV or HS	It is likely that 2767 (f) is the parent of 2769 (f), 2768 (f) is the parent of 2770 (m), and 2767 (f) and 2768 (f) are full sibs. Note that 2769 (f) and 2770 (m) are inferred to be cousins.	2768<br>2767	2768<br>2767
	(2761, 2793) FS<br>(2761, 2764) PO<br>(2763, 2764) PO<br>(2761, 2763) GG<br>(2764, 2793) GG, HS, AV, or CO<br>(2792, 2793) PO<br>(2792, 2796) HS, AV, or GG<br>(2763, 2767) GG	It is likely that 2761 (m) is the parent of 2764 (m), who is the parent of 2763 (f). It is also likely that 2793 (f) is the full sib of 2761 (m). No other non-cousin relationships involving 2761, 2763, 2764, 2767, 2768, 2769, 2770, 2792, 2793, 2796.	2793<br>2764	2793<br>2764<br>2761<br>2792
		This population has 11 inferred cousin relationships involving 12 individuals.		
Tundra Nentsi		No relationships in this population.		
