# Supplementary material for: Genetic Variation and Population Structure in Native Americans
Source: PLoS Genet. 2007 Nov 23;3(11):e185. doi: 10.1371/journal.pgen.0030185 (PMC2082466; doi:10.1371/journal.pgen.0030185)
Supplement: Supplementary file 37 [file pgen.0030185.st017.pdf]

Table S17. Inferred relative pairs within populations (part V).

Population	Inferred relative pairs	Comments	Individuals excluded from N379	Individuals excluded from N354
Waunana	(2581, 2584) PO<br>(2583, 2584) HS, AV, or GG	(2581, 2583) are inferred to be cousins, half sibs, or avuncular. No other relationships involving 2581, 2583, 2584.	2584	2584
	(2595, 2597) PO<br>(2596, 2597) PO	It is likely that 2595 (m) and 2596 (f) are the parents of 2597 (f). (2594, 2595) are inferred to be cousins. No other relationships involving 2594, 2595, 2596, 2597.	2597	2597
	(2585, 2589) PO<br>(2585, 2590) PO<br>(2586, 2589) PO<br>(2586, 2590) PO<br>(2587, 2589) PO<br>(2587, 2590) PO<br>(2588, 2589) PO<br>(2588, 2590) PO<br>(2585, 2586) FS<br>(2585, 2587) FS<br>(2585, 2588) FS<br>(2586, 2587) FS<br>(2586, 2588) FS<br>(2587, 2588) FS	It is likely that 2589 (f) and 2590 (m) are the parents of 2585 (m), 2586 (m), 2587 (f), and 2588 (m).	2585<br>2590<br>2586<br>2587<br>2588	2585<br>2590<br>2586<br>2587<br>2588
	(2590, 2599) FS<br>(2599, 2600) PO<br>(2598, 2600) PO	(2590, 2600) are inferred to be cousins. (2585, 2599) are inferred to be cousins or grandparent/grandchild, (2586, 2599) are inferred to be cousins, (2587, 2599) are inferred to be cousins or grandparent/grandchild, (2588, 2599) are inferred to be cousins. (2589, 2598) are inferred to be cousins. It is likely that 2598 (m) and 2599 (f) are the parents of 2600 (f), and that 2599 (f) is the sibling of 2590 (m). No other relationships involving 2585, 2586, 2587, 2588, 2589, 2590, 2598, 2599, 2600.<br>No other relationships in this population.	2600	2599<br>2600
Wayuu	(2525, 2538) FS<br>(2539, 2540) FS	No other relationships involving 2525, 2538.<br>No other relationships involving 2539, 2540.<br>No other relationships in this population	2525<br>2539	2525<br>2539
Zapotec		No relationships in this population.		
Zenu	(2485, 2486) FS<br>(2485, 2487) FS<br>(2486, 2487) FS	No other relationships involving 2485, 2486, 2487.	2485<br>2487	2485<br>2487
	(2493, 2494) AV, HS, or CO	No other relationships involving 2493, 2494.		2494
	(2499, 2500) AV or HS	No other relationships involving 2499, 2500.		2499
	(2483, 2496) GG	(2495, 2496), (2496, 2497), (2495, 2497), (2488, 2495), (2488, 2497) are inferred to be cousins.<br>No other relationships in this population.		2496
