# Supplementary material for: Genetic Variation and Population Structure in Native Americans
Source: PLoS Genet. 2007 Nov 23;3(11):e185. doi: 10.1371/journal.pgen.0030185 (PMC2082466; doi:10.1371/journal.pgen.0030185)
Supplement: Supplementary file 38 [file pgen.0030185.st018.pdf]

Table S18. Number of inferred relative pairs within populations.

**Population**	**Sample size in N436**	**Number of PO pairs**	**Number of FS pairs**	**Number of second-degree pairs**
Ache	19	2	0	1
Arhuaco	17	3	7	0
Aymara	18	0	0	0
Cabecar	20	0	1	2
Chipewyan	29	2	3	9
Cree	18	0	1	0
Embera	11	1	3	1
Guarani	10	1	1	0
Guaymi	18	0	0	2
Huilliche	20	0	0	0
Inga	17	1	0	1
Kaingang	7	0	0	0
Kaqchikel	12	0	1	0
Kogi	17	7	9	1
Mixe	20	0	0	0
Mixtec	20	0	1	1
Ojibwa	20	3	3	3
Quechua	20	0	0	0
Ticuna (Arara)	17	0	1	0
Ticuna (Tarapaca)	18	5	4	6
Tundra Nentsi	14	0	0	0
Waunana	20	13	7	1
Wayuu	17	0	2	0
Zapotec	19	0	0	0
Zenu	18	0	3	3
**Total**	**436**	**38**	**47**	**31**
