# Supplementary material for: Genetic Variation and Population Structure in Native Americans
Source: PLoS Genet. 2007 Nov 23;3(11):e185. doi: 10.1371/journal.pgen.0030185 (PMC2082466; doi:10.1371/journal.pgen.0030185)
Supplement: Supplementary file 39 [file pgen.0030185.st019.pdf]

Table S19. The 38 inferred parent/offspring pairs in N436.

Population	First individual			Second individual		
	Identification number	Sex	Parent (P), offspring (O), or uncertain (U)	Identification number	Sex	Parent (P), offspring (O), or uncertain (U)
Ache	2735	F	O	2746	F	P
Ache	2746	F	P	2747	F	O
Arhuaco	2157	F	U	2158	M	U
Arhuaco	2577	F	O	2732	F	P
Arhuaco	2579	F	O	2732	F	P
Chipewyan	2392	M	P	2399	M	O
Chipewyan	2392	M	P	2400	M	O
Embera	2569	F	U	2570	F	U
Guarani	2729	F	U	2731	F	U
Inga	2517	F	U	2518	F	U
Kogi	2461	M	O	2473	M	P
Kogi	2462	M	O	2473	M	P
Kogi	2464	M	O	2473	M	P
Kogi	2465	F	U	2472	M	U
Kogi	2466	M	O	2476	F	P
Kogi	2467	F	O	2476	F	P
Kogi	2473	M	P	2475	F	O
Ojibwa	2421	F	P	2438	F	O
Ojibwa	2429	M	U	2432	M	U
Ojibwa	2430	F	P	2431	M	O
Ticuna (Tarapaca)	2761	M	P	2764	M	O
Ticuna (Tarapaca)	2763	F	O	2764	M	P
Ticuna (Tarapaca)	2767	F	P	2769	F	O
Ticuna (Tarapaca)	2768	F	P	2770	M	O
Ticuna (Tarapaca)	2792	M	U	2793	F	U
Waunana	2581	M	U	2584	M	U
Waunana	2585	M	O	2589	F	P
Waunana	2585	M	O	2590	M	P
Waunana	2586	M	O	2589	F	P
Waunana	2586	M	O	2590	M	P
Waunana	2587	F	O	2589	F	P
Waunana	2587	F	O	2590	M	P
Waunana	2588	M	O	2589	F	P
Waunana	2588	M	O	2590	M	P
Waunana	2595	M	P	2597	F	O
Waunana	2596	F	P	2597	F	O
Waunana	2598	M	P	2600	F	O
Waunana	2599	F	P	2600	F	O
