# Supplementary material for: Genetic Variation and Population Structure in Native Americans
Source: PLoS Genet. 2007 Nov 23;3(11):e185. doi: 10.1371/journal.pgen.0030185 (PMC2082466; doi:10.1371/journal.pgen.0030185)
Supplement: Supplementary file 40 [file pgen.0030185.st020.pdf]

Table S20. The 47 inferred full sib pairs in N436.

Population	First individual		Second individual	
	Identification number	Sex	Identification number	Sex
Arhuaco	2159	F	2420	M
Arhuaco	2458	F	2459	M
Arhuaco	2458	F	2576	F
Arhuaco	2459	M	2576	F
Arhuaco	2460	M	2575	M
Arhuaco	2577	F	2579	F
Arhuaco	2732	F	2733	F
Cabecar	2034	M	2039	F
Chipewyan	2383	M	2560	M
Chipewyan	2394	F	2395	F
Chipewyan	2399	M	2400	M
Cree	2411	M	2418	F
Embera	2564	M	2565	F
Embera	2564	M	2566	F
Embera	2565	F	2566	F
Guarani	2725	F	2726	M
Kaqchikel	2690	M	2694	M
Kogi	2461	M	2462	M
Kogi	2461	M	2464	M
Kogi	2461	M	2475	F
Kogi	2462	M	2464	M
Kogi	2462	M	2475	F
Kogi	2464	M	2475	F
Kogi	2466	M	2467	F
Kogi	2468	F	2473	M
Kogi	2470	F	2476	F
Mixtec	2051	M	2055	Ambiguous
Ojibwa	2421	F	2425	F
Ojibwa	2430	F	2437	F
Ojibwa	2439	F	2440	M
Ticuna (Arara)	2543	F	2545	F
Ticuna (Tarapaca)	2761	M	2793	F
Ticuna (Tarapaca)	2766	F	2795	M
Ticuna (Tarapaca)	2767	F	2768	F
Ticuna (Tarapaca)	2794	M	2797	F
Waunana	2585	M	2586	M
Waunana	2585	M	2587	F
Waunana	2585	M	2588	M
Waunana	2586	M	2587	F
Waunana	2586	M	2588	M
Waunana	2587	F	2588	M
Waunana	2590	M	2599	F
Wayuu	2525	M	2538	M
Wayuu	2539	M	2540	M
Zenu	2485	M	2486	M
Zenu	2485	M	2487	M
Zenu	2486	M	2487	M
