# Supplementary material for: Genetic Variation and Population Structure in Native Americans
Source: PLoS Genet. 2007 Nov 23;3(11):e185. doi: 10.1371/journal.pgen.0030185 (PMC2082466; doi:10.1371/journal.pgen.0030185)
Supplement: Supplementary file 41 [file pgen.0030185.st021.pdf]

**Table S21**. The 31 inferred within-population second-degree relative pairs in N436.

| Population | First individual | | | Second individual | | |
|---|---|---|---|---|---|---|
| | Identification number | Sex | Half sib (H), aunt or uncle (A), niece or nephew (N), grandparent (G), grandchild (C), or uncertain (U) | Identification number | Sex | Half sib (H), aunt or uncle (A), niece or nephew (N), grandparent (G), grandchild (C), or uncertain (U) |
| Ache | 2739 | F | U | 2748 | M | U |
| Cabecar | 2021 | M | U | 2026 | F | U |
| Cabecar | 2035 | M | U | 2037 | F | U |
| Chipewyan | 2012 | M | U | 2387 | M | U |
| Chipewyan | 2156 | M | U | 2383 | M | U |
| Chipewyan | 2156 | M | U | 2560 | M | U |
| Chipewyan | 2382 | M | U | 2393 | F | U |
| Chipewyan | 2383 | M | U | 2398 | M | U |
| Chipewyan | 2390 | M | U | 2395 | F | U |
| Chipewyan | 2390 | M | U | 2474 | M | U |
| Chipewyan | 2455 | M | U | 2800 | M | U |
| Chipewyan | 2511 | M | U | 2515 | M | U |
| Embera | 2561 | M | U | 2562 | F | U |
| Guaymi | 2002 | M | U | 2013 | F | U |
| Guaymi | 2011 | F | U | 2014 | F | U |
| Inga | 2501 | F | U | 2505 | M | U |
| Kogi | 2472 | M | U | 2473 | M | U |
| Mixtec | 2052 | F | U | 2054 | F | U |
| Ojibwa | 2422 | F | U | 2436 | F | U |
| Ojibwa | 2425 | F | A | 2438 | F | N |
| Ojibwa | 2427 | F | U | 2428 | M | U |
| Ticuna (Tarapaca) | 2761 | M | G | 2763 | F | C |
| Ticuna (Tarapaca) | 2763 | F | U | 2767 | F | U |
| Ticuna (Tarapaca) | 2764 | M | N | 2793 | F | A |
| Ticuna (Tarapaca) | 2767 | F | A | 2770 | M | N |
| Ticuna (Tarapaca) | 2768 | F | A | 2769 | F | N |
| Ticuna (Tarapaca) | 2792 | M | U | 2796 | M | U |
| Waunana | 2583 | F | U | 2584 | M | U |
| Zenu | 2483 | M | U | 2496 | F | U |
| Zenu | 2493 | F | U | 2494 | F | U |
| Zenu | 2499 | M | U | 2500 | M | U |

The type of second-degree relationship is regarded as certain only if a single type of relationship is compatible with the pairs that appear in Tables S19 and S20 (and with the lack of appearance of any other pairs in those tables). Some pairs listed with uncertain relationship might not actually be second-degree relatives, and some second-degree pairs might not have been identified. There is some possibility that (2052, 2054) and (2561, 2562) may be parent/offspring pairs.
