# Supplementary material for: Genetic Variation and Population Structure in Native Americans
Source: PLoS Genet. 2007 Nov 23;3(11):e185. doi: 10.1371/journal.pgen.0030185 (PMC2082466; doi:10.1371/journal.pgen.0030185)
Supplement: Supplementary file 42 [file pgen.0030185.st022.pdf]

Table S22. The five inferred parent/parent/offspring trios in N436.

Population	Identification number of father	Identification number of mother	Identification number of offspring	Sex of offspring
Waunana	2595	2596	2597	F
Waunana	2590	2589	2585	M
Waunana	2590	2589	2586	M
Waunana	2590	2589	2587	F
Waunana	2590	2589	2588	M
