# Supplementary material for: Genetic Variation and Population Structure in Native Americans
Source: PLoS Genet. 2007 Nov 23;3(11):e185. doi: 10.1371/journal.pgen.0030185 (PMC2082466; doi:10.1371/journal.pgen.0030185)
Supplement: Supplementary file 43 [file pgen.0030185.st023.pdf]

Table S23. Numbers of individuals excluded from N436 in N379 and N354.

Population	Sample size in N436	Number of individuals excluded from N436 in N379	Sample size in N379	Number of individuals excluded from N436 in N354	Sample size in N354
Ache	19	1	18	3	16
Arhuaco	17	8	9	8	9
Aymara	18	0	18	0	18
Cabecar	20	1	19	3	17
Chipewyan	29	4	25	11	18
Cree	18	1	17	1	17
Embera	11	4	7	4	7
Guarani	10	2	8	2	8
Guaymi	18	0	18	2	16
Huilliche	20	0	20	0	20
Inga	17	1	16	2	15
Kaingang	7	0	7	0	7
Kaqchikel	12	1	11	1	11
Kogi	17	9	8	10	7
Mixe	20	0	20	0	20
Mixtec	20	2	18	2	18
Ojibwa	20	4	16	8	12
Quechua	20	0	20	0	20
Ticuna (Arara)	17	1	16	1	16
Ticuna (Tarapaca)	18	6	12	8	10
Tundra Nentsi	14	0	14	0	14
Waunana	20	8	12	9	11
Wayuu	17	2	15	2	15
Zapotec	19	0	19	0	19
Zenu	18	2	16	5	13
**Total**	**436**	**57**	**379**	**82**	**354**

Kogi individual 2463 was excluded from N379 and N355 despite not being part of any inferred relative pairs in the Kogi population. When searching for relative pairs in a joint analysis of the Arhuaco and Kogi data, this individual was inferred to be part of second-degree pairs with Arhuaco individuals 2159, 2160, 2419, and 2420. It is likely that this individual is from the Arhuaco population but was mislabeled.
