# Supplementary material for: Genetic Variation and Population Structure in Native Americans
Source: PLoS Genet. 2007 Nov 23;3(11):e185. doi: 10.1371/journal.pgen.0030185 (PMC2082466; doi:10.1371/journal.pgen.0030185)
Supplement: Supplementary file 44 [file pgen.0030185.st024.pdf]

Table S24. The 57 individuals included in N436 but not in N379.

Population	Individuals in N436 but not in N379
Cabecar	2034
Mixtec	2054
Mixtec	2055
Arhuaco	2158
Arhuaco	2159
Chipewyan	2395
Chipewyan	2399
Chipewyan	2400
Cree	2418
Ojibwa	2421
Ojibwa	2430
Ojibwa	2432
Ojibwa	2440
Arhuaco	2458
Arhuaco	2460
Kogi	2461
Kogi	2462
Kogi	2463
Kogi	2464
Kogi	2467
Kogi	2472
Kogi	2473
Kogi	2475
Kogi	2476
Zenu	2485
Zenu	2487

Population	Individuals in N436 but not in N379 (continued)
Inga	2518
Wayuu	2525
Wayuu	2539
Ticuna (Arara)	2543
Chipewyan	2560
Embera	2562
Embera	2564
Embera	2566
Embera	2569
Arhuaco	2576
Arhuaco	2577
Arhuaco	2579
Waunana	2584
Waunana	2585
Waunana	2586
Waunana	2587
Waunana	2588
Waunana	2590
Waunana	2597
Waunana	2600
Kaqchikel	2690
Guarani	2726
Guarani	2729
Arhuaco	2733
Ache	2746
Ticuna (Tarapaca)	2764
Ticuna (Tarapaca)	2767
Ticuna (Tarapaca)	2768
Ticuna (Tarapaca)	2793
Ticuna (Tarapaca)	2795
Ticuna (Tarapaca)	2797
