# Supplementary material for: Genetic Variation and Population Structure in Native Americans
Source: PLoS Genet. 2007 Nov 23;3(11):e185. doi: 10.1371/journal.pgen.0030185 (PMC2082466; doi:10.1371/journal.pgen.0030185)
Supplement: Supplementary file 45 [file pgen.0030185.st025.pdf]

Table S25. The 82 individuals included in N436 but not in N354.

Population	Individuals in N436 but not in N354
Guaymi	2002
Guaymi	2014
Cabecar	2026
Cabecar	2034
Cabecar	2037
Mixtec	2054
Mixtec	2055
Chipewyan	2156
Arhuaco	2158
Arhuaco	2159
Chipewyan	2383
Chipewyan	2387
Chipewyan	2390
Chipewyan	2393
Chipewyan	2395
Chipewyan	2399
Chipewyan	2400
Cree	2418
Ojibwa	2421
Ojibwa	2428
Ojibwa	2430
Ojibwa	2432
Ojibwa	2436
Ojibwa	2437
Ojibwa	2438
Ojibwa	2440
Arhuaco	2458
Arhuaco	2460
Kogi	2461
Kogi	2462
Kogi	2463
Kogi	2464
Kogi	2466
Kogi	2467
Kogi	2472
Kogi	2473
Kogi	2475
Kogi	2476
Zenu	2485
Zenu	2487
Zenu	2494
**Population**	**Individuals in N436 but not in N354 (continued)**
Zenu	2496
Zenu	2499
Inga	2505
Chipewyan	2515
Inga	2518
Wayuu	2525
Wayuu	2539
Ticuna (Arara)	2543
Chipewyan	2560
Embera	2562
Embera	2564
Embera	2566
Embera	2569
Arhuaco	2576
Arhuaco	2577
Arhuaco	2579
Waunana	2584
Waunana	2585
Waunana	2586
Waunana	2587
Waunana	2588
Waunana	2590
Waunana	2597
Waunana	2599
Waunana	2600
Kaqchikel	2690
Guarani	2726
Guarani	2729
Arhuaco	2733
Ache	2746
Ache	2747
Ache	2748
Ticuna (Tarapaca)	2761
Ticuna (Tarapaca)	2764
Ticuna (Tarapaca)	2767
Ticuna (Tarapaca)	2768
Ticuna (Tarapaca)	2792
Ticuna (Tarapaca)	2793
Ticuna (Tarapaca)	2795
Ticuna (Tarapaca)	2797
Chipewyan	2800
