# Supplementary material for: Genetic Variation and Population Structure in Native Americans
Source: PLoS Genet. 2007 Nov 23;3(11):e185. doi: 10.1371/journal.pgen.0030185 (PMC2082466; doi:10.1371/journal.pgen.0030185)
Supplement: Supplementary file 46 [file pgen.0030185.st026.pdf]

Table S26. The missing data rate in N436, N379, and N354.

Population	Missing data rate in N436 (%)	Missing data rate in N379 (%)	Missing data rate in N354 (%)
Ache	11.4	11.5	12.2
Arhuaco	3.5	1.5	1.5
Aymara	2.8	2.8	2.8
Cabecar	1.9	1.9	1.9
Chipewyan	6.1	5.9	6.2
Cree	3.9	4.1	4.1
Embera	4.6	1.3	1.3
Guarani	4.1	4.1	4.1
Guaymi	7.3	7.3	7.9
Huilliche	6.7	6.7	6.7
Inga	4.3	4.5	4.7
Kaingang	14.5	14.5	14.5
Kaqchikel	0.8	0.8	0.8
Kogi	5.1	2.0	2.2
Mixe	3.5	3.5	3.5
Mixtec	5.9	5.5	5.5
Ojibwa	2.7	2.0	1.7
Quechua	2.4	2.4	2.4
Ticuna (Arara)	13.8	13.3	13.3
Ticuna (Tarapaca)	2.4	1.8	1.8
Tundra Nentsi	7.7	7.7	7.7
Waunana	0.8	0.7	0.8
Wayuu	3.8	4.0	4.0
Zapotec	13.4	13.4	13.4
Zenu	0.9	0.9	0.8
**Whole data set**	**5.2**	**5.2**	**5.3**
