# Supplementary material for: Genetic Variation and Population Structure in Native Americans
Source: PLoS Genet. 2007 Nov 23;3(11):e185. doi: 10.1371/journal.pgen.0030185 (PMC2082466; doi:10.1371/journal.pgen.0030185)
Supplement: Supplementary file 47 [file pgen.0030185.st027.pdf]

Table S27. Nei genetic distance matrix for Native American populations.

		I	II	III	IV	V	VI	VII	VIII	IX	X	XI	XII	XIII	XIV	XV	XVI	XVII	XVIII	XIX	XX	XXI	XXII	XXIII	XXIV	XXV	XXVI	XXVII	XXVIII	XXIX
I	Chipewyan	0.000000	0.089093	0.092227	0.144617	0.118662	0.109106	0.119636	0.095235	0.115326	0.134199	0.170033	0.175460	0.161309	0.145371	0.161422	0.125910	0.119494	0.097534	0.107015	0.108237	0.188339	0.141623	0.109815	0.154039	0.163361	0.168231	0.174620	0.228545	0.242337
II	Cree	0.089093	0.000000	0.077204	0.138022	0.108005	0.099785	0.106755	0.082107	0.109767	0.128482	0.158232	0.174266	0.152171	0.134764	0.152755	0.119287	0.113189	0.086827	0.098746	0.098787	0.184386	0.131343	0.104457	0.149349	0.156987	0.160205	0.168875	0.220003	0.231867
III	Ojibwa	0.092227	0.077204	0.000000	0.139126	0.108726	0.100964	0.108893	0.083392	0.105878	0.125713	0.154582	0.171231	0.149725	0.133931	0.152921	0.114084	0.111068	0.089314	0.099526	0.100057	0.183102	0.135606	0.103364	0.147322	0.159753	0.163326	0.170547	0.221728	0.237129
IV	Pima	0.144617	0.138022	0.139126	0.000000	0.125751	0.117969	0.121676	0.110283	0.120875	0.144852	0.168433	0.174689	0.176895	0.151814	0.167530	0.135598	0.133208	0.114167	0.115753	0.128772	0.199058	0.157443	0.132105	0.156347	0.166938	0.171569	0.179323	0.214531	0.245685
V	Mixtec	0.118662	0.108005	0.108726	0.125751	0.000000	0.082231	0.079591	0.076612	0.088492	0.110169	0.136008	0.146112	0.141317	0.120905	0.137934	0.102257	0.100243	0.082306	0.084806	0.094323	0.171856	0.125572	0.094953	0.126910	0.136330	0.136346	0.147896	0.194344	0.208499
VI	Zapotec	0.109106	0.099785	0.100964	0.117969	0.082231	0.000000	0.080801	0.071028	0.083026	0.107699	0.133845	0.143187	0.140241	0.119361	0.134125	0.097290	0.093542	0.077157	0.079754	0.086185	0.166180	0.119261	0.089154	0.125560	0.134282	0.135988	0.145948	0.195305	0.204017
VII	Mixe	0.119636	0.106755	0.108893	0.121676	0.079591	0.080801	0.000000	0.074458	0.086243	0.109921	0.130080	0.140198	0.142759	0.115885	0.137813	0.097511	0.096916	0.076896	0.081649	0.091943	0.168769	0.121314	0.094490	0.126702	0.129842	0.136056	0.142690	0.191792	0.208825
VIII	Maya	0.095235	0.082107	0.083392	0.110283	0.076612	0.071028	0.074458	0.000000	0.070214	0.094738	0.119140	0.132465	0.125163	0.104082	0.121181	0.085274	0.082063	0.058946	0.064747	0.074440	0.154046	0.106629	0.078154	0.111865	0.123229	0.128421	0.131489	0.182361	0.197897
IX	Kaqchikel	0.115326	0.109767	0.105878	0.120875	0.088492	0.083026	0.086243	0.070214	0.000000	0.102419	0.124628	0.136498	0.135531	0.113928	0.132986	0.096187	0.091518	0.073358	0.078249	0.090071	0.168450	0.117369	0.092977	0.121704	0.127883	0.133990	0.142888	0.189392	0.199457
X	Cabecar	0.134199	0.128482	0.125713	0.144852	0.110169	0.107699	0.109921	0.094738	0.102419	0.000000	0.104089	0.138907	0.141294	0.116062	0.132758	0.104695	0.114105	0.098539	0.100224	0.110618	0.184519	0.135782	0.102319	0.142613	0.151700	0.158309	0.157623	0.203858	0.225386
XI	Guaymi	0.170033	0.158232	0.154582	0.168433	0.136008	0.133845	0.130080	0.119140	0.124628	0.104089	0.000000	0.148283	0.165949	0.137935	0.160036	0.127262	0.138469	0.124104	0.126095	0.135149	0.200278	0.160677	0.128534	0.164732	0.168495	0.173219	0.175528	0.219277	0.232057
XII	Kogi	0.175460	0.174266	0.171231	0.174689	0.146112	0.143187	0.140198	0.132465	0.136498	0.138907	0.148283	0.000000	0.138510	0.154744	0.173287	0.142517	0.150852	0.137332	0.137363	0.150724	0.205352	0.170937	0.138976	0.168656	0.170799	0.175526	0.178645	0.230229	0.241140
XIII	Arhuaco	0.161309	0.152171	0.149725	0.176895	0.141317	0.140241	0.142759	0.125163	0.135531	0.141294	0.165949	0.138510	0.000000	0.156180	0.181553	0.138208	0.142599	0.128061	0.132348	0.141984	0.214933	0.169897	0.130262	0.173719	0.173237	0.182438	0.188305	0.233464	0.251531
XIV	Waunana	0.145371	0.134764	0.133931	0.151814	0.120905	0.119361	0.115885	0.104082	0.113928	0.116062	0.137935	0.154744	0.156180	0.000000	0.137888	0.118540	0.119465	0.103364	0.105187	0.117776	0.189614	0.142772	0.113828	0.145930	0.153066	0.160477	0.164320	0.206780	0.220673
XV	Embera	0.161422	0.152755	0.152921	0.167530	0.137934	0.134125	0.137813	0.121181	0.132986	0.132758	0.160036	0.173287	0.181553	0.137888	0.000000	0.135211	0.136101	0.124433	0.127233	0.134004	0.202755	0.165714	0.133005	0.166163	0.166662	0.174713	0.179713	0.228149	0.240966
XVI	Zenu	0.125910	0.119287	0.114084	0.135598	0.102257	0.097290	0.097511	0.085274	0.096187	0.104695	0.127262	0.142517	0.138208	0.118540	0.135211	0.000000	0.103633	0.085665	0.091666	0.099496	0.171933	0.128772	0.095252	0.130465	0.141761	0.146873	0.148507	0.199268	0.207078
XVII	Inga	0.119494	0.113189	0.111068	0.133208	0.100243	0.093542	0.096916	0.082063	0.091518	0.114105	0.138469	0.150852	0.142599	0.119465	0.136101	0.103633	0.000000	0.078017	0.084069	0.096563	0.170635	0.119535	0.097175	0.124928	0.125449	0.137025	0.144327	0.192332	0.198911
XVIII	Quechua	0.097534	0.086827	0.089314	0.114167	0.082306	0.077157	0.076896	0.058946	0.073358	0.098539	0.124104	0.137332	0.128061	0.103364	0.124433	0.085665	0.078017	0.000000	0.055719	0.073958	0.157477	0.103704	0.079444	0.111564	0.114327	0.122414	0.131953	0.181312	0.193557
XIX	Aymara	0.107015	0.098746	0.099526	0.115753	0.084806	0.079754	0.081649	0.064747	0.078249	0.100224	0.126095	0.137363	0.132348	0.105187	0.127233	0.091666	0.084069	0.055719	0.000000	0.080125	0.161091	0.112458	0.085244	0.117009	0.122520	0.127771	0.138710	0.181220	0.201788
XX	Huilliche	0.108237	0.098787	0.100057	0.128772	0.094323	0.086185	0.091943	0.074440	0.090071	0.110618	0.135149	0.150724	0.141984	0.117776	0.134004	0.099496	0.096563	0.073958	0.080125	0.000000	0.164649	0.118998	0.090353	0.126975	0.136804	0.140732	0.144919	0.197740	0.203268
XXI	Kaingang	0.188339	0.184386	0.183102	0.199058	0.171856	0.166180	0.168769	0.154046	0.168450	0.184519	0.200278	0.205352	0.214933	0.189614	0.202755	0.171933	0.170635	0.157477	0.161091	0.164649	0.000000	0.185986	0.161865	0.187230	0.188788	0.203887	0.201988	0.250664	0.247657
XXII	Guarani	0.141623	0.131343	0.135606	0.157443	0.125572	0.119261	0.121314	0.106629	0.117369	0.135782	0.160677	0.170937	0.169897	0.142772	0.165714	0.128772	0.119535	0.103704	0.112458	0.118998	0.185986	0.000000	0.120419	0.146915	0.155942	0.157718	0.160272	0.201686	0.211214
XXIII	Wayuu	0.109815	0.104457	0.103364	0.132105	0.094953	0.089154	0.094490	0.078154	0.092977	0.102319	0.128534	0.138976	0.130262	0.113828	0.133005	0.095252	0.097175	0.079444	0.085244	0.090353	0.161865	0.120419	0.000000	0.128429	0.131554	0.140945	0.147418	0.197157	0.208705
XXIV	Piapoco	0.154039	0.149349	0.147322	0.156347	0.126910	0.125560	0.126702	0.111865	0.121704	0.142613	0.164732	0.168656	0.173719	0.145930	0.166163	0.130465	0.124928	0.111564	0.117009	0.126975	0.187230	0.146915	0.128429	0.000000	0.143895	0.152271	0.155187	0.205607	0.216941
XXV	Ticuna (Tarapaca)	0.163361	0.156987	0.159753	0.166938	0.136330	0.134282	0.129842	0.123229	0.127883	0.151700	0.168495	0.170799	0.173237	0.153066	0.166662	0.141761	0.125449	0.114327	0.122520	0.136804	0.188788	0.155942	0.131554	0.143895	0.000000	0.111751	0.162722	0.210387	0.220788
XXVI	Ticuna (Arara)	0.168231	0.160205	0.163326	0.171569	0.136346	0.135988	0.136056	0.128421	0.133990	0.158309	0.173219	0.175526	0.182438	0.160477	0.174713	0.146873	0.137025	0.122414	0.127771	0.140732	0.203887	0.157718	0.140945	0.152271	0.111751	0.000000	0.177869	0.216289	0.229809
XXVII	Karitiana	0.174620	0.168875	0.170547	0.179323	0.147896	0.145948	0.142690	0.131489	0.142888	0.157623	0.175528	0.178645	0.188305	0.164320	0.179713	0.148507	0.144327	0.131953	0.138710	0.144919	0.201988	0.160272	0.147418	0.155187	0.162722	0.177869	0.000000	0.214431	0.232908
XXVIII	Surui	0.228545	0.220003	0.221728	0.214531	0.194344	0.195305	0.191792	0.182361	0.189392	0.203858	0.219277	0.230229	0.233464	0.206780	0.228149	0.199268	0.192332	0.181312	0.181220	0.197740	0.250664	0.201686	0.197157	0.205607	0.210387	0.216289	0.214431	0.000000	0.269883
XXIX	Ache	0.242337	0.231867	0.237129	0.245685	0.208499	0.204017	0.208825	0.197897	0.199457	0.225386	0.232057	0.241140	0.251531	0.220673	0.240966	0.207078	0.198911	0.193557	0.201788	0.203268	0.247657	0.211214	0.208705	0.216941	0.220788	0.229809	0.232908	0.269883	0.000000
