# Supplementary material for: Genetic Variation and Population Structure in Native Americans
Source: PLoS Genet. 2007 Nov 23;3(11):e185. doi: 10.1371/journal.pgen.0030185 (PMC2082466; doi:10.1371/journal.pgen.0030185)
Supplement: Supplementary file 48 [file pgen.0030185.st028.pdf]

Table S28. Reynolds genetic distance matrix for Native American populations.

		I	II	III	IV	V	VI	VII	VIII	IX	X	XI	XII	XIII	XIV	XV	XVI	XVII	XVIII	XIX	XX	XXI	XXII	XXIII	XXIV	XXV	XXVI	XXVII	XXVIII	XXIX
I	Chipewyan	0.000000	0.059269	0.061301	0.106976	0.082840	0.071899	0.086337	0.065151	0.076471	0.102778	0.132740	0.137458	0.115473	0.106914	0.117111	0.089522	0.086773	0.067144	0.075030	0.074469	0.132014	0.097329	0.075862	0.110321	0.125726	0.129276	0.136513	0.186265	0.193330
II	Cree	0.059269	0.000000	0.045781	0.095774	0.068872	0.060010	0.070257	0.051109	0.067839	0.091722	0.116533	0.131019	0.104091	0.094528	0.104089	0.078339	0.076024	0.055283	0.061978	0.062724	0.124311	0.083427	0.064925	0.100466	0.114931	0.114683	0.125985	0.169458	0.178319
III	Ojibwa	0.061301	0.045781	0.000000	0.099447	0.072496	0.062119	0.073063	0.053229	0.065264	0.090687	0.114465	0.128894	0.103130	0.095154	0.104318	0.075964	0.076407	0.057958	0.066528	0.065860	0.124288	0.087849	0.066329	0.100428	0.119482	0.117823	0.128860	0.172977	0.184378
IV	Pima	0.106976	0.095774	0.099447	0.000000	0.096884	0.085047	0.097247	0.079373	0.088197	0.118821	0.145628	0.151550	0.138657	0.124397	0.132746	0.105247	0.104510	0.085317	0.086007	0.095323	0.153304	0.120011	0.095853	0.128092	0.144225	0.146178	0.154633	0.191110	0.216433
V	Mixtec	0.082840	0.068872	0.072496	0.096884	0.000000	0.050687	0.058492	0.047714	0.059181	0.085525	0.112777	0.121060	0.105011	0.091829	0.100000	0.071463	0.071745	0.055033	0.057880	0.062968	0.123748	0.083918	0.062683	0.097240	0.112465	0.109831	0.119573	0.164825	0.176121
VI	Zapotec	0.071899	0.060010	0.062119	0.085047	0.050687	0.000000	0.052761	0.040132	0.047767	0.079669	0.104941	0.113591	0.098349	0.084636	0.093455	0.064196	0.061907	0.045807	0.049251	0.054200	0.114591	0.075433	0.054899	0.089271	0.102664	0.103080	0.112624	0.160643	0.166150
VII	Mixe	0.086337	0.070257	0.073063	0.097247	0.058492	0.052761	0.000000	0.047854	0.058726	0.088503	0.108123	0.119383	0.106956	0.090239	0.104247	0.072775	0.070817	0.051714	0.054744	0.063498	0.122041	0.084530	0.064529	0.099444	0.108557	0.110001	0.120581	0.166425	0.180306
VIII	Maya	0.065151	0.051109	0.053229	0.079373	0.047714	0.040132	0.047854	0.000000	0.039845	0.068177	0.091003	0.103115	0.088160	0.071892	0.081668	0.053778	0.053846	0.033813	0.038048	0.045293	0.102827	0.065398	0.046032	0.076760	0.095510	0.094622	0.099039	0.145336	0.157583
IX	Kaqchikel	0.076471	0.067839	0.065264	0.088197	0.059181	0.047767	0.058726	0.039845	0.000000	0.074283	0.099315	0.109964	0.096577	0.080254	0.091609	0.062857	0.060740	0.043144	0.047254	0.055131	0.116198	0.076494	0.057038	0.084250	0.099073	0.102185	0.111190	0.158815	0.165477
X	Cabecar	0.102778	0.091722	0.090687	0.118821	0.085525	0.079669	0.088503	0.068177	0.074283	0.000000	0.090457	0.121920	0.108586	0.092268	0.103490	0.081837	0.087288	0.072506	0.074708	0.080766	0.140551	0.102038	0.071780	0.112333	0.129046	0.131545	0.133625	0.181968	0.197354
XI	Guaymi	0.132740	0.116533	0.114465	0.145628	0.112777	0.104941	0.108123	0.091003	0.099315	0.090457	0.000000	0.141631	0.136254	0.116016	0.131558	0.105186	0.112150	0.096460	0.099987	0.105935	0.160109	0.126726	0.098967	0.137862	0.151019	0.152282	0.159341	0.207310	0.219675
XII	Kogi	0.137458	0.131019	0.128894	0.151550	0.121060	0.113591	0.119383	0.103115	0.109964	0.121920	0.141631	0.000000	0.123248	0.136726	0.144416	0.118133	0.124357	0.108512	0.110657	0.119139	0.170458	0.142506	0.108035	0.144690	0.156653	0.157957	0.165076	0.220948	0.228489
XIII	Arhuaco	0.115473	0.104091	0.103130	0.138657	0.105011	0.098349	0.106956	0.088160	0.096577	0.108586	0.136254	0.123248	0.000000	0.121312	0.135789	0.103656	0.107178	0.091086	0.095075	0.102918	0.162654	0.123804	0.090087	0.133754	0.141959	0.146717	0.153646	0.198082	0.220502
XIV	Waunana	0.106914	0.094528	0.095154	0.124397	0.091829	0.084636	0.090239	0.071892	0.080254	0.092268	0.116016	0.136726	0.121312	0.000000	0.106697	0.091854	0.091959	0.073733	0.076211	0.085815	0.143931	0.104836	0.078971	0.116077	0.128265	0.134757	0.141420	0.183842	0.193093
XV	Embera	0.117111	0.104089	0.104318	0.132746	0.100000	0.093455	0.104247	0.081668	0.091609	0.103490	0.131558	0.144416	0.135789	0.106697	0.000000	0.098984	0.100870	0.087415	0.090398	0.092995	0.150461	0.120183	0.092406	0.126301	0.138579	0.145801	0.150893	0.200435	0.209300
XVI	Zenu	0.089522	0.078339	0.075964	0.105247	0.071463	0.064196	0.072775	0.053778	0.062857	0.081837	0.105186	0.118133	0.103656	0.091854	0.098984	0.000000	0.071955	0.056163	0.062032	0.067013	0.123087	0.089472	0.061299	0.096558	0.113896	0.112479	0.119369	0.169388	0.176929
XVII	Inga	0.086773	0.076024	0.076407	0.104510	0.071745	0.061907	0.070817	0.053846	0.060740	0.087288	0.112150	0.124357	0.107178	0.091959	0.100870	0.071955	0.000000	0.050399	0.057059	0.068178	0.123546	0.080429	0.064456	0.092527	0.099760	0.108760	0.116566	0.165930	0.168471
XVIII	Quechua	0.067144	0.055283	0.057958	0.085317	0.055033	0.045807	0.051714	0.033813	0.043144	0.072506	0.096460	0.108512	0.091086	0.073733	0.087415	0.056163	0.050399	0.000000	0.030782	0.046863	0.107588	0.064798	0.048710	0.077769	0.086344	0.091566	0.100828	0.147883	0.156493
XIX	Aymara	0.075030	0.061978	0.066528	0.086007	0.057880	0.049251	0.054744	0.038048	0.047254	0.074708	0.099987	0.110657	0.095075	0.076211	0.090398	0.062032	0.057059	0.030782	0.000000	0.052397	0.113021	0.072914	0.053397	0.083190	0.094348	0.096865	0.107255	0.150602	0.164858
XX	Huilliche	0.074469	0.062724	0.065860	0.095323	0.062968	0.054200	0.063498	0.045293	0.055131	0.080766	0.105935	0.119139	0.102918	0.085815	0.092995	0.067013	0.068178	0.046863	0.052397	0.000000	0.112179	0.077434	0.057178	0.090624	0.105380	0.108146	0.112084	0.161278	0.165236
XXI	Kaingang	0.132014	0.124311	0.124288	0.153304	0.123748	0.114591	0.122041	0.102827	0.116198	0.140551	0.160109	0.170458	0.162654	0.143931	0.150461	0.123087	0.123546	0.107588	0.113021	0.112179	0.000000	0.133882	0.109996	0.144385	0.154920	0.164684	0.164829	0.214367	0.222456
XXII	Guarani	0.097329	0.083427	0.087849	0.120011	0.083918	0.075433	0.084530	0.065398	0.076494	0.102038	0.126726	0.142506	0.123804	0.104836	0.120183	0.089472	0.080429	0.064798	0.072914	0.077434	0.133882	0.000000	0.078947	0.107234	0.121087	0.121550	0.129712	0.174012	0.183146
XXIII	Wayuu	0.075862	0.064925	0.066329	0.095853	0.062683	0.054899	0.064529	0.046032	0.057038	0.071780	0.098967	0.108035	0.090087	0.078971	0.092406	0.061299	0.064456	0.048710	0.053397	0.057178	0.109996	0.078947	0.000000	0.088762	0.100132	0.105636	0.112161	0.163087	0.168365
XXIV	Piapoco	0.110321	0.100466	0.100428	0.128092	0.097240	0.089271	0.099444	0.076760	0.084250	0.112333	0.137862	0.144690	0.133754	0.116077	0.126301	0.096558	0.092527	0.077769	0.083190	0.090624	0.144385	0.107234	0.088762	0.000000	0.122059	0.126882	0.133396	0.184817	0.198950
XXV	Ticuna (Tarapaca)	0.125726	0.114931	0.119482	0.144225	0.112465	0.102664	0.108557	0.095510	0.099073	0.129046	0.151019	0.156653	0.141959	0.128265	0.138579	0.113896	0.099760	0.086344	0.094348	0.105380	0.154920	0.121087	0.100132	0.122059	0.000000	0.103470	0.146779	0.202118	0.207901
XXVI	Ticuna (Arara)	0.129276	0.114683	0.117823	0.146178	0.109831	0.103080	0.110001	0.094622	0.102185	0.131545	0.152282	0.157957	0.146717	0.134757	0.145801	0.112479	0.108760	0.091566	0.096865	0.108146	0.164684	0.121550	0.105636	0.126882	0.103470	0.000000	0.156172	0.202133	0.215785
XXVII	Karitiana	0.136513	0.125985	0.128860	0.154633	0.119573	0.112624	0.120581	0.099039	0.111190	0.133625	0.159341	0.165076	0.153646	0.141420	0.150893	0.119369	0.116566	0.100828	0.107255	0.112084	0.164829	0.129712	0.112161	0.133396	0.146779	0.156172	0.000000	0.209182	0.218687
XXVIII	Surui	0.186265	0.169458	0.172977	0.191110	0.164825	0.160643	0.166425	0.145336	0.158815	0.181968	0.207310	0.220948	0.198082	0.183842	0.200435	0.169388	0.165930	0.147883	0.150602	0.161278	0.214367	0.174012	0.163087	0.184817	0.202118	0.202133	0.209182	0.000000	0.268656
XXIX	Ache	0.193330	0.178319	0.184378	0.216433	0.176121	0.166150	0.180306	0.157583	0.165477	0.197354	0.219675	0.228489	0.220502	0.193093	0.209300	0.176929	0.168471	0.156493	0.164858	0.165236	0.222456	0.183146	0.168365	0.198950	0.207901	0.215785	0.218687	0.268656	0.000000
