# Supplementary material for: Genetic Variation and Population Structure in Native Americans
Source: PLoS Genet. 2007 Nov 23;3(11):e185. doi: 10.1371/journal.pgen.0030185 (PMC2082466; doi:10.1371/journal.pgen.0030185)
Supplement: Supplementary file 49 [file pgen.0030185.st029.pdf]

Table S29. Chord genetic distance matrix for Native American populations.

		I	II	III	IV	V	VI	VII	VIII	IX	X	XI	XII	XIII	XIV	XV	XVI	XVII	XVIII	XIX	XX	XXI	XXII	XXIII	XXIV	XXV	XXVI	XXVII	XXVIII	XXIX
I	Chipewyan	0.000000	0.264029	0.268400	0.349163	0.306662	0.291177	0.311256	0.267961	0.307346	0.329019	0.379549	0.386892	0.365707	0.346167	0.369181	0.319696	0.312994	0.273313	0.290884	0.288407	0.405066	0.344409	0.293258	0.362392	0.372962	0.375486	0.387429	0.449473	0.463971
II	Cree	0.264029	0.000000	0.243889	0.337766	0.290914	0.276371	0.292068	0.248119	0.297167	0.318264	0.362202	0.381925	0.351826	0.331989	0.356151	0.308553	0.301853	0.256502	0.277207	0.274097	0.395926	0.329342	0.283287	0.352266	0.361717	0.362794	0.377724	0.435561	0.449299
III	Ojibwa	0.268400	0.243889	0.000000	0.340361	0.293115	0.279024	0.295282	0.250089	0.292944	0.315688	0.359156	0.379818	0.349617	0.330912	0.357056	0.302595	0.300217	0.260587	0.279424	0.276007	0.395788	0.334695	0.282643	0.350782	0.366810	0.366422	0.380778	0.438452	0.454149
IV	Pima	0.349163	0.337766	0.340361	0.000000	0.336324	0.319966	0.336341	0.300325	0.336503	0.362591	0.409727	0.421786	0.410352	0.380350	0.407357	0.352473	0.351196	0.308988	0.320504	0.328970	0.454011	0.390018	0.336183	0.396505	0.411627	0.413295	0.429771	0.483965	0.518751
V	Mixtec	0.306662	0.290914	0.293115	0.336324	0.000000	0.259214	0.263917	0.244802	0.277835	0.305515	0.352410	0.366564	0.351666	0.325963	0.352296	0.295450	0.293917	0.254917	0.265491	0.273890	0.398292	0.332681	0.277710	0.341571	0.354442	0.351313	0.370243	0.432551	0.449224
VI	Zapotec	0.291177	0.276371	0.279024	0.319966	0.259214	0.000000	0.259664	0.232222	0.264070	0.296429	0.341473	0.355306	0.343680	0.317016	0.340847	0.283797	0.279516	0.242544	0.253304	0.258605	0.383710	0.319171	0.265193	0.332072	0.343913	0.343041	0.359888	0.423803	0.433963
VII	Mixe	0.311256	0.292068	0.295282	0.336341	0.263917	0.259664	0.000000	0.243096	0.277194	0.308464	0.349020	0.364933	0.357340	0.323237	0.356123	0.293639	0.292600	0.249688	0.263384	0.273395	0.400887	0.331936	0.279940	0.345775	0.350277	0.354629	0.370374	0.435776	0.456217
VIII	Maya	0.267961	0.248119	0.250089	0.300325	0.244802	0.232222	0.243096	0.000000	0.237706	0.272384	0.313098	0.330997	0.317230	0.288771	0.314862	0.259586	0.256375	0.210464	0.225082	0.236089	0.358698	0.293543	0.243457	0.304312	0.319591	0.322626	0.331653	0.393129	0.411142
IX	Kaqchikel	0.307346	0.297167	0.292944	0.336503	0.277835	0.264070	0.277194	0.237706	0.000000	0.300499	0.345284	0.364713	0.352089	0.322376	0.353080	0.292355	0.286726	0.244757	0.259720	0.271535	0.403854	0.328760	0.278584	0.340012	0.350506	0.355272	0.372884	0.438520	0.451801
X	Cabecar	0.329019	0.318264	0.315688	0.362591	0.305515	0.296429	0.308464	0.272384	0.300499	0.000000	0.314610	0.362615	0.355619	0.322626	0.350423	0.302154	0.314833	0.279862	0.289461	0.297304	0.418149	0.349480	0.289453	0.363012	0.375809	0.379440	0.386069	0.448703	0.471200
XI	Guaymi	0.379549	0.362202	0.359156	0.409727	0.352410	0.341473	0.349020	0.313098	0.345284	0.314610	0.000000	0.398843	0.404030	0.367288	0.403406	0.346237	0.359974	0.322602	0.335396	0.338754	0.461883	0.397633	0.334522	0.410903	0.418425	0.419929	0.431388	0.497778	0.516400
XII	Kogi	0.386892	0.381925	0.379818	0.421786	0.366564	0.355306	0.364933	0.330997	0.364713	0.362615	0.398843	0.000000	0.380760	0.393086	0.424009	0.368520	0.378214	0.340574	0.352210	0.359832	0.474168	0.415133	0.350499	0.422282	0.428224	0.427312	0.441104	0.519001	0.534395
XIII	Arhuaco	0.365707	0.351826	0.349617	0.410352	0.351666	0.343680	0.357340	0.317230	0.352089	0.355619	0.404030	0.380760	0.000000	0.382634	0.418223	0.354474	0.359460	0.323726	0.337880	0.342579	0.465587	0.401520	0.332364	0.412186	0.414616	0.419837	0.433542	0.497416	0.520697
XIV	Waunana	0.346167	0.331989	0.330912	0.380350	0.325963	0.317016	0.323237	0.288771	0.322376	0.322626	0.367288	0.393086	0.382634	0.000000	0.366829	0.327955	0.329398	0.291810	0.302232	0.311442	0.435447	0.367689	0.310936	0.377172	0.387890	0.392572	0.404396	0.465948	0.482561
XV	Embera	0.369181	0.356151	0.357056	0.407357	0.352296	0.340847	0.356123	0.314862	0.353080	0.350423	0.403406	0.424009	0.418223	0.366829	0.000000	0.354069	0.356392	0.322135	0.335747	0.336055	0.462017	0.402070	0.340018	0.410211	0.414400	0.419926	0.432710	0.506379	0.521164
XVI	Zenu	0.319696	0.308553	0.302595	0.352473	0.295450	0.283797	0.293639	0.259586	0.292355	0.302154	0.346237	0.368520	0.354474	0.327955	0.354069	0.000000	0.301735	0.263306	0.278419	0.284106	0.406342	0.342734	0.280723	0.350688	0.364985	0.368143	0.376516	0.444677	0.456491
XVII	Inga	0.312994	0.301853	0.300217	0.351196	0.293917	0.279516	0.292600	0.256375	0.286726	0.314833	0.359974	0.378214	0.359460	0.329398	0.356392	0.301735	0.000000	0.251691	0.268650	0.280939	0.405453	0.330883	0.284280	0.343765	0.345567	0.356558	0.372360	0.437429	0.448231
XVIII	Quechua	0.273313	0.256502	0.260587	0.308988	0.254917	0.242544	0.249688	0.210464	0.244757	0.279862	0.322602	0.340574	0.323726	0.291810	0.322135	0.263306	0.251691	0.000000	0.211004	0.237664	0.366413	0.292600	0.247685	0.306821	0.311275	0.318874	0.335672	0.397055	0.411499
XIX	Aymara	0.290884	0.277207	0.279424	0.320504	0.265491	0.253304	0.263384	0.225082	0.259720	0.289461	0.335396	0.352210	0.337880	0.302232	0.335747	0.278419	0.268650	0.211004	0.000000	0.251663	0.383081	0.313257	0.261955	0.323139	0.332527	0.335417	0.354820	0.412453	0.435380
XX	Huilliche	0.288407	0.274097	0.276007	0.328970	0.273890	0.258605	0.273395	0.236089	0.271535	0.297304	0.338754	0.359832	0.342579	0.311442	0.336055	0.284106	0.280939	0.237664	0.251663	0.000000	0.376683	0.314779	0.264709	0.328917	0.341894	0.343111	0.353114	0.417608	0.424928
XXI	Kaingang	0.405066	0.395926	0.395788	0.454011	0.398292	0.383710	0.400887	0.358698	0.403854	0.418149	0.461883	0.474168	0.465587	0.435447	0.462017	0.406342	0.405453	0.366413	0.383081	0.376683	0.000000	0.437516	0.380001	0.449775	0.453453	0.465205	0.474305	0.550770	0.559834
XXII	Guarani	0.344409	0.329342	0.334695	0.390018	0.332681	0.319171	0.331936	0.293543	0.328760	0.349480	0.397633	0.415133	0.401520	0.367689	0.402070	0.342734	0.330883	0.292600	0.313257	0.314779	0.437516	0.000000	0.321324	0.383083	0.396139	0.394599	0.405379	0.469253	0.483333
XXIII	Wayuu	0.293258	0.283287	0.282643	0.336183	0.277710	0.265193	0.279940	0.243457	0.278584	0.289453	0.334522	0.350499	0.332364	0.310936	0.340018	0.280723	0.284280	0.247685	0.261955	0.264709	0.380001	0.321324	0.000000	0.334547	0.340346	0.348238	0.360987	0.424873	0.437212
XXIV	Piapoco	0.362392	0.352266	0.350782	0.396505	0.341571	0.332072	0.345775	0.304312	0.340012	0.363012	0.410903	0.422282	0.412186	0.377172	0.410211	0.350688	0.343765	0.306821	0.323139	0.328917	0.449775	0.383083	0.334547	0.000000	0.391275	0.396632	0.408994	0.487113	0.504891
XXV	Ticuna (Tarapaca)	0.372962	0.361717	0.366810	0.411627	0.354442	0.343913	0.350277	0.319591	0.350506	0.375809	0.418425	0.428224	0.414616	0.387890	0.414400	0.364985	0.345567	0.311275	0.332527	0.341894	0.453453	0.396139	0.340346	0.391275	0.000000	0.349508	0.419809	0.495918	0.511123
XXVI	Ticuna (Arara)	0.375486	0.362794	0.366422	0.413295	0.351313	0.343041	0.354629	0.322626	0.355272	0.379440	0.419929	0.427312	0.419837	0.392572	0.419926	0.368143	0.356558	0.318874	0.335417	0.343111	0.465205	0.394599	0.348238	0.396632	0.349508	0.000000	0.432483	0.496135	0.515314
XXVII	Karitiana	0.387429	0.377724	0.380778	0.429771	0.370243	0.359888	0.370374	0.331653	0.372884	0.386069	0.431388	0.441104	0.433542	0.404396	0.432710	0.376516	0.372360	0.335672	0.354820	0.353114	0.474305	0.405379	0.360987	0.408994	0.419809	0.432483	0.000000	0.506816	0.530076
XXVIII	Surui	0.449473	0.435561	0.438452	0.483965	0.432551	0.423803	0.435776	0.393129	0.438520	0.448703	0.497778	0.519001	0.497416	0.465948	0.506379	0.444677	0.437429	0.397055	0.412453	0.417608	0.550770	0.469253	0.424873	0.487113	0.495918	0.496135	0.506816	0.000000	0.607841
XXIX	Ache	0.463971	0.449299	0.454149	0.518751	0.449224	0.433963	0.456217	0.411142	0.451801	0.471200	0.516400	0.534395	0.520697	0.482561	0.521164	0.456491	0.448231	0.411499	0.435380	0.424928	0.559834	0.483333	0.437212	0.504891	0.511123	0.515314	0.530076	0.607841	0.000000
