# Supplementary material for: Genetic Variation and Population Structure in Native Americans
Source: PLoS Genet. 2007 Nov 23;3(11):e185. doi: 10.1371/journal.pgen.0030185 (PMC2082466; doi:10.1371/journal.pgen.0030185)
Supplement: Supplementary file 50 [file pgen.0030185.st030.pdf]

Table S30. Waypoint geographic distance matrix for Native American populations, in kilometers.

		I	II	III	IV	V	VI	VII	VIII	IX	X	XI	XII	XIII	XIV	XV	XVI	XVII	XVIII	XIX	XX	XXI	XXII	XXIII	XXIV	XXV	XXVI	XXVII	XXVIII	XXIX
I	Chipewyan	0	1069	2254	3397	4804	4915	4819	4695	5130	5897	6061	6063	6071	6590	6412	6236	7016	8709	9683	11615	10542	10378	6101	7128	7784	7784	8676	8819	10397
II	Cree	1069	0	1637	2416	3739	3849	3751	3629	4063	4849	5021	5089	5098	5574	5408	5246	5991	7674	8654	10554	9614	9442	5136	6177	6793	6793	7720	7867	9444
III	Ojibwa	2254	1637	0	3043	3596	3701	3560	3192	3624	4124	4226	4002	4006	4631	4418	4209	5074	6764	7697	9762	8348	8192	4019	4998	5719	5719	6533	6668	8233
IV	Pima	3397	2416	3043	0	1744	1832	1811	2050	2339	3310	3545	4054	4072	4214	4150	4096	4527	6016	6981	8577	8362	8174	4144	5117	5468	5468	6487	6643	8102
V	Mixtec	4804	3739	3596	1744	0	111	106	672	679	1634	1880	2567	2588	2554	2537	2541	2820	4273	5239	6908	6641	6452	2672	3528	3774	3774	4794	4949	6370
VI	Zapotec	4915	3849	3701	1832	111	0	154	718	652	1583	1830	2546	2567	2502	2495	2510	2755	4185	5148	6804	6566	6378	2652	3486	3710	3710	4729	4883	6292
VII	Mixe	4819	3751	3560	1811	106	154	0	574	579	1542	1787	2464	2484	2462	2440	2440	2736	4209	5179	6872	6562	6374	2567	3430	3689	3689	4709	4864	6295
VIII	Maya	4695	3629	3192	2050	672	718	574	0	445	1297	1518	2029	2048	2174	2100	2051	2517	4115	5099	6926	6346	6160	2126	3069	3439	3439	4452	4608	6104
IX	Kaqchikel	5130	4063	3624	2339	679	652	579	445	0	976	1217	1894	1915	1892	1862	1863	2188	3728	4708	6497	6024	5836	1999	2852	3133	3133	4153	4309	5767
X	Cabecar	5897	4849	4124	3310	1634	1583	1542	1297	976	0	246	1107	1128	920	923	989	1222	2836	3821	5727	5058	4870	1215	1910	2159	2159	3178	3334	4809
XI	Guaymi	6061	5021	4226	3545	1880	1830	1787	1518	1217	246	0	920	941	675	682	771	1001	2653	3635	5581	4829	4642	1025	1665	1924	1924	2942	3098	4586
XII	Kogi	6063	5089	4002	4054	2567	2546	2464	2029	1894	1107	920	0	22	744	496	248	1160	2780	3695	5783	4539	4363	109	1109	1726	1726	2633	2783	4356
XIII	Arhuaco	6071	5098	4006	4072	2588	2567	2484	2048	1915	1128	941	22	0	754	506	258	1167	2780	3693	5783	4528	4352	87	1096	1720	1720	2623	2772	4346
XIV	Waunana	6590	5574	4631	4214	2554	2502	2462	2174	1892	920	675	744	754	0	248	497	445	2138	3097	5131	4177	3992	799	1023	1267	1267	2277	2433	3947
XV	Embera	6412	5408	4418	4150	2537	2495	2440	2100	1862	923	682	496	506	248	0	248	676	2346	3290	5346	4288	4105	553	991	1393	1393	2376	2531	4073
XVI	Zenu	6236	5246	4209	4096	2541	2510	2440	2051	1863	989	771	248	258	497	248	0	917	2560	3490	5564	4409	4229	312	1022	1548	1548	2495	2648	4210
XVII	Inga	7016	5991	5074	4527	2820	2755	2736	2517	2188	1222	1001	1160	1167	445	676	917	0	1700	2668	4688	3837	3649	1196	1025	956	956	1974	2129	3587
XVIII	Quechua	8709	7674	6764	6016	4273	4185	4209	4115	3728	2836	2653	2780	2780	2138	2346	2560	1700	0	985	3004	2514	2331	2782	2003	1195	1195	1276	1344	2191
XIX	Aymara	9683	8654	7697	6981	5239	5148	5179	5099	4708	3821	3635	3695	3693	3097	3290	3490	2668	985	0	2131	1804	1647	3684	2788	2002	2002	1529	1490	1449
XX	Huilliche	11615	10554	9762	8577	6908	6804	6872	6926	6497	5727	5581	5783	5783	5131	5346	5564	4688	3004	2131	0	2683	2674	5782	4919	4125	4125	3584	3505	2463
XXI	Kaingang	10542	9614	8348	8362	6641	6566	6562	6346	6024	5058	4829	4539	4528	4177	4288	4409	3837	2514	1804	2683	0	189	4484	3439	2910	2910	1914	1760	356
XXII	Guarani	10378	9442	8192	8174	6452	6378	6374	6160	5836	4870	4642	4363	4352	3992	4105	4229	3649	2331	1647	2674	189	0	4310	3266	2725	2725	1734	1581	232
XXIII	Wayuu	6101	5136	4019	4144	2672	2652	2567	2126	1999	1215	1025	109	87	799	553	312	1196	2782	3684	5782	4484	4310	0	1047	1701	1701	2584	2732	4308
XXIV	Piapoco	7128	6177	4998	5117	3528	3486	3430	3069	2852	1910	1665	1109	1096	1023	991	1022	1025	2003	2788	4919	3439	3266	1047	0	809	809	1548	1692	3271
XXV	Ticuna (Tarapaca)	7784	6793	5719	5468	3774	3710	3689	3439	3133	2159	1924	1726	1720	1267	1393	1548	956	1195	2002	4125	2910	2725	1701	809	0	0	1020	1176	2683
XXVI	Ticuna (Arara)	7784	6793	5719	5468	3774	3710	3689	3439	3133	2159	1924	1726	1720	1267	1393	1548	956	1195	2002	4125	2910	2725	1701	809	0	0	1020	1176	2683
XXVII	Karitiana	8676	7720	6533	6487	4794	4729	4709	4452	4153	3178	2942	2633	2623	2277	2376	2495	1974	1276	1529	3584	1914	1734	2584	1548	1020	1020	0	156	1724
XXVIII	Surui	8819	7867	6668	6643	4949	4883	4864	4608	4309	3334	3098	2783	2772	2433	2531	2648	2129	1344	1490	3505	1760	1581	2732	1692	1176	1176	156	0	1579
XXIX	Ache	10397	9444	8233	8102	6370	6292	6295	6104	5767	4809	4586	4356	4346	3947	4073	4210	3587	2191	1449	2463	356	232	4308	3271	2683	2683	1724	1579	0
