# Supplementary material for: Genetic Variation and Population Structure in Native Americans
Source: PLoS Genet. 2007 Nov 23;3(11):e185. doi: 10.1371/journal.pgen.0030185 (PMC2082466; doi:10.1371/journal.pgen.0030185)
Supplement: Supplementary file 51 [file pgen.0030185.st031.pdf]

Table S31. Heterozygosity and F_{ST} (×100) for various geographic regions, based on a reduced data set of 1306 individuals.

Geographic region	Number of populations	Heterozygosity (pooled)	Heterozygosity (average across populations)	F_{ST} (×100)
Worldwide	78	0.742 (0.072)	0.688 (0.076)	6.1 (5.8, 6.3)
Africa	7	0.775 (0.072)	0.758 (0.073)	2.6 (2.4, 2.8)
Europe	8	0.732 (0.075)	0.728 (0.076)	0.8 (0.6, 0.9)
Middle East	4	0.740 (0.073)	0.733 (0.072)	1.3 (1.2, 1.4)
Central/South Asia	9	0.738 (0.076)	0.730 (0.076)	1.2 (1.1, 1.4)
East Asia	19	0.714 (0.098)	0.705 (0.097)	1.3 (1.1, 1.4)
Oceania	2	0.694 (0.120)	0.674 (0.123)	5.6 (4.9, 6.3)
America	29	0.677 (0.111)	0.630 (0.105)	6.3 (6.1, 6.5)
North America	3	0.699 (0.100)	0.688 (0.101)	2.5 (2.2, 2.9)
Central America	8	0.669 (0.119)	0.641 (0.116)	4.6 (4.3, 4.9)
Western South America	10	0.675 (0.113)	0.643 (0.110)	4.0 (3.7, 4.2)
Eastern South America	8	0.642 (0.131)	0.582 (0.118)	12.6 (12.1, 13.1)

Heterozygosities are listed with standard deviations across loci. Pooled heterozygosity is significantly lower in Native Americans than in any of the other major geographic regions ($P<0.0001$ for each comparison, Wilcoxon signed rank test). For convenience, values of 100 times F_{ST} are shown in the table rather than F_{ST} values, and they are listed with 95% bootstrap confidence intervals across loci. The populations included in the various subdivisions of the Americas are listed in Table 2.
