# Supplementary material for: Genetic Variation and Population Structure in Native Americans
Source: PLoS Genet. 2007 Nov 23;3(11):e185. doi: 10.1371/journal.pgen.0030185 (PMC2082466; doi:10.1371/journal.pgen.0030185)
Supplement: Supplementary file 52 [file pgen.0030185.st032.pdf]

Table S32. Correlation of genetic and linguistic distances, based on a reduced set of 1306 individuals.

Population comparison	Distance measure between stocks	Excluded stock	Correlation of genetic and linguistic distance		Partial correlation of genetic and linguistic distance controlling for geographic distance	
			r	*P*	*r*	*P*
All pairs	Equal		0.03	0.23	0.01	0.43
All pairs excluding one stock	Equal	Northern Amerind	0.05	0.21	0.04	0.29
	Equal	Central Amerind	0.05	0.16	0.05	0.22
	Equal	Chibchan-Paezan	0.01	0.44	-0.02	0.64
	Equal	Andean	0.04	0.26	-0.05	0.77
	Equal	Equatorial-Tucanoan	0.15	0.004	0.13	0.02
All pairs	Dice		-0.03	0.63	-0.09	0.82
All pairs excluding one stock	Dice	Northern Amerind	-0.12	0.90	-0.16	0.96
	Dice	Central Amerind	-0.04	0.66	-0.05	0.69
	Dice	Chibchan-Paezan	-0.06	0.75	-0.14	0.92
	Dice	Andean	0.13	0.04	-0.04	0.69
	Dice	Equatorial-Tucanoan	-0.004	0.51	-0.08	0.71
All pairs	Jaccard		-0.04	0.66	-0.09	0.84
All pairs excluding one stock	Jaccard	Northern Amerind	-0.12	0.90	-0.15	0.96
	Jaccard	Central Amerind	-0.04	0.68	-0.06	0.72
	Jaccard	Chibchan-Paezan	-0.07	0.76	-0.15	0.94
	Jaccard	Andean	0.14	0.03	-0.04	0.67
	Jaccard	Equatorial-Tucanoan	-0.001	0.49	-0.08	0.70
All pairs within stocks	-		0.51	0.16	0.39	0.16
All pairs within stocks excluding one stock	-	Northern Amerind	0.51	0.17	0.39	0.16
	-	Central Amerind	0.49	0.18	0.38	0.19
	-	Chibchan-Paezan	0.55	0.07	0.40	0.06
	-	Andean	0.44	0.42	0.25	0.43
	-	Equatorial-Tucanoan	0.66	0.02	0.65	0.03
