# Supplementary material for: Genetic Variation and Population Structure in Native Americans
Source: PLoS Genet. 2007 Nov 23;3(11):e185. doi: 10.1371/journal.pgen.0030185 (PMC2082466; doi:10.1371/journal.pgen.0030185)
Supplement: Supplementary file 53 [file pgen.0030185.st033.pdf]

Table S33. Nei genetic distance matrix for Native American populations, based on a reduced set of 1306 individuals.

		I	II	III	IV	V	VI	VII	VIII	IX	X	XI	XII	XIII	XIV	XV	XVI	XVII	XVIII	XIX	XX	XXI	XXII	XXIII	XXIV	XXV	XXVI	XXVII	XXVIII	XXIX
I	Chipewyan	0.000000	0.092455	0.103464	0.147480	0.122356	0.112084	0.122375	0.099494	0.120042	0.140045	0.174602	0.195137	0.172456	0.144702	0.169899	0.135495	0.123172	0.101119	0.111063	0.112069	0.192825	0.149388	0.114788	0.167251	0.170706	0.171071	0.179276	0.229858	0.246927
II	Cree	0.092455	0.000000	0.085719	0.136204	0.107226	0.098948	0.106258	0.082758	0.108510	0.130796	0.158660	0.190177	0.162198	0.130425	0.156555	0.124733	0.114263	0.086312	0.097908	0.097930	0.183726	0.134119	0.105100	0.156782	0.160330	0.160211	0.167074	0.220864	0.231700
III	Ojibwa	0.103464	0.085719	0.000000	0.141679	0.116718	0.109098	0.116210	0.092447	0.115205	0.136148	0.161045	0.194592	0.167758	0.138320	0.165099	0.127069	0.119410	0.096474	0.107352	0.108781	0.189523	0.145295	0.113474	0.161316	0.168988	0.169321	0.175282	0.225617	0.239993
IV	Pima	0.147480	0.136204	0.141679	0.000000	0.123548	0.115261	0.120433	0.109340	0.118710	0.144694	0.167576	0.188797	0.181791	0.148011	0.173456	0.139048	0.132107	0.112387	0.113985	0.127099	0.197342	0.158638	0.131170	0.162911	0.167716	0.168324	0.175105	0.214777	0.242990
V	Mixtec	0.122356	0.107226	0.116718	0.123548	0.000000	0.082275	0.079605	0.077238	0.089081	0.112606	0.136735	0.163291	0.152803	0.119058	0.145551	0.105948	0.101177	0.082252	0.085024	0.094485	0.171790	0.130920	0.095043	0.136030	0.137588	0.136992	0.146638	0.196311	0.209350
VI	Zapotec	0.112084	0.098948	0.109098	0.115261	0.082275	0.000000	0.080801	0.071315	0.083609	0.110328	0.134757	0.159613	0.150613	0.118472	0.140306	0.101941	0.094591	0.077157	0.079754	0.086185	0.166180	0.124336	0.089252	0.134795	0.136676	0.136399	0.145461	0.195505	0.205092
VII	Mixe	0.122375	0.106258	0.116210	0.120433	0.079605	0.080801	0.000000	0.075627	0.086957	0.111637	0.130843	0.156503	0.151276	0.114515	0.144078	0.101008	0.097643	0.076896	0.081649	0.091943	0.168769	0.125591	0.094051	0.135461	0.131800	0.136762	0.142229	0.192596	0.209241
VIII	Maya	0.099494	0.082758	0.092447	0.109340	0.077238	0.071315	0.075627	0.000000	0.071992	0.098519	0.121151	0.149605	0.135724	0.101535	0.127932	0.090920	0.083651	0.060094	0.065974	0.074545	0.154512	0.111517	0.078861	0.121777	0.126973	0.129249	0.131597	0.183481	0.197858
IX	Kaqchikel	0.120042	0.108510	0.115205	0.118710	0.089081	0.083609	0.086957	0.071992	0.000000	0.105099	0.125642	0.154043	0.146203	0.114119	0.140240	0.101520	0.092774	0.074011	0.078597	0.090882	0.168604	0.122138	0.094304	0.131113	0.132390	0.134845	0.142174	0.190700	0.199845
X	Cabecar	0.140045	0.130796	0.136148	0.144694	0.112606	0.110328	0.111637	0.098519	0.105099	0.000000	0.106306	0.154886	0.152467	0.116821	0.142215	0.110803	0.116792	0.101076	0.102677	0.113603	0.186983	0.143144	0.104115	0.154259	0.156538	0.160174	0.157815	0.209585	0.228749
XI	Guaymi	0.174602	0.158660	0.161045	0.167576	0.136735	0.134757	0.130843	0.121151	0.125642	0.106306	0.000000	0.164348	0.175434	0.138450	0.168492	0.132187	0.140370	0.125251	0.127168	0.136200	0.201432	0.168308	0.128095	0.175282	0.173049	0.175441	0.175093	0.221032	0.233657
XII	Kogi	0.195137	0.190177	0.194592	0.188797	0.163291	0.159613	0.156503	0.149605	0.154043	0.154886	0.164348	0.000000	0.172545	0.168070	0.192459	0.159567	0.167429	0.154276	0.153559	0.166012	0.219004	0.190069	0.155712	0.192626	0.188153	0.192601	0.191250	0.244686	0.254063
XIII	Arhuaco	0.172456	0.162198	0.167758	0.181791	0.152803	0.150613	0.151276	0.135724	0.146203	0.152467	0.175434	0.172545	0.000000	0.164987	0.196421	0.154235	0.150653	0.138115	0.142237	0.151242	0.223100	0.183563	0.141935	0.190934	0.183914	0.190957	0.195680	0.241850	0.259815
XIV	Waunana	0.144702	0.130425	0.138320	0.148011	0.119058	0.118472	0.114515	0.101535	0.114119	0.116821	0.138450	0.168070	0.164987	0.000000	0.143692	0.121196	0.120230	0.103217	0.106113	0.116492	0.190297	0.145831	0.111878	0.154027	0.156811	0.159917	0.165283	0.208706	0.222589
XV	Embera	0.169899	0.156555	0.165099	0.173456	0.145551	0.140306	0.144078	0.127932	0.140240	0.142215	0.168492	0.192459	0.196421	0.143692	0.000000	0.146031	0.143693	0.130230	0.132454	0.138988	0.209444	0.175103	0.138637	0.180217	0.175044	0.181411	0.184621	0.233864	0.248103
XVI	Zenu	0.135495	0.124733	0.127069	0.139048	0.105948	0.101941	0.101008	0.090920	0.101520	0.110803	0.132187	0.159567	0.154235	0.121196	0.146031	0.000000	0.108950	0.090707	0.095990	0.104169	0.175173	0.135810	0.100496	0.142900	0.144976	0.149731	0.152173	0.204082	0.210618
XVII	Inga	0.123172	0.114263	0.119410	0.132107	0.101177	0.094591	0.097643	0.083651	0.092774	0.116792	0.140370	0.167429	0.150653	0.120230	0.143693	0.108950	0.000000	0.078984	0.085354	0.097754	0.171152	0.125481	0.098476	0.133101	0.128225	0.138472	0.144905	0.192584	0.200212
XVIII	Quechua	0.101119	0.086312	0.096474	0.112387	0.082252	0.077157	0.076896	0.060094	0.074011	0.101076	0.125251	0.154276	0.138115	0.103217	0.130230	0.090707	0.078984	0.000000	0.055719	0.073958	0.157477	0.108351	0.079593	0.120711	0.116956	0.122933	0.131475	0.181844	0.194389
XIX	Aymara	0.111063	0.097908	0.107352	0.113985	0.085024	0.079754	0.081649	0.065974	0.078597	0.102677	0.127168	0.153559	0.142237	0.106113	0.132454	0.095990	0.085354	0.055719	0.000000	0.080125	0.161091	0.116628	0.084988	0.126207	0.126366	0.128247	0.138093	0.182008	0.202749
XX	Huilliche	0.112069	0.097930	0.108781	0.127099	0.094485	0.086185	0.091943	0.074545	0.090882	0.113603	0.136200	0.166012	0.151242	0.116492	0.138988	0.104169	0.097754	0.073958	0.080125	0.000000	0.164649	0.122324	0.090808	0.135029	0.140769	0.141294	0.144502	0.198709	0.204502
XXI	Kaingang	0.192825	0.183726	0.189523	0.197342	0.171790	0.166180	0.168769	0.154512	0.168604	0.186983	0.201432	0.219004	0.223100	0.190297	0.209444	0.175173	0.171152	0.157477	0.161091	0.164649	0.000000	0.189689	0.161720	0.196351	0.191001	0.203438	0.201025	0.252229	0.247729
XXII	Guarani	0.149388	0.134119	0.145295	0.158638	0.130920	0.124336	0.125591	0.111517	0.122138	0.143144	0.168308	0.190069	0.183563	0.145831	0.175103	0.135810	0.125481	0.108351	0.116628	0.122324	0.189689	0.000000	0.126216	0.157598	0.161830	0.163555	0.164707	0.205731	0.215734
XXIII	Wayuu	0.114788	0.105100	0.113474	0.131170	0.095043	0.089252	0.094051	0.078861	0.094304	0.104115	0.128095	0.155712	0.141935	0.111878	0.138637	0.100496	0.098476	0.079593	0.084988	0.090808	0.161720	0.126216	0.000000	0.136978	0.133684	0.140654	0.146440	0.197060	0.210515
XXIV	Piapoco	0.167251	0.156782	0.161316	0.162911	0.136030	0.134795	0.135461	0.121777	0.131113	0.154259	0.175282	0.192626	0.190934	0.154027	0.180217	0.142900	0.133101	0.120711	0.126207	0.135029	0.196351	0.157598	0.136978	0.000000	0.155045	0.159262	0.161792	0.210746	0.224119
XXV	Ticuna (Tarapaca)	0.170706	0.160330	0.168988	0.167716	0.137588	0.136676	0.131800	0.126973	0.132390	0.156538	0.173049	0.188153	0.183914	0.156811	0.175044	0.144976	0.128225	0.116956	0.126366	0.140769	0.191001	0.161830	0.133684	0.155045	0.000000	0.111625	0.163449	0.214358	0.224238
XXVI	Ticuna (Arara)	0.171071	0.160211	0.169321	0.168324	0.136992	0.136399	0.136762	0.129249	0.134845	0.160174	0.175441	0.192601	0.190957	0.159917	0.181411	0.149731	0.138472	0.122933	0.128247	0.141294	0.203438	0.163555	0.140654	0.159262	0.111625	0.000000	0.176302	0.217335	0.230977
XXVII	Karitiana	0.179276	0.167074	0.175282	0.175105	0.146638	0.145461	0.142229	0.131597	0.142174	0.157815	0.175093	0.191250	0.195680	0.165283	0.184621	0.152173	0.144905	0.131475	0.138093	0.144502	0.201025	0.164707	0.146440	0.161792	0.163449	0.176302	0.000000	0.212483	0.231283
XXVIII	Surui	0.229858	0.220864	0.225617	0.214777	0.196311	0.195505	0.192596	0.183481	0.190700	0.209585	0.221032	0.244686	0.241850	0.208706	0.233864	0.204082	0.192584	0.181844	0.182008	0.198709	0.252229	0.205731	0.197060	0.210746	0.214358	0.217335	0.212483	0.000000	0.268376
XXIX	Ache	0.246927	0.231700	0.239993	0.242990	0.209350	0.205092	0.209241	0.197858	0.199845	0.228749	0.233657	0.254063	0.259815	0.222589	0.248103	0.210618	0.200212	0.194389	0.202749	0.204502	0.247729	0.215734	0.210515	0.224119	0.224238	0.230977	0.231283	0.268376	0.000000
